# Dietary and Lifestyle Correlates of Gut Microbiota Diversity and Composition in Healthy Adults: A Cross-Sectional Pilot Study Informing Longitudinal Research

**DOI:** 10.3390/nu18203325

**Published:** 2026-10-10

**Authors:** Massimiliano Guerra, Martina Brandolini, Laura Dionisi, Alessandra Mistral De Pascali, Ludovica Ingletto, Claudia Colosimo, Giulia Gatti, Maria Sofia Montanari, Anna Marzucco, Laura Grumiro, Giorgio Dirani, Silvia Zannoli, Alessandra Scagliarini, Monica Cricca, Vittorio Sambri

**Affiliations:** 1 Unit of Microbiology, The Greater Romagna Area Hub Laboratory, 47522 Cesena, Italy; martina.brandolini3@unibo.it (M.B.); giulia.gatti@auslromagna.it (G.G.); anna.marzucco@auslromagna.it (A.M.); giorgio.dirani@auslromagna.it (G.D.); silvia.zannoli@auslromagna.it (S.Z.); monica.cricca3@unibo.it (M.C.); vittorio.sambri@unibo.it (V.S.); 2 Department of Medical and Surgical Sciences (DIMEC), University of Bologna, 40138 Bologna, Italy; laura.dionisi2@unibo.it (L.D.); alessandra.depascal3@unibo.it (A.M.D.P.); ludovica.ingletto@unibo.it (L.I.); claudia.colosimo2@unibo.it (C.C.); mariasofia.montanar2@unibo.it (M.S.M.); laura.grumiro2@unibo.it (L.G.); alessand.scagliarini@unibo.it (A.S.)

**Keywords:** gut microbiota, Mediterranean diet, ultra-processed foods, physical activity, sleep, 16S rRNA gene sequencing, compositional data analysis, effect size, sample-size planning, longitudinal study design

## Abstract

Background/Objectives: Modifiable dietary and lifestyle factors may contribute to gut microbiota variation but are often evaluated separately. This pilot study examined four exposures across complementary microbiome domains and used association magnitude and uncertainty to inform longitudinal study design. Methods: Twenty healthy adults (10 females and 10 males) provided a fresh-stool specimen and completed the exposure questionnaire on the day of specimen production. Exposures comprised Mediterranean diet adherence, ultra-processed food consumption frequency, physical activity, and sleep characteristics. Microbiome outcomes comprised independently recalculated OTU-level Shannon diversity (primary outcome) and Pielou’s evenness, genus-level Bray–Curtis and Aitchison distances, centered log-ratio coordinates of eight prespecified genera, and the natural-log-transformed *Prevotella*/*Bacteroides* ratio. Associations were evaluated using age- and sex-adjusted linear regression and permutational multivariate analysis of variance, with false-discovery-rate control, influence diagnostics, and robustness analyses. Scenario-based sample-size calculations were restricted to age- and sex-adjusted cross-sectional linear regression models. Results: Complete exposure and microbiome data were available for all participants. No association was supported after false-discovery-rate correction. However, the observed effect estimates were not uniformly negligible: maximum partial *R*^2^ values were 0.140 across the alpha-diversity models, 0.210 for the UPF–*Akkermansia* association, and 0.108 for the *Prevotella*/*Bacteroides* log-ratio, whereas the maximum marginal *R*^2^ for overall community composition was 0.0649. Estimates were imprecise and participant-sensitive, and robustness analyses did not alter the overall interpretation. Conservative scenarios for age- and sex-adjusted cross-sectional regression models, based on assumed partial *R*^2^ values of 0.03–0.05, yielded planning benchmarks of 275–465 analyzable participants, or 306–582 enrolled participants assuming 10–20% unusable observations. Conclusions: This pilot does not establish the absence of diet–microbiota or lifestyle–microbiota relationships. Instead, it provides a reproducible framework for prioritizing hypotheses and planning adequately powered longitudinal studies using repeated, temporally aligned exposure and microbiome measurements.

## 1. Introduction

The human gut microbiota exhibits substantial inter-individual variability, even among apparently healthy individuals. Large population-based studies have identified demographic, anthropometric, dietary, behavioral, medication-related, and intestinal factors as correlates of microbial community structure; nevertheless, the variables measured in these studies collectively explain only a limited proportion of the overall variation [1,2]. This complexity makes it difficult to distinguish reproducible biological relationships from population-specific, confounded, or technically induced variation, particularly in small observational studies.

Diet is among the most relevant potentially modifiable determinants of the gut microbiota. Controlled feeding studies have demonstrated that marked dietary changes can rapidly and reproducibly alter microbial composition and metabolic activity [3]. Particular attention has been directed toward the Mediterranean dietary pattern, characterized by a high intake of plant-based foods and olive oil, moderate fish consumption, and limited intake of red and processed meat. Greater adherence to this pattern has been associated with differences in taxonomic composition, microbial diversity, and metabolite profiles [4]. In a recent study of 60 well-characterized healthy individuals, greater Mediterranean diet adherence was associated with higher alpha diversity and greater relative abundances of *Paraprevotella* and *Bacteroides* [5]. In a separate one-year randomized trial involving 400 older adults at high cardiovascular risk, an energy-reduced Mediterranean diet combined with physical activity increased alpha diversity, reduced the relative abundances of the *Eubacterium hallii* group and *Dorea*, and modified the fecal metabolome [6]. However, the magnitude and direction of diet–microbiota associations have not been uniform across populations or microbiome outcomes.

Ultra-processed food consumption represents a complementary dietary dimension of growing interest. In adults with overweight or obesity and metabolic syndrome, ultra-processed food intake was not associated with overall alpha or beta diversity but showed exploratory associations with *Alloprevotella*, *Negativibacillus*, *Prevotella*, and *Sutterella* under the study-specific false-discovery threshold [7]. More recently, a six-month randomized dietary intervention found that restricting ultra-processed foods was associated with greater increases in Ruminococcaceae and *Faecalibacterium*, while the overall microbial response remained limited and heterogeneous [8]. Physical activity may also contribute to microbiota variability: exercise interventions have modified microbial composition and function, although responses have differed according to host metabolic status and have not always persisted after exercise discontinuation [9]. Evidence regarding sleep is similarly heterogeneous. Recurrent partial sleep restriction has been associated with an increased Firmicutes-to-Bacteroidetes ratio and changes in Coriobacteriaceae, Erysipelotrichaceae, and Tenericutes, without evidence of a major shift in overall diversity [10]. In another randomized crossover study, severe short-term sleep restriction reduced microbial richness by 21% and altered only one amplicon sequence variant within Ruminococcaceae, without significant changes in Shannon diversity, Simpson diversity, or beta diversity [11]. These findings indicate that global diversity indices, community-level dissimilarities, and individual bacterial groups may capture distinct and not necessarily concordant dimensions of the microbial response.

Despite growing evidence, important gaps remain. Dietary and lifestyle exposures are frequently examined separately, although Mediterranean diet adherence, ultra-processed food consumption, physical activity, and sleep may be behaviorally interrelated. Moreover, much of the available evidence derives from intervention studies or populations with obesity, metabolic disorders, or advanced age. Controlled feeding research in young healthy adults has shown that repeated adoption and abandonment of a healthy dietary pattern can produce corresponding changes in microbial community composition [12]. Dense longitudinal sampling has also demonstrated marked day-to-day variation and highly personalized diet–microbiota relationships, with relatively few food–microbe associations shared consistently across individuals [13]. These observations emphasize the need for studies that integrate multiple modifiable exposures, distinguish global community properties from changes in specific bacterial groups, and separate cross-sectional inter-individual variation from within-person temporal dynamics.

Interpretation is further complicated by host characteristics and methodological heterogeneity. Age, sex, adiposity, medication use, bowel habits, and stool consistency may influence microbiota composition or confound observed associations [14]. Microbiome profiles are also sensitive to stool collection, storage, DNA extraction, sequencing, bioinformatic processing, and the statistical treatment of compositional data [15]. Standardized procedures and transparent reporting are therefore essential, as emphasized by the STORMS reporting guidelines for human microbiome research [16].

Small pilot datasets can nevertheless contribute meaningfully when used to characterize variability, estimate the magnitude and uncertainty of associations, identify influential observations, and evaluate an analytical workflow. Their purpose is not to demonstrate the absence of biologically relevant effects or to provide a single definitive sample-size target. Sample-size requirements depend on the selected microbiome outcome, expected effect size, population heterogeneity, covariate structure, multiple-testing strategy, and statistical method. Simulation-based approaches are particularly relevant for beta-diversity analyses using pairwise distances and permutational multivariate analysis of variance [17]. Recent evidence also indicates that temporal stability varies substantially across diversity measures and microbial features and that repeated sampling may improve the efficiency of future epidemiological studies [18]. Consequently, planning based on pilot data should compare multiple biologically plausible scenarios and account explicitly for uncertainty rather than rely exclusively on observed point estimates [19].

Against this background, the present observational cross-sectional pilot study integrated gut microbiome profiles with dietary and lifestyle data from the same 20 healthy adults to examine associations between four potentially modifiable exposures—Mediterranean Diet adherence, ultra-processed food consumption, physical activity, and sleep characteristics—and gut microbiota diversity and composition. Microbiome outcomes were organized within a hierarchical framework encompassing the Shannon diversity index and Pielou’s evenness, Bray–Curtis and Aitchison distances, eight prespecified bacterial genera (*Bacteroides*, *Prevotella*, *Faecalibacterium*, *Bifidobacterium*, *Blautia*, *Roseburia*, *Ruminococcus*, and *Akkermansia*), and the continuous *Prevotella*/*Bacteroides* compositional gradient. A complementary objective was to characterize the magnitude, direction, uncertainty, and robustness of the estimated associations, document a reproducible analytical workflow, and translate the information obtained from the pilot dataset into transparent sample-size planning scenarios and design considerations for future longitudinal studies involving larger cohorts, repeated and temporally aligned measurements, more detailed dietary assessment, and objective measures of physical activity and sleep.

## 2. Materials and Methods

### 2.1. Study Design and Reporting

The present investigation was designed as an observational, cross-sectional pilot study integrating gut microbiome profiles generated by 16S rRNA gene sequencing with demographic, dietary, physical-activity, and sleep data obtained from the same 20 healthy adults using structured questionnaires. The microbiome profiles originated from an earlier intra-individual methodological investigation in which multiple profiles had been generated from each participant to evaluate stool-collection and processing conditions. For the present study, one profile generated from fresh, non-homogenized, and non-diluted stool was retained per participant and integrated with the questionnaire-derived dataset. The individual participant constituted the unit of analysis, resulting in 20 independent microbiome observations and avoiding pseudoreplication from multiple profiles originating from the same biological specimen.

The study had two complementary purposes: first, to estimate the magnitude, direction, and uncertainty of the associations of Mediterranean Diet adherence, ultra-processed food consumption, physical activity, and sleep characteristics with gut microbiota diversity and composition; and second, to apply a reproducible analytical workflow and translate the information obtained from the pilot dataset into transparent, scenario-based sample-size planning benchmarks and design considerations for future studies. The dietary and lifestyle variables, the integrated analytical framework, and the future-study planning component were specific to the present investigation.

Given the pilot nature and limited sample size, the association analyses were considered exploratory and hypothesis-generating. Interpretation focused on effect estimates, confidence intervals, multiplicity-adjusted evidence, and estimate robustness rather than on statistical significance alone. The cross-sectional design did not permit assessment of temporal directionality or causal inference, and non-significant findings were not interpreted as evidence of the absence of an association.

The study is reported in accordance with the applicable items of the Strengthening the Reporting of Observational Studies in Epidemiology (STROBE) statement for cross-sectional studies and the Strengthening the Organization and Reporting of Microbiome Studies (STORMS) guidelines [16,20].

### 2.2. Source Cohort, Present Analytical Sample, and Ethics

Participants were recruited at the Greater Romagna Area Hub Laboratory in Pievesestina, Cesena, Italy, under the coordination of the Unit of Microbiology, Department of Laboratory and Transfusion Medicine, Local Health Authority of Romagna. Recruitment and stool collection were conducted between September and December 2025.

Eligibility was assessed using a study-specific 15-item screening questionnaire reproduced as Supplementary Questionnaire S1. Individuals aged 20–40 years were eligible if they reported no clinical, pharmacological, or behavioral conditions expected to substantially affect gut microbiota composition. Exclusion criteria comprised current or previous chronic systemic disease; regular medication use; antibiotic exposure during the preceding six months; use of probiotics, prebiotics, synbiotics, or other potentially microbiota-modulating supplements during the preceding four weeks; use of laxatives, antidiarrheal agents, proton-pump inhibitors, or bowel-cleansing preparations during the preceding four weeks; gastrointestinal infection during the preceding six months; autoimmune disease, inflammatory bowel disease, or irritable bowel syndrome; previous major gastrointestinal surgery; colonoscopy or bowel preparation during the preceding three months; acute gastrointestinal symptoms at screening; major dietary changes during the preceding four weeks; and pregnancy.

A total of 100 prospective participants were approached, provided written informed consent, and entered the recruitment process. Of these, 80 completed the eligibility questionnaire and underwent formal screening, whereas 20 did not complete screening. Among the 80 screened individuals, 62 fulfilled all eligibility criteria and 18 were excluded as ineligible. Eligible participants were stratified by sex, and 10 individuals were randomly selected without replacement from each stratum using the sample() function in R version 4.3.3 (R Foundation for Statistical Computing, Vienna, Austria). The resulting cohort comprised 20 healthy adults, including 10 females and 10 males. All selected participants provided a suitable stool specimen, and no replacements were required.

The analytical sample comprised the 20 participants prospectively selected from the 62 eligible individuals for stool collection and microbiome profiling. The dietary and lifestyle questionnaire formed part of the same prospective data-collection protocol: it was provided together with the eligibility-screening questionnaire, and participants selected for microbiome profiling were instructed to complete it on the day of stool production. The target of 20 participants reflected the feasibility and laboratory scope of the intensive source design, in which eight microbiome profiles were generated per participant, corresponding to 160 analytical profiles in total, while sex-stratified random selection ensured equal representation of females and males. The remaining 42 eligible individuals did not undergo stool collection or microbiome profiling and therefore could not contribute to the present analyses. All 20 selected participants had a prespecified fresh-stool microbiome profile and complete demographic, dietary, physical-activity, and sleep data and were included in the final analytical cohort. No participant was selected or excluded based on the observed exposure or microbiome values. No formal a priori power calculation was performed for the cross-sectional association analyses, which were consequently considered exploratory and hypothesis-generating. Microbiome and questionnaire-derived data were linked exclusively through coded study identifiers.

The study protocol was approved by the Comitato Etico della Romagna (CET-CEROM; study registration no. 3912; protocol no. 3372/2025; approval date: 1 August 2025), and the study was conducted in accordance with the Declaration of Helsinki. The approved protocol and written informed-consent process covered participant recruitment, stool collection, administration of the dietary and lifestyle questionnaire, and the integrated analysis of microbiome and questionnaire-derived data. Written informed consent was obtained before enrollment and before any study procedures were performed. No directly identifying personal information was available during laboratory processing or statistical analysis.

### 2.3. Stool Collection and Definition of the Analytical Microbiome Sample

Participants were instructed to collect a stool specimen after 8:00 p.m. on the evening preceding the scheduled delivery to the laboratory. Each specimen was collected in a sterile graduated polypropylene Coprotainer^®^ container (Sarstedt, Nümbrecht, Germany), refrigerated at 4 °C immediately after collection, transported to the laboratory under controlled refrigerated conditions at 4 °C, and processed within 24 h of defecation. Specimens exceeding the 24-h interval or not maintained under the required refrigerated conditions were considered unsuitable for analysis. All specimens included in the present study met the predefined collection, storage, transport, and processing requirements.

For each participant, only the microbiome profile generated from the fresh-stool aliquot that had not undergone homogenization or dilution was retained for the present study. No additional profile originating from the same participant or biological specimen was included. The final analytical microbiome dataset therefore comprised 20 independent profiles, one per participant.

### 2.4. DNA Extraction, 16S rRNA Gene Sequencing, and Bioinformatic Processing

Microbial DNA was extracted from 250 mg of fresh stool using the QIAamp PowerFecal Pro DNA Kit (cat. no. 51804; QIAGEN, Hilden, Germany) on a QIAcube Connect MDx automated platform (QIAGEN). Samples were transferred to PowerBead Pro tubes containing 800 µL of CD1 solution and subjected to vortexing, heat incubation, mechanical disruption using a TissueLyser II (QIAGEN), and centrifugation. Following the inhibitor-removal steps, 600 µL of clarified supernatant was processed through the automated silica-membrane purification workflow according to the manufacturer’s instructions. DNA concentration was measured fluorometrically using the Qubit 1X dsDNA High Sensitivity Assay Kit and a Qubit fluorometer (Invitrogen, Thermo Fisher Scientific, Waltham, MA, USA). No quantitative PCR, spike-in standard, or other method for absolute microbial-load quantification was employed; consequently, taxonomic measurements were interpreted as relative abundances.

DNA extracts with concentrations >1 ng/µL were diluted in UltraPure distilled water (Invitrogen, Thermo Fisher Scientific) to a target concentration of 1 ng/µL, whereas extracts with concentrations ≤ 1 ng/µL were processed without further dilution. Libraries were prepared using a Microlab VANTAGE automated liquid-handling platform (Hamilton Company, Reno, NV, USA) and the Microbiota Solution B Kit (cat. no. AD-002.024; Arrow Diagnostics, Genoa, Italy). The manufacturer-supplied primer mixture targeted the V3–V4 and V6 hypervariable regions of the bacterial 16S rRNA gene. Library preparation comprised a target PCR followed by an indexing PCR, with AMPure XP bead-based purification (Beckman Coulter, Brea, CA, USA) between the two amplification steps. Purified libraries were quantified fluorometrically, converted to molar concentrations assuming a nominal amplicon length of 900 bp, normalized to 10 nM, and pooled in equal volumes. Sequencing was performed on a MiSeq platform (Illumina, San Diego, CA, USA) using the MiSeq Reagent Kit v3 (Illumina) with 2 × 300-bp paired-end reads and sequencing-by-synthesis chemistry. PhiX Control v3 (Illumina) was added at 10% as an internal sequencing control. Reaction-mix components and thermal-cycling conditions are reported in Supplementary Table S1.

Raw sequencing data were processed using MicrobAT software version 1.3.0 (Microbiota Analysis Tool; SmartSeq S.r.l., Novara, Italy). The embedded workflow performed read-quality filtering, chimera handling, operational taxonomic unit assignment, taxonomic classification, and generation of abundance tables at multiple taxonomic levels. The clustering threshold, read-filtering cutoffs, chimera-handling parameters, classifier settings, and reference-database version were embedded within the software and were neither user-configurable nor reported in the available exported output. No separate external denoising or amplicon sequence variant inference was performed; the resulting feature table was therefore treated as an operational taxonomic unit–based dataset.

The exported OTU count table contained 1756 rows, including one software-generated aggregate row labelled “taxa1” that did not represent an individual OTU and was not included by MicrobAT in its reported observed richness. This aggregate row was therefore excluded from the alpha-diversity calculations. The remaining 1755 OTU rows were used to independently recalculate the alpha-diversity indices. For participant i, the relative abundance of OTU j was defined as p_ij_ = n_ij_/Σ_j_n_ij_, where n_ij_ denotes the corresponding read count. Shannon diversity was calculated as H_i_ = −Σ_j_p_ij_ ln(p_ij_), using natural logarithms and including OTUs with positive counts. Pielou’s evenness was calculated as J_i_ = H_i_/ln(S_i_), where S_i_ denotes the number of OTUs with positive counts in participant i. The independently recalculated values reproduced the MicrobAT-reported observed richness exactly and reproduced Shannon diversity and Pielou’s evenness within the two-decimal precision of the exported software output. Full-precision independently recalculated values were used in all subsequent alpha-diversity analyses. No rarefaction, prevalence filtering, or additional low-abundance filtering was applied in the primary alpha-diversity analysis.

For the downstream community-composition and taxon-specific analyses, the exported counts were aggregated at the genus level. The resulting table contained 241 genus-level features, including formally assigned genera and incompletely classified groups retained at the most informative available taxonomic level. Relative abundances were calculated within each participant by dividing each genus-level count by the total count in the corresponding genus-level table. These genus-level compositions were used to calculate Bray–Curtis dissimilarities, Aitchison distances, centered log-ratio coordinates, and the natural-log-transformed *Prevotella*/*Bacteroides* ratio.

Extracted DNA concentrations < 1 ng/µL and sequencing-library concentrations < 5 ng/µL were recorded as quality-control flags rather than automatic exclusion criteria. Extraction failure was defined as the absence of a recoverable DNA extract or failure to generate a sequencing library, whereas sequencing failure was defined as the absence of a usable MicrobAT-derived OTU profile. An analytical profile was retained when DNA extraction and library preparation were successful, sequencing yielded nonzero reads, and MicrobAT generated an OTU profile with valid taxonomic output, irrespective of DNA or library concentration alone. Blank controls and negative extraction controls were included in each sequencing run and evaluated as part of the routine laboratory quality-assurance workflow; mock-community controls had also been assessed during preliminary workflow verification. Review of the blank and negative extraction controls did not identify evidence of contamination requiring rejection of a sequencing run, repetition or exclusion of a participant sample, or removal of individual taxa. The controls were therefore not incorporated into the participant-level analytical dataset, and no control-based contaminant-removal procedure was required. No extraction, library-preparation, or sequencing failure was recorded among the 160 participant-derived profiles generated in the source laboratory study. All 20 fresh-stool profiles selected for the present analysis satisfied the prespecified analytical criteria and were retained.

Accordingly, the upstream primary bioinformatic processing performed within MicrobAT could not be reproduced independently in full because the proprietary clustering, read-filtering, chimera-handling, classification, and reference-database parameters were neither user-configurable nor included in the exported analytical output. By contrast, all downstream processing and statistical analyses starting from the exported OTU count table were explicitly specified and independently implemented. These procedures included exclusion of the software-generated aggregate row, recalculation of observed richness, Shannon diversity, and Pielou’s evenness, aggregation at the genus level, calculation of relative abundances and compositional transformations, derivation of microbiome outcomes, regression and permutation-based analyses, diagnostic and sensitivity procedures, and scenario-based sample-size calculations. The corresponding R scripts are available through the Zenodo repository identified in the Data Availability Statement.

### 2.5. Assessment of Dietary and Lifestyle Exposures

Dietary and lifestyle information was collected using a structured questionnaire completed under a coded participant identifier. Participants were instructed to report their usual dietary behaviors, whereas the physical-activity and sleep sections retained their respective reference periods of the preceding seven days and preceding month. Four exposures were examined: adherence to the Mediterranean Diet, consumption of ultra-processed foods, physical activity, and sleep characteristics.

Mediterranean Diet adherence. Adherence to the Mediterranean Diet was assessed using the validated 14-item Mediterranean Diet Adherence Screener (MEDAS), developed within the PREDIMED framework [21]. Each item was assigned 1 point when the corresponding adherence criterion was met and 0 points otherwise. Item scores were summed to obtain a total score ranging from 0 to 14, with higher values indicating greater adherence. For descriptive purposes, scores were classified as indicating low (0–5), intermediate (6–9), or high (10–14) adherence.

Ultra-processed food consumption. Habitual ultra-processed food consumption was assessed using a study-specific nine-item frequency score developed before the present analyses and informed by the NOVA food-classification framework [22]. The nine categories—packaged savory snacks, sugar-sweetened beverages, industrially produced sweets, fast food, ready-to-eat or frozen meals, processed meats, sweetened breakfast cereals, industrially produced sauces, and energy or functional beverages—were selected a priori to represent commonly encountered, readily recognizable groups of industrially formulated foods and beverages that could be reported without requiring product-level coding. The instrument was intended as a brief indicator of consumption frequency and not as an exhaustive assessment of all foods potentially classified within NOVA group 4. For each category, reported frequency was scored as 0 for never or almost never, 1 for once per week, 2 for two to four times per week, and 3 for five or more times per week. These pragmatic ordinal cut-offs were chosen to distinguish absent or negligible, occasional, recurrent, and frequent consumption while maintaining a response format suitable for a brief questionnaire. Items were equally weighted because no validated empirical basis was available for assigning differential weights to the nine categories; consequently, the score assumes that each category and each one-point frequency increment contribute equally to the total. Item scores were summed to obtain a total ranging from 0 to 27, with higher values indicating more frequent reported consumption. For descriptive presentation only, the theoretical score range was divided into three equal-width operational intervals: low (0–9), intermediate (10–18), and high (19–27). These categories were not used as the primary inferential exposures, for which the continuous score was retained. The score has not undergone independent validation and does not quantify portion size, consumed weight, total energy intake, or the proportion of dietary energy derived from ultra-processed foods. It was therefore treated as a structured exploratory frequency measure rather than as a validated quantitative estimate of ultra-processed food intake.

Physical activity. Physical activity during the preceding seven days was assessed using the International Physical Activity Questionnaire–Short Form (IPAQ-SF) [23]. Participants reported the number of days per week and minutes per day spent walking and performing moderate- and vigorous-intensity activities in bouts lasting at least 10 min. Sitting time during a typical weekday was also recorded as a descriptive indicator of sedentary behavior but was not included in the total physical-activity score. In accordance with the IPAQ-SF data-processing protocol, reported durations exceeding 180 min/day within any activity-intensity category were truncated to 180 min/day before score calculation. Total physical activity was expressed as metabolic equivalent task minutes per week (MET-min/week) and calculated as follows:

Total MET−min/week=3.3(Wm×Wd)+4.0(Mm×Md)+8.0(Vm×Vd)

where Wm, Mm, and Vm denote the minutes per day spent walking and performing moderate- and vigorous-intensity activities, respectively, and Wd, Md, and Vd denote the corresponding numbers of days per week. Participants were descriptively classified as having low, moderate, or high physical-activity levels according to the standard IPAQ-SF scoring criteria. The score calculated after applying the 180-min/day truncation rule was used in the primary analyses; scores derived from the original untruncated responses were examined in a sensitivity analysis.

Sleep characteristics. Sleep characteristics during the month preceding questionnaire completion were assessed using a study-specific instrument adapted from the principal domains of the Pittsburgh Sleep Quality Index (PSQI) [24]. The instrument evaluated seven components: subjective sleep quality, sleep latency, sleep duration, habitual sleep efficiency, sleep disturbances, use of sleep medication, and daytime dysfunction. Participants reported their habitual bedtime, wake time, sleep latency, and mean duration of actual sleep per night. Reported sleep duration was used to score the sleep-duration component. Time spent in bed was calculated from the reported bedtime and wake time, accounting for overnight intervals, and habitual sleep efficiency was calculated as the ratio of reported sleep duration to time spent in bed, multiplied by 100. Each component was scored from 0 to 3, with higher values indicating greater reported sleep difficulty, and the seven component scores were summed to obtain an adapted sleep score ranging from 0 to 21. Component derivation and the corresponding scoring rules are reported in Supplementary Table S2, Panel B. For descriptive summaries only, the total score was assigned to four prespecified operational bands: Band 1 (0–4), Band 2 (5–9), Band 3 (10–14), and Band 4 (15–21). These cutoffs partitioned the theoretical score range into successive five-point intervals, with the upper band including the remaining values up to the theoretical maximum; they were pragmatic descriptive thresholds and were not derived from clinical validation. The continuous adapted sleep score was used in all association analyses, whereas the operational bands were used only to summarize the sample and assess multivariate dispersion. Because the instrument was an adaptation and did not constitute administration of the complete validated PSQI, neither the total score nor the operational bands were interpreted as validated measures of sleep quality, clinically meaningful categories, or diagnostic classifications.

MEDAS, UPF, IPAQ-SF MET-min/week, and adapted sleep scores were analyzed as continuous exposures. MEDAS and IPAQ-SF categories were applied according to their respective scoring frameworks, whereas the UPF categories and adapted sleep-score ranges were study-specific operational groupings. All categories and score ranges were used exclusively for descriptive summaries and multivariate-dispersion diagnostics and were not included as primary inferential exposures. Complete information required to derive all four continuous exposure scores was available for the 20 participants. The data-collection instrument is reproduced as Supplementary Questionnaire S2, and the complete coding rules, component definitions, scoring thresholds, formulas, and descriptive groupings are reported in Supplementary Table S2.

### 2.6. Covariates

Potential confounding was addressed at both the design and analysis stages. At the design stage, the eligibility criteria restricted the cohort to healthy adults and excluded major conditions and exposures known to affect the gut microbiota, including chronic systemic or gastrointestinal disease, regular medication use, recent antibiotic exposure, recent use of microbiota-modulating supplements, gastrointestinal infection, acute gastrointestinal symptoms, and major recent dietary changes. These characteristics therefore functioned as restriction criteria rather than model covariates. Within the resulting defined cohort, age and sex were obtained from the demographic section of the structured questionnaire and selected a priori for covariate adjustment because of their potential relationships with both dietary and lifestyle exposures and gut microbiome characteristics. Age was analyzed as a continuous variable expressed in years. Sex was included as a binary categorical variable, with female as the reference category and male as the comparison category. Adiposity or body composition, stool consistency, bowel frequency or intestinal transit, and total energy intake were not measured in sufficient detail for inclusion as covariates. Given the analytical sample of 20 participants, adjustment was intentionally restricted to age and sex to preserve model parsimony and limit overfitting. No data-driven covariate-selection procedure was applied, and complete age and sex information was available for all participants.

### 2.7. Microbiome Outcomes

For the dietary and lifestyle analyses conducted in the present study, microbiome measures were organized into three complementary domains: within-sample diversity, between-sample community composition, and taxon-specific variation.

Within-sample alpha diversity was quantified at the OTU level using the full-precision values independently recalculated from the exported OTU count table. The Shannon diversity index was designated as the primary microbiome outcome for the association analyses because it accounts for both the number of OTUs detected within each sample and the distribution of their relative abundances. Pielou’s evenness was examined as a complementary alpha-diversity outcome and was defined as Shannon diversity normalized by the natural logarithm of observed OTU richness. It therefore describes the uniformity of the abundance distribution among the OTUs detected within each participant.

Between-sample differences in overall microbial community composition were evaluated as secondary outcomes using two complementary measures. Bray–Curtis dissimilarity was calculated from genus-level relative abundances to quantify differences in community structure while giving greater weight to more abundant genera. Aitchison distance was calculated as the Euclidean distance between centered log-ratio-transformed genus-level compositions and was used to account for the compositional nature of microbiome abundance data. The complete genus-level abundance matrix was used in the primary beta-diversity analyses. Robustness was evaluated using prevalence-filtered matrices and alternative pseudocount values.

Exploratory taxon-specific analyses were restricted to eight bacterial genera selected a priori, before inspection of the genus-level abundance and prevalence distributions in the current dataset and before any exposure–taxon association modelling: *Bacteroides*, *Prevotella*, *Faecalibacterium*, *Bifidobacterium*, *Blautia*, *Roseburia*, *Ruminococcus*, and *Akkermansia*. The selection was based on published human evidence rather than on patterns observed in the present sample. *Bacteroides* and *Prevotella* were included to represent the well-described diet-associated compositional gradient linked to long-term dietary patterns [25]. *Faecalibacterium*, *Roseburia*, *Ruminococcus*, and *Blautia* were included because human observational and dietary-intervention studies have repeatedly implicated these genera in diet–microbiome relationships and fermentation-related microbial functions [26]. Bifidobacterium was included because of its documented responsiveness to fermentable plant substrates in human dietary-intervention studies [27], whereas *Akkermansia* was included because of its mucin-associated ecology and documented relevance to human metabolic health [28]. Selection was not based on the abundance or prevalence observed in the current dataset, the direction or magnitude of any exposure coefficient, or nominal statistical significance. To preserve the relative information inherent to compositional sequencing data, taxon-specific primary analyses used centered log-ratio coordinates calculated from the complete genus-level composition after addition of the prespecified pseudocount. Alternative pseudocounts and models based on arcsine-square-root-transformed, natural-log-transformed, and untransformed relative abundances were examined exclusively in sensitivity analyses.

The relative balance between *Prevotella* and *Bacteroides* was additionally evaluated as a continuous natural-log-transformed ratio. This measure represented the relative predominance of the two genera along a compositional gradient and was not interpreted as evidence of discrete enterotypes.

Given the pilot sample size and the number of taxon-specific comparisons, analyses of the selected genera and the *Prevotella*/*Bacteroides* log-ratio were considered exploratory. Interpretation therefore focused on the magnitude, direction, uncertainty, multiplicity-adjusted evidence, and robustness of the estimated associations rather than on nominal statistical significance alone.

### 2.8. Statistical Analysis

Statistical analyses were designed to estimate the magnitude, direction, and uncertainty of the associations observed in this pilot study. Complete microbiome outcome, dietary and lifestyle exposure, age, and sex data were available for all 20 participants. Complete-case analyses were therefore performed, and no missing-data imputation was required. Continuous variables were summarized using the mean and standard deviation and, where informative, the median and interquartile range. Categorical variables were reported as counts and percentages.

Mediterranean Diet adherence, ultra-processed food consumption, physical activity, and sleep characteristics were analyzed as continuous scores in their respective measurement units. Each exposure was examined in a separate model to limit model complexity and reduce the risk of overfitting. All association models were adjusted for age, expressed in years, and sex, with female as the reference category. The general model specification was:

$$Y_{i}\;=\;\beta_{0}\;+\;\beta_{1}X_{i}\;+\;\beta_{2}{Age}_{i}\;+\;\beta_{3}{Sex}_{i}\;+\;\varepsilon_{i}$$

where $Y_{i}$ denotes the microbiome outcome and $X_{i}$ the dietary or lifestyle exposure of interest for participant i. No interaction terms or data-driven variable-selection procedures were applied.

The IPAQ-SF score obtained after truncating reported activity durations exceeding 180 min/day was used in the primary analyses. To assess the influence of this data-processing rule, analyses involving Shannon diversity, Pielou’s evenness, the centered log-ratio coordinates of the eight selected genera, the *Prevotella*/*Bacteroides* log-ratio, and the two community-composition measures were repeated using the IPAQ-SF score derived from the original untruncated responses. Covariates and all other model specifications were kept unchanged.

Associations with Shannon diversity and Pielou’s evenness were evaluated using ordinary least-squares linear regression. For each exposure–outcome combination, the exposure coefficient was reported as the unstandardized mean difference in the outcome associated with a one-unit increase in the exposure score, together with its standard error, 95% confidence interval, and two-sided *p*-value. The exposure-specific partial *R*^2^ was calculated as:

$$Partial\;R^{2}=\frac{t^{2}}{t^{2}+\;dfresidual}$$

where t denotes the test statistic for the exposure coefficient and dfresidual = 16 in the complete models adjusted for age and sex.

Additional diagnostic and sensitivity analyses were conducted to evaluate the potential dependence of the alpha-diversity results on sequencing depth. The participant-specific number of classified OTU reads contributing to the independently recalculated alpha-diversity values was used as the sequencing-depth measure. Pearson correlations were calculated between sequencing depth and each alpha-diversity index using both the original and log10-transformed depth scales, and Spearman rank correlations were used to evaluate monotonic associations. Individual Shannon rarefaction curves were generated by repeated subsampling without replacement, using 100 independent subsamples at each evaluated depth and a random seed of 321. For the common-depth sensitivity analysis, the OTU count table of each participant was rarefied 1000 times without replacement to the minimum depth observed in the analytical dataset, corresponding to 3026 classified OTU reads, using the rrarefy function in the vegan package and a random seed of 123. Participant-specific mean Shannon and Pielou values across the 1000 repetitions were calculated, and their concordance with the corresponding full-depth values was assessed using Pearson and Spearman correlations. The eight age- and sex-adjusted exposure–alpha-diversity models were then repeated using the mean rarefied values. The same eight models were also repeated after excluding participant 7F, who had the lowest classified OTU-read depth. These evaluations were used exclusively as sequencing-depth diagnostics and sensitivity analyses and did not replace the full-depth primary alpha-diversity analysis.

The primary taxon-specific analyses were conducted using centered log-ratio coordinates derived from the complete genus-level composition, comprising 241 features. For sample i and feature j, the centered log-ratio coordinate was calculated as:

$$clr(x_{ij})\;=\;ln(x_{ij}\;+\;c)\;-\;(1/D)\sum_{k=1}^{D}ln(x_{ik}\;+\;c)$$

where $x_{\mathrm{ij}}$ denotes the relative abundance of feature j, D is the total number of genus-level features, and c = 1 × 10^−6^ in relative-abundance units. Separate age- and sex-adjusted linear models were fitted for each combination of the four exposures and eight selected genera. For graphical presentation in the main manuscript, exposure coefficients and their confidence limits were standardized by multiplying each unstandardized value by the ratio of the standard deviation of the corresponding exposure to the standard deviation of the genus-specific CLR outcome. For supplementary visualization, each partial *R*^2^ value was assigned the sign of its corresponding exposure coefficient; this signed quantity was used exclusively for color encoding and was not treated as a separate inferential effect-size measure. Sensitivity analyses were conducted using: (i) arcsine-square-root-transformed proportions, arcsin(√x); (ii) natural-log-transformed abundances, $ln(x+10^{-6})$; (iii) untransformed relative abundances; and (iv) centered log-ratio coordinates calculated using alternative pseudocounts of 1 × 10^−8^ and 1 × 10^−4^. These alternative representations were evaluated to determine whether the direction and interpretation of the associations depended materially on the scale used to represent taxonomic abundance [29].

The relative balance between *Prevotella* and *Bacteroides* was calculated as:

$$\ln\left[\frac{Prevotella+cPB}{Bacteroides+cPB}\right]$$

where cPB = 8.262619 × 10^−6^, corresponding to one-half of the smallest non-zero relative abundance observed for either genus in the analytical dataset. The log-ratio was examined as a continuous outcome using separate age- and sex-adjusted linear models for each exposure. To assess whether the microbiome profile with the lowest sequencing depth materially influenced the log-ratio results, all four models were additionally refitted after temporarily excluding participant 7F. The four resulting *p*-values were adjusted as a separate sensitivity family using the Benjamini–Hochberg procedure.

Between-sample community composition was evaluated using Bray–Curtis dissimilarities calculated directly from the complete genus-level relative-abundance matrix and Aitchison distances calculated as Euclidean distances between centered log-ratio-transformed compositions. In the primary Aitchison analysis, a pseudocount of 1 × 10^−6^ was added to each relative abundance before centered log-ratio transformation.

The association between each continuous exposure and community composition was evaluated using permutational multivariate analysis of variance (PERMANOVA) [30]. Separate models were fitted for each combination of the four exposures and two distance measures according to the following specification:

D ∼ X + Age + Sex

where D denotes the relevant distance matrix and X the exposure under investigation. The marginal effect of the exposure, conditional on age and sex, was tested using the adonis2 function with the by = “margin” option. Results were reported as marginal pseudo-F statistics, marginal *R*^2^ values, and permutation-derived *p*-values. Marginal *R*^2^ represented the proportion of total community variation attributable to the exposure after accounting for the other variables in the model and was not interpreted as partial *R*^2^ in the conventional regression sense.

All permutation-based analyses used the same matrix of 9999 unrestricted permutations, generated after setting the random seed to 123. Permutation-derived *p*-values were calculated using the observed pseudo-F statistic and the corresponding upper tail of the permutation distribution. Principal coordinate analysis was used exclusively to visualize between-sample dissimilarities and not for formal hypothesis testing.

Because PERMANOVA may be influenced by differences in both community location and within-group dispersion, homogeneity of multivariate dispersions was examined diagnostically [31]. For this purpose only, participants were grouped according to the operational categories defined for the corresponding dietary or lifestyle instrument. Distances to group spatial medians were calculated using betadisper with type = “median” and small-sample bias adjustment. Differences in dispersion were evaluated using permutest with the same set of 9999 permutations. These categorical analyses were used exclusively for diagnostic assessment and did not replace the primary PERMANOVA models based on continuous exposure scores.

Beta-diversity sensitivity analyses included repeating the Bray–Curtis and Aitchison analyses after retaining only genus-level features with a relative abundance greater than zero in at least four participants, corresponding to a prevalence threshold of 20%. This filtering retained 161 of the original 241 features. After filtering, relative abundances were reclosed to sum to one within each sample. The sensitivity of Aitchison results to zero handling was additionally evaluated by repeating the analysis using pseudocounts of 1 × 10^−8^ and 1 × 10^−4^.

Linear-model assumptions and the influence of individual observations were evaluated using residual-versus-fitted plots, quantile–quantile plots, the Shapiro–Wilk test applied to model residuals, the Breusch–Pagan test for heteroscedasticity, variance inflation factors, and Cook’s distance. A variance inflation factor greater than 5 was considered indicative of potentially problematic collinearity. Observations with Cook’s distance greater than 4/n = 0.20 were examined using leave-one-out analyses in which each participant was excluded in turn. No observation was excluded solely on the basis of diagnostic measures.

Conventional ordinary least-squares inference was considered the primary inferential approach for univariate outcomes. Its robustness was evaluated using two sensitivity approaches. First, heteroscedasticity-consistent HC3 standard errors, 95% confidence intervals, and two-sided *p*-values were calculated using the small-sample correction proposed by MacKinnon and White [32]. HC3 confidence intervals and tests were calculated using the t distribution and the residual degrees of freedom of the model. Second, age- and sex-adjusted Freedman–Lane permutation tests based on 9999 permutations were performed [33]. Residuals from the reduced model containing age and sex were permuted, added to the fitted values from the reduced model, and analyzed using the complete model containing the exposure, age, and sex. Two-sided permutation *p*-values were calculated from the absolute exposure t statistic using the plus-one correction:

$$pperm=[1+\sum_{\beta =1}^{B}I(|t_{\beta }|\geq |tobs|)]/(B+1)$$

where B = 9999, tobs denotes the observed exposure statistic, and ***t*_β_** the corresponding statistic obtained in permutation b. Diagnostic tests were not used to select the inferential method producing the smallest *p*-value. Conventional, HC3, and permutation-based results were instead compared to evaluate the stability of the estimates and conclusions.

The Benjamini–Hochberg procedure was used to control the false discovery rate (FDR) [34]. In the primary analyses, adjustment was applied separately within four families defined according to microbiome domain: alpha diversity, comprising eight comparisons (four exposures × two indices); beta diversity, comprising eight comparisons (four exposures × two distance measures); selected genera, comprising 32 comparisons (four exposures × eight genera); and the *Prevotella*/*Bacteroides* log-ratio, comprising four comparisons. The eight multivariate-dispersion tests were adjusted as a separate diagnostic family. For univariate outcomes, the Benjamini–Hochberg procedure was applied separately within each family to conventional, HC3, and Freedman–Lane *p*-values.

For the alpha-diversity sequencing-depth sensitivity analyses, Benjamini–Hochberg adjustment was applied separately across the eight models based on the mean rarefied values and across the eight models fitted after exclusion of the lowest-depth participant. For taxonomic sensitivity analyses, multiplicity adjustment was applied separately for each alternative transformation or pseudocount. Similarly, beta-diversity sensitivity analyses based on prevalence filtering, alternative pseudocounts, and the untruncated IPAQ-SF score were adjusted separately from the primary analyses. Univariate analyses based on the untruncated IPAQ-SF score were adjusted separately within the corresponding microbiome domain. Sensitivity analyses were therefore not combined with their respective primary families.

Both nominal and FDR-adjusted *p*-values were retained. In the primary analyses, an FDR-adjusted *p*-value below 0.05 was considered evidence against the corresponding null hypothesis. Interpretation nevertheless prioritized effect sizes, confidence intervals, consistency across sensitivity analyses, and the exploratory nature of the study rather than statistical significance alone. Tests of regression coefficients were two-sided, whereas PERMANOVA and multivariate-dispersion tests used the upper tail of their respective permutation distributions.

All statistical analyses were performed using R version 4.5.1 (R Foundation for Statistical Computing, Vienna, Austria). The principal R packages were vegan version 2.7-2 for ecological distances, rarefaction, PERMANOVA, and multivariate-dispersion analyses; permute version 0.9-10 for generation of the common permutation matrix; sandwich version 3.1-1 for HC3 covariance estimation; car version 3.1-3 for regression-model diagnostics; and readxl version 1.4.5 for questionnaire-data import. The complete analysis script, random seeds, permutation specifications, package versions, and session information were retained to support computational reproducibility.

### 2.9. Scenario-Based Sample-Size Planning for Future Regression Analyses

Scenario-based sample-size calculations were conducted to provide transparent planning benchmarks for future studies using age- and sex-adjusted cross-sectional linear regression models. These calculations were intended exclusively for prospective study design and were not used to estimate the achieved power of the present study, reinterpret its nonsignificant findings, or provide formal power estimates for longitudinal or distance-based community-composition analyses.

For continuous microbiome outcomes analysed using multiple linear regression, the exposure-specific effect was represented by the partial coefficient of determination, $R_{partial}^{2}$, and converted to Cohen’s $\left(f^{2}\right)$ [35]:

$$f^{2}=\frac{R_{partial}^{2}}{1-R_{partial}^{2}}$$

The scenario grid considered partial *R*^2^ values of 0.01, 0.03, 0.05, and 0.10 to represent a broad range of exposure-specific effects. Partial *R*^2^ values of 0.03 and 0.05 correspond to Cohen’s *f*^2^ values of approximately 0.031 and 0.053, respectively, and were designated as the principal modest-effect planning range. This range was selected because effects of this magnitude may remain scientifically relevant in a multifactorial microbiome setting while avoiding reliance on the highly unstable, exposure-specific estimates obtained from the small pilot sample. The 0.01 and 0.10 scenarios were retained to show how the required sample size would change under smaller or larger assumed effects. The required number of analyzable participants was estimated for a fixed-effects multiple linear regression model testing one exposure coefficient while adjusting for age and sex. The model therefore included three predictors in addition to the intercept.

The noncentral (F) distribution had one numerator degree of freedom, (N-4) denominator degrees of freedom, and the following noncentrality parameter:

$$\lambda =f^{2}N$$

For each effect-size scenario, the minimum integer (*N*) required to achieve either 80% or 90% statistical power was identified. Calculations were performed using a nominal significance level of α=0.05 and, as a more stringent sensitivity scenario, a Bonferroni-adjusted threshold accounting for the four principal exposures:

$$\alpha_{conservative}=\frac{0.05}{4}=0.0125$$

The partial $\left(R^{2}\right)$ estimates obtained in the pilot analyses of Shannon diversity, Pielou evenness, the CLR-transformed abundances of the eight prespecified genera, and the continuous log-transformed *Prevotella*-to-*Bacteroides* ratio were additionally evaluated as exploratory planning inputs. Because effect-size estimates from small pilot samples can be unstable, these observed values were not used as the sole basis for defining a recommended sample size [36].

To account for attrition, incomplete exposure measurements, unsuccessful sequencing, or other sources of unusable observations, the required number of enrolled participants was calculated as:

$$N_{enrolled}=\left\lceil \frac{N_{analysable}}{1-a}\right\rceil$$

where a denotes the anticipated proportion of enrolled participants without analyzable data. Loss rates of 10%, 15%, and 20% were examined.

For longitudinal workload scenarios, the total number of planned stool specimens was calculated as:

$$N_{specimens}=m\times N_{enrolled}$$

where (m) denotes the number of planned stool collections per participant. Scenarios with three and four repeated collections were considered.

Because the dataset contained a single temporal observation per participant, within-participant temporal variance, temporal correlation, and changes in the exposures could not be estimated. Participant-level sample-size calculations were therefore based exclusively on cross-sectional linear regression models and were not adjusted for any potential efficiency gain associated with repeated measurements. Repeated collections affected only the estimated total number of stool specimens in the longitudinal workload scenarios. No formal longitudinal or PERMANOVA sample-size calculation was performed. A future study in which community composition constitutes the primary outcome should use a dedicated simulation-based power analysis incorporating the intended distance measure, multivariate covariance structure, exposure distribution, covariates, permutation strategy, and repeated-measures design.

## 3. Results

### 3.1. Participant Flow and Sample Characteristics

**Figure 1:**
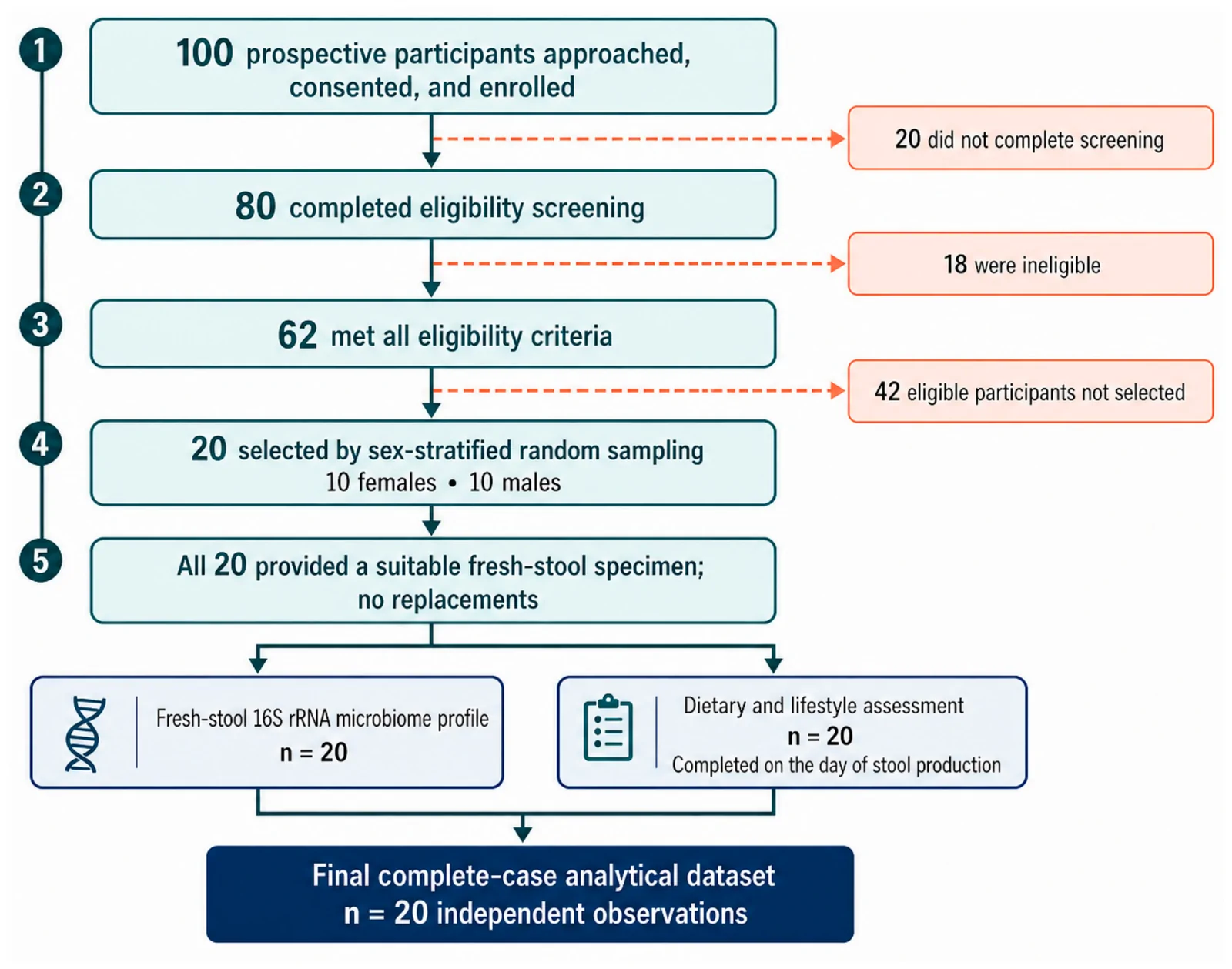
Participant recruitment and construction of the final analytical dataset. Of the 100 prospective participants who were approached, provided written informed consent, and were enrolled, 80 completed eligibility screening, whereas 20 did not complete the screening process. Among the 80 screened participants, 62 met all eligibility criteria and 18 were ineligible. Twenty participants were selected without replacement through sex-stratified computer-generated random sampling, comprising 10 females and 10 males; the remaining 42 eligible participants were not selected. All selected participants provided a suitable fresh-stool specimen and completed the dietary and lifestyle assessment on the same day that the specimen was produced for laboratory delivery, yielding a complete-case analytical dataset of 20 independent participant-level observations.

A total of 100 prospective participants were approached, provided written informed consent, and were enrolled. Of these, 80 completed the eligibility questionnaire and underwent formal screening, whereas 20 did not complete the screening process. Among the 80 screened participants, 62 met all eligibility criteria and 18 were ineligible. From the 62 eligible participants, 20 were selected without replacement through sex-stratified computer-generated random sampling, comprising 10 females and 10 males; the remaining 42 eligible participants were not selected. All 20 selected participants provided a suitable fresh-stool specimen and completed the dietary and lifestyle assessment on the same day that the specimen was produced for laboratory delivery, as instructed. No selected participant required replacement, and no microbiome or exposure data were missing. The final complete-case analytical dataset therefore comprised 20 independent participant-level observations (Figure 1).

The mean age of the analytical sample was 28.8 ± 4.5 years, with a median of 28.0 years (interquartile range [IQR], 26.0–31.3) and a range of 21–40 years. Participant age and sex are summarized in Table 1, and participant-level demographic characteristics are reported in Supplementary Table S3.

### 3.2. Distribution of Dietary and Lifestyle Exposures

The distributions of the four dietary and lifestyle exposures are summarized in Table 1. Participant-level demographic characteristics, total exposure scores, and descriptive classifications are reported in Supplementary Table S3.

The mean Mediterranean Diet Adherence Screener (MEDAS) score was 8.25 ± 1.83, with a median of 8.5 (interquartile range [IQR], 7.0–10.0) and a range of 4–11. Two participants (10.0%) were classified as having low adherence, 12 (60.0%) as having intermediate adherence, and six (30.0%) as having high adherence. The most frequently satisfied MEDAS criteria were consumption of fewer than one serving of red or processed meat per day (19/20, 95.0%), use of extra-virgin olive oil as the principal culinary fat (18/20, 90.0%), and consumption of fewer than one serving of butter, margarine, or cream per day (18/20, 90.0%). The least frequently satisfied criteria were consumption of at least seven glasses of wine per week (4/20, 20.0%), at least three weekly servings of legumes (5/20, 25.0%), and at least three weekly servings of fish or seafood (5/20, 25.0%). Participant-level responses to the 14 MEDAS items are reported in Supplementary Table S4A,B.

The mean study-specific ultra-processed food (UPF) score was 7.25 ± 3.73, with a median of 6.5 (IQR, 5.0–8.5) and a range of 2–18. Fifteen participants (75.0%) were classified in the low-consumption category and five (25.0%) in the intermediate-consumption category; none were classified in the high-consumption category. Energy or functional beverages were never or almost never consumed by 15 participants (75.0%), fast food by 12 (60.0%), and sugar-sweetened breakfast cereals by 11 (55.0%). The highest prevalence of at least weekly consumption was observed for packaged savory snacks and industrially produced sweets (15/20 for each category, 75.0%), followed by industrially produced sauces (13/20, 65.0%). These item-level results describe reported consumption frequency and should not be interpreted as estimates of the weight, energy contribution, or proportion of the diet represented by ultra-processed foods. Participant-level responses to the nine UPF items are reported in Supplementary Table S5.

After application of the International Physical Activity Questionnaire–Short Form (IPAQ-SF) scoring rule that truncates activity durations exceeding 180 min/day, mean physical activity was 2524.0 ± 2007.7 metabolic equivalent of task minutes per week (MET-min/week), with a median of 1902.5 MET-min/week (IQR, 1171.3–4255.5) and a range of 0–6444 MET-min/week. Nine participants (45.0%) were classified as having high, six (30.0%) moderate, and five (25.0%) low physical-activity levels. One participant reported 240 min/day of walking; truncation to 180 min/day reduced that participant’s total score from 6984 to 5598 MET-min/week without changing the High classification. Median reported sitting time on a typical weekday was 120 min/day (IQR, 120–240; range, 90–480). Participant-level IPAQ-SF responses, corrected MET-min/week values, and physical-activity categories are reported in Supplementary Table S6.

The mean adapted sleep score was 5.15 ± 3.34, with a median of 5.0 (IQR, 2.8–7.0) and a range of 0–13. Seven participants (35.0%) had scores in the range of 0–4, 11 (55.0%) in the range of 5–9, and two (10.0%) in the range of 10–14; no participant had a score in the range of 15–21. These score ranges were used exclusively to describe the distribution of the adapted score and did not represent validated or clinically meaningful sleep categories. Mean reported sleep duration was 7.08 ± 1.52 h/night, with a median of 7.0 h (IQR, 6.88–8.00) and a range of 3.5–9.0 h. Median sleep latency was 10 min (IQR, 5–20; range, 5–60). On the subjective sleep-quality item, 15 participants (75.0%) rated their sleep as very good or fairly good, whereas five (25.0%) rated it as fairly poor or very poor. Participant-level sleep timing, duration, subjective quality, disturbance-related responses, sleep-medication use, and daytime consequences are reported in Supplementary Table S7A–C. The seven adapted component scores, total adapted sleep scores, and descriptive score ranges are provided in Supplementary Table S8.

The continuous MEDAS, UPF, IPAQ-SF, and adapted sleep scores were used as exposure variables in the association analyses. MEDAS and IPAQ-SF categories, the study-specific UPF categories, and the adapted sleep-score ranges were used exclusively for descriptive characterization and multivariate-dispersion diagnostics.

### 3.3. Sequencing Performance and Descriptive Gut Microbiota Profile

All 20 fresh-stool samples yielded valid sequencing-derived taxonomic profiles and were retained in the analytical dataset. The mean number of total reads per sample was 186,570.8 ± 48,767.9, of which 168,200.4 ± 46,187.5 were retained after quality filtering. The mean proportion of retained reads was 88.6 ± 7.5%. A mean of 144,753.9 ± 39,989.3 reads per sample were taxonomically classified using the Ribosomal Database Project (RDP) classifier, corresponding to 85.8 ± 3.9% of quality-filtered reads. Detailed sequencing and quality-control metrics are reported in Supplementary Table S9, and participant-level total, quality-filtered, and RDP-classified read counts are shown in Supplementary Figure S1.

Total sequencing depth ranged from 24,738 to 256,997 reads per sample. Participant 7F had the lowest overall sequencing depth, with 24,738 total reads, 14,311 quality-filtered reads, and 11,595 RDP-classified reads. Within the exported OTU count table used for the independent alpha-diversity recalculation, classified OTU-read totals had a mean of 114,475.4 ± 36,408.1 reads per participant and ranged from 3026 to 161,259 reads. Participant 7F also had the lowest classified OTU-read depth, with 3026 reads. RDP-classified read counts were obtained from the sequencing-statistics output, whereas classified OTU-read totals were calculated from the non-aggregate rows of the exported OTU count table; these measures derive from different pipeline outputs and are therefore not directly interchangeable. Because participant 7F nevertheless yielded a valid taxonomic profile, the sample was retained in the primary analyses. Its potential influence was evaluated using sequencing-depth correlations, repeated rarefaction, and exclusion sensitivity analyses for alpha diversity, as well as the prespecified exclusion sensitivity analysis for the natural-log-transformed *Prevotella*/*Bacteroides* ratio.

After preprocessing, the final genus-level abundance table contained 241 taxa or taxonomic groups, comprising both classified genera and incompletely classified groups reported at the most informative taxonomic level available. The ten most abundant genus-level taxonomic groups accounted for a mean cumulative relative abundance of 59.89%. *Bacteroides* had the highest mean relative abundance (15.34%), followed by unclassified *Lachnospiraceae* (11.27%) and *Faecalibacterium* (7.69%). The remaining dominant groups were unclassified Clostridiales, unclassified Ruminococcaceae, unclassified Bacteroidales, *Bifidobacterium*, *Blautia*, *Prevotella*, and *Lachnospiraceae incertae sedis*.

**Figure 2:**
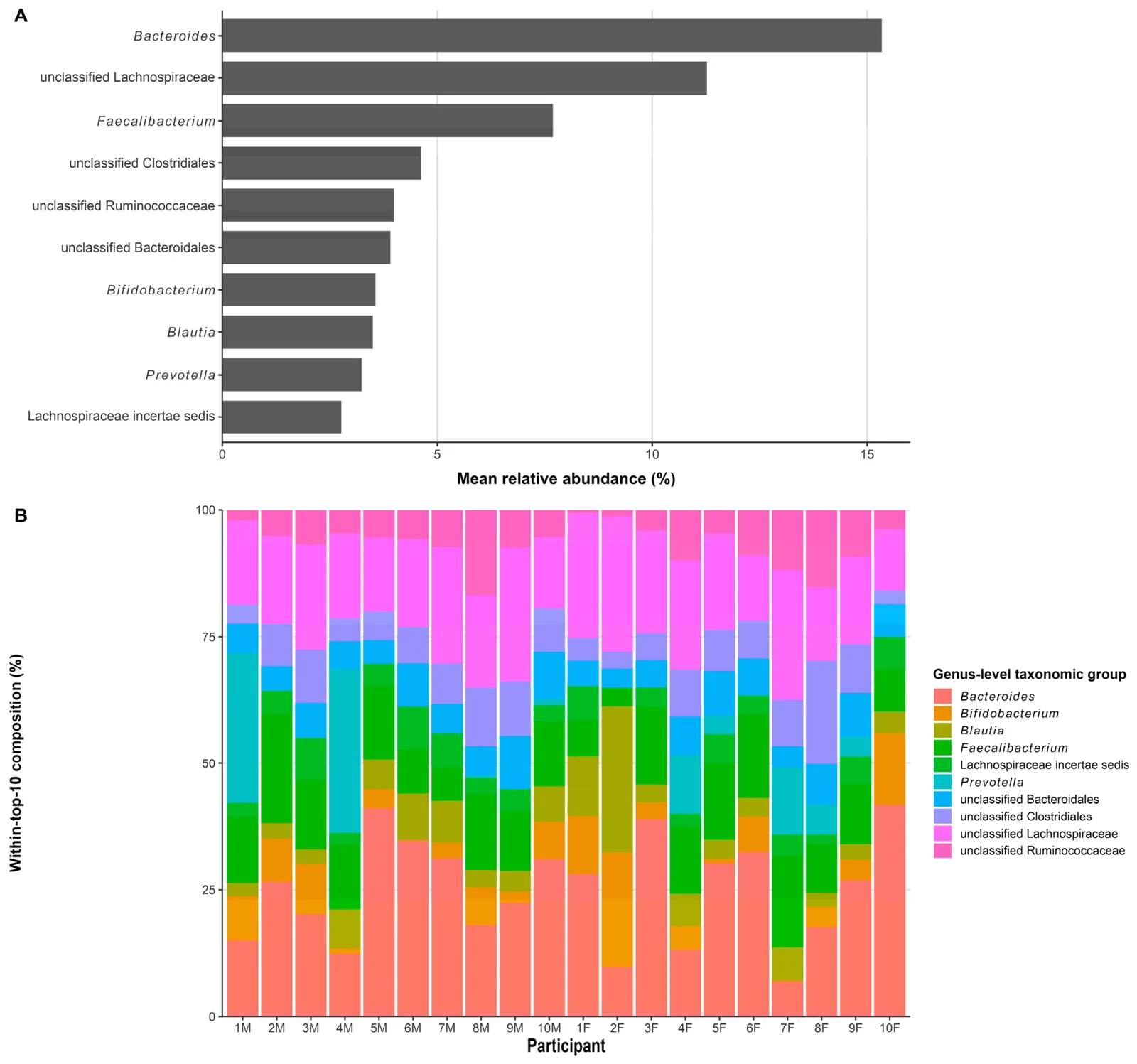
Dominant genus-level taxonomic groups in the 20 fresh-stool microbiome profiles. (**A**) Mean relative abundance of the ten most abundant genus-level taxonomic groups. (**B**) Participant-level composition of the same ten taxonomic groups after their summed relative abundance was rescaled to 100% within each participant. The ten groups were selected according to their mean participant-level relative abundance and collectively accounted for 59.89% of the total genus-level composition. Panel (**B**) displays proportional composition within the selected top-ten subset and therefore does not represent the complete genus-level community. Taxonomic groups that could not be resolved to the genus level are reported at the lowest reliably assigned taxonomic level. Relative abundances represent compositional rather than absolute abundances. F, female; M, male.

Substantial interindividual variability was observed in the relative abundances of the dominant taxonomic groups. This was particularly evident for *Prevotella*, which was detected in 15 of the 20 participants (75.0%), had a median relative abundance of 0.07%, and reached a maximum of 22.72%. By contrast, each of the other nine dominant taxonomic groups was detected in all participants. The mean relative abundances and participant-level distributions of the ten dominant taxonomic groups are shown in Figure 2, and their complete descriptive statistics are reported in Supplementary Table S10.

### 3.4. Alpha-Diversity Profile, Sequencing-Depth Diagnostics, and Beta-Diversity Ordination

Independent recalculation from the exported OTU count table reproduced the MicrobAT-reported observed richness exactly and reproduced Shannon diversity and Pielou’s evenness for all 20 participants within the two-decimal precision of the software output. Full-precision independently recalculated values were therefore used in the revised alpha-diversity analyses. Shannon diversity was positively associated with classified OTU-read depth according to Pearson correlation on both the original depth scale (r = 0.651; *p* = 0.0019) and the log10-transformed scale (r = 0.585; *p* = 0.0067). However, the corresponding Spearman correlation did not support a monotonic association (ρ = 0.224; *p* = 0.342). Pielou’s evenness was not associated with classified OTU-read depth according to Pearson correlation on either the original scale (r = −0.021; *p* = 0.931) or the log10-transformed scale (r = −0.233; *p* = 0.323), or according to Spearman correlation (ρ = −0.250; *p* = 0.289).

After 1000 repeated rarefactions to the common depth of 3026 classified OTU reads, concordance between full-depth and mean rarefied values was very high for Shannon diversity (Pearson’s r = 0.999; Spearman’s ρ = 0.998) and more moderate for Pielou’s evenness (Pearson’s r = 0.609; Spearman’s ρ = 0.723). Individual Shannon rarefaction curves and the concordance between full-depth and mean rarefied alpha-diversity estimates are shown in Supplementary Figure S5.

The Shannon index, designated as the primary microbiome outcome, had a mean value of 3.99 ± 0.27, a median of 4.09 (IQR, 3.93–4.15), and a range of 3.13–4.27. Pielou’s evenness, considered a complementary alpha-diversity measure, had a mean value of 0.647 ± 0.025, a median of 0.652 (IQR, 0.639–0.660), and a range of 0.571–0.695. Both measures showed relatively limited dispersion across participants. Descriptive statistics are reported in Table 2, and participant-level classified OTU-read depths, observed OTU richness, and full-precision alpha-diversity values are provided in Supplementary Table S11.

**Figure 3:**
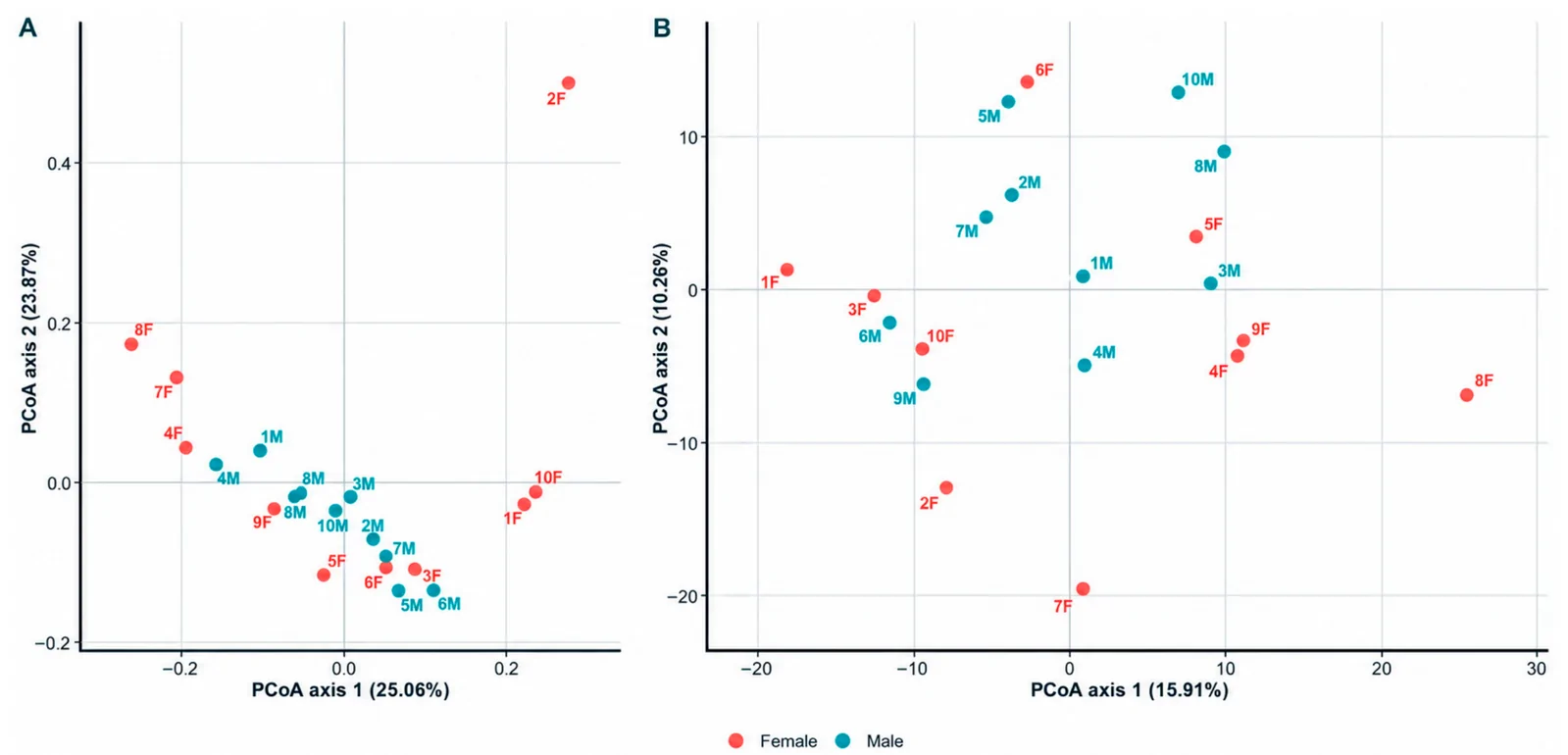
Principal coordinates analysis of between-participant gut microbiota composition. (**A**) PCoA based on Bray–Curtis dissimilarity calculated from observed genus-level relative abundances. (**B**) PCoA based on Aitchison distance calculated in centered log-ratio space after adding a proportional pseudocount of 1 × 10^−6^. Each point represents one participant, and participant identifiers are displayed next to the corresponding points. Colors identify sex for visualization only and do not represent inferential groupings. Percentages in the axis labels indicate the proportion of variation represented by each coordinate axis. F, female; M, male; PCoA, principal coordinates analysis.

Principal coordinates analysis was used to visualize between-participant differences in overall microbial community composition. For Bray–Curtis dissimilarity, the first and second PCoA axes represented 25.06% and 23.87% of the total variation, respectively, corresponding to a cumulative proportion of 48.93%. For Aitchison distance, the first and second axes represented 15.91% and 10.26%, respectively, corresponding to a cumulative proportion of 26.17%. Visual inspection of both ordinations indicated continuous interindividual compositional variation without clearly separated clusters, although some participants occupied relatively peripheral positions in the ordination spaces (Figure 3). Participant-level PCoA coordinates are reported in Supplementary Table S12.

### 3.5. Prespecified Genera and the Prevotella/Bacteroides Compositional Gradient

All eight bacterial genera prespecified for exploratory taxon-level analyses were detected in the microbiome dataset. *Bacteroides* had the highest mean relative abundance (15.34%), followed by *Faecalibacterium* (7.69%), *Bifidobacterium* (3.56%), *Blautia* (3.50%), and *Prevotella* (3.24%). *Roseburia* and *Ruminococcus* each had a mean relative abundance of 1.25%, whereas *Akkermansia* had the lowest mean relative abundance (0.58%). Seven of the eight genera were detected in all participants. *Prevotella* was detected in 15 participants (75.0%) and showed the greatest distributional asymmetry, with a mean relative abundance of 3.24%, a median of 0.07%, and a range of 0–22.72%. Descriptive statistics for the eight genera are reported in Table 3, and participant-level relative abundances are provided in Supplementary Table S13. For descriptive visualization, the participant-level distribution of the eight genera after transformation as log10(relative abundance [%] + 0.01) is shown in Supplementary Figure S2.

The untransformed *Prevotella*/*Bacteroides* ratio was strongly right-skewed, with a mean of 0.399 ± 0.791, a median of 0.00454 (IQR, 0.000060–0.191), and a range of 0–2.60. After addition of the prespecified pseudocount and natural-log transformation, the ratio had a mean of −5.253 ± 4.164, a median of −6.342 (IQR, −8.614 to −1.726), and a range of −10.396 to 0.955. Seventeen participants (85.0%) had an untransformed ratio below 1 and were descriptively classified as showing relative *Bacteroides* predominance, whereas three participants (15.0%; 1M, 4M, and 7F) had a ratio above 1 and showed relative *Prevotella* predominance. No participant had equal observed abundances of the two genera.

**Figure 4:**
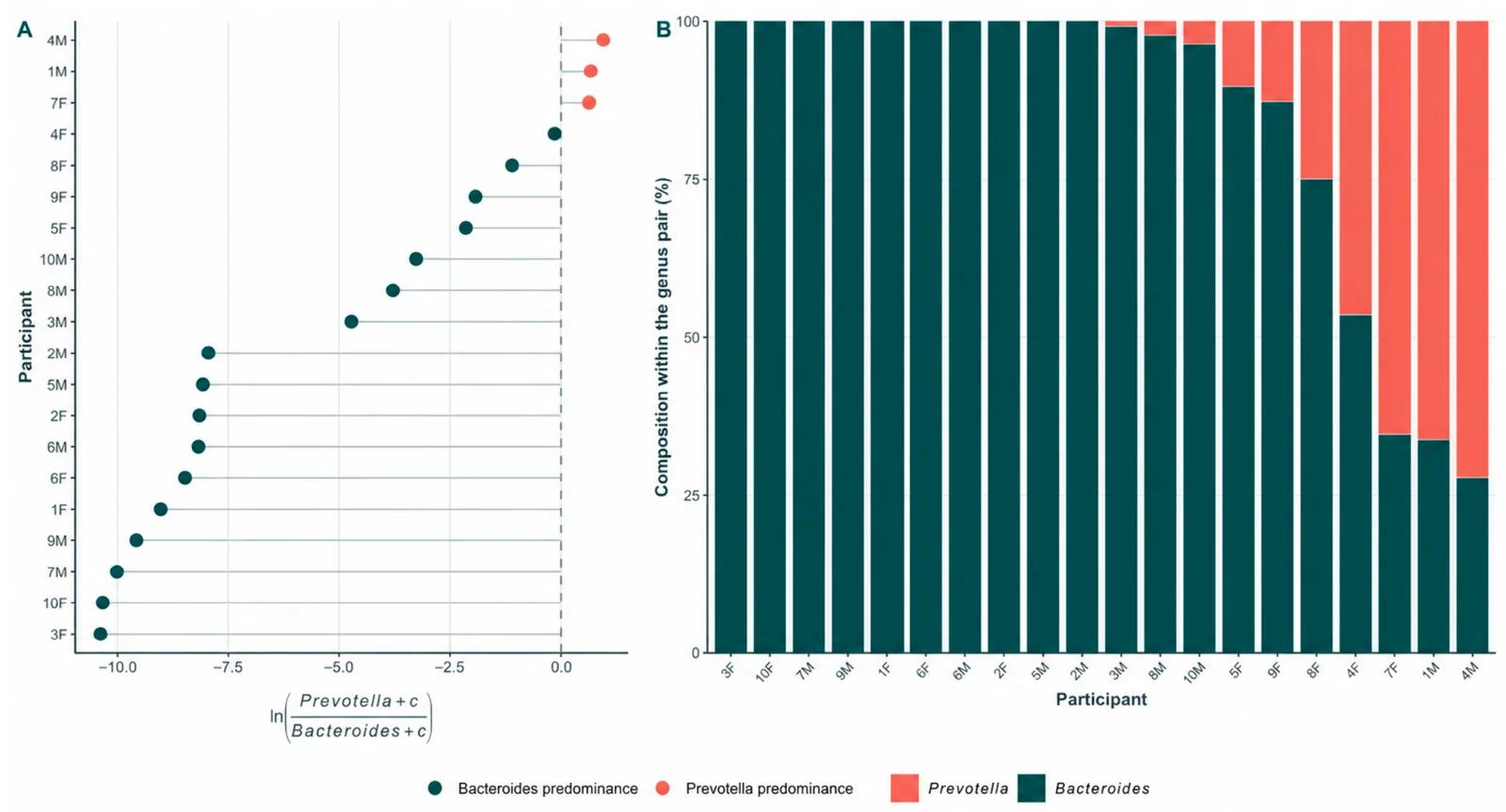
Continuous *Prevotella*/*Bacteroides* compositional gradient across the 20 participants. (**A**) Participants ordered according to increasing values of $\ln\left[\frac{Prevotella+c}{Bacteroides+c}\right]$, where c = 8.262619 × 10^−6^ on the proportional-abundance scale. The dashed vertical line at zero represents equal abundances of the two genera, and point colors indicate descriptive predominance based on the untransformed P/B ratio. (**B**) Composition within the *Bacteroides*–*Prevotella* pair, calculated from the observed relative abundances of the two genera and expressed as their respective percentages of the pairwise sum. Each column represents one participant and totals 100%. The absence of a *Prevotella* segment indicates an observed abundance of zero rather than a missing value. Within-pair percentages do not represent the abundances of the two genera relative to the complete microbial community. Predominance categories are descriptive and should not be interpreted as formal enterotypes. P/B, *Prevotella*/*Bacteroides*.

Ordering participants according to the natural-log-transformed ratio showed a continuous compositional gradient rather than a separation into discrete groups (Figure 4A). The within-pair composition showed a progressive increase in the proportional contribution of *Prevotella* across this gradient (Figure 4B). In participants in whom *Prevotella* was not detected, the genus pair was composed entirely of *Bacteroides*; the absence of a *Prevotella* segment therefore represents an observed relative abundance of zero rather than a missing value. These predominance categories were used only for descriptive purposes and were not interpreted as formal enterotypes. Participant-level abundances, untransformed ratios, natural-log-transformed ratios, and descriptive predominance categories are reported in Supplementary Table S14.

### 3.6. Associations of Dietary and Lifestyle Exposures with Alpha Diversity

Associations of the four continuous dietary and lifestyle exposures with the Shannon index and Pielou’s evenness were evaluated using separate ordinary least-squares linear regression models adjusted for age and sex. None of the eight exposure coefficients had a conventional 95% confidence interval excluding zero or a nominal *p*-value below 0.05. Nominal *p*-values ranged from 0.127 to 0.684, and all FDR-adjusted *p*-values were 0.684 after Benjamini–Hochberg correction across the eight alpha-diversity comparisons (Table 4).

For the Shannon index, the largest exposure-specific effect, as quantified by partial *R*^2^, was observed for physical activity. Each additional MET-min/week was associated with a mean difference of −5.82 × 10^−5^ in the Shannon index (95% CI, −1.35 × 10^−4^ to 1.84 × 10^−5^; *p* = 0.127; partial *R*^2^ = 0.140), corresponding to an estimated difference of −0.058 per additional 1000 MET-min/week. The partial *R*^2^ values for MEDAS, UPF score, and the adapted Sleep score were 0.026, 0.011, and 0.026, respectively. All corresponding confidence intervals were compatible with effects in either direction.

For Pielou’s evenness, the largest exposure-specific effect was observed for the adapted Sleep score. Each one-point increase was associated with a mean difference of 0.00220 in Pielou’s evenness (95% CI, −0.00158 to 0.00597; *p* = 0.235; partial *R*^2^ = 0.087). The partial *R*^2^ values for MEDAS, UPF score, and physical activity were 0.058, 0.014, and 0.049, respectively. These estimates were imprecise and were not interpreted as evidence of directional associations.

The complete models explained 15.4–26.4% of the observed variance in the Shannon index and 12.0–18.5% of the variance in Pielou’s evenness; adjusted *R*^2^ values ranged from −0.005 to 0.126 and from −0.045 to 0.032, respectively. No problematic collinearity was identified. The Breusch–Pagan test yielded nominal *p*-values above 0.05 in all eight models, whereas the Shapiro–Wilk test yielded a nominal *p*-value below 0.05 only for the MEDAS–Shannon model (*p* = 0.032). At least one observation exceeded the Cook’s-distance screening threshold of 4/n = 0.20 in each model. The largest Cook’s-distance value was 1.264 in the UPF–Shannon model and involved participant 4M; nevertheless, no participant was excluded from the primary analyses. Leave-one-out analyses produced a coefficient sign reversal in four of the eight models, predominantly for estimates close to zero, but no leave-one-out iteration yielded a nominal exposure *p*-value below 0.05.

No association was supported after FDR correction using either HC3-based or Freedman–Lane permutation inference. Use of the original untruncated IPAQ-SF score did not materially alter the estimates or conclusions. The overall interpretation also remained unchanged when the models were repeated using alpha-diversity values averaged across 1000 rarefactions to 3026 classified OTU reads (minimum *p*-FDR = 0.495) or after exclusion of participant 7F, who had the lowest classified OTU-read depth (minimum *p*-FDR = 0.502). Detailed model-fit, diagnostic, and robustness results are reported in Supplementary Table S15.

### 3.7. Associations of Dietary and Lifestyle Exposures with Overall Gut Microbiota Composition

Associations of the four continuous dietary and lifestyle exposures with overall gut microbiota composition were evaluated using separate age- and sex-adjusted permutational multivariate analyses of variance (PERMANOVA) based on Bray–Curtis and Aitchison distances. None of the eight marginal exposure effects had a nominal permutation-derived *p*-value below 0.05. After Benjamini–Hochberg correction across the eight beta-diversity comparisons, all FDR-adjusted *p*-values were 0.800 (Table 5).

For Bray–Curtis dissimilarity, the marginal proportion of community variation attributable to the exposures after adjustment for age and sex ranged from 3.42% to 6.49%. Physical activity showed the largest marginal effect (marginal *R*^2^ = 0.0649; pseudo-F = 1.246; *p* = 0.247), followed by the UPF score (marginal *R*^2^ = 0.0435; pseudo-F = 0.815; *p* = 0.560), MEDAS (marginal *R*^2^ = 0.0348; pseudo-F = 0.646; *p* = 0.784), and the adapted Sleep score (marginal *R*^2^ = 0.0342; pseudo-F = 0.633; *p* = 0.800). The corresponding complete models, including the exposure, age, and sex, accounted for 13.63–16.70% of the total Bray–Curtis variation.

For Aitchison distance, marginal *R*^2^ values ranged from 0.0454 to 0.0585. The largest marginal effect was observed for the adapted Sleep score (marginal *R*^2^ = 0.0585; pseudo-F = 1.114; *p* = 0.275), followed by physical activity (marginal *R*^2^ = 0.0477; pseudo-F = 0.897; *p* = 0.646), the UPF score (marginal *R*^2^ = 0.0471; pseudo-F = 0.885; *p* = 0.654), and MEDAS (marginal *R*^2^ = 0.0454; pseudo-F = 0.850; *p* = 0.741). The corresponding complete models accounted for 14.62–15.94% of the total Aitchison variation. Within this cross-sectional pilot dataset, none of the four exposures therefore showed a statistically supported association with overall microbial community composition. Given the small sample, the observed marginal *R*^2^ values should be regarded as imprecise exploratory effect-size estimates that may inform, but should not independently determine, the design of future studies.

Diagnostic tests based on the operational exposure categories did not identify statistically supported differences in multivariate dispersion, either before or after correction for multiple testing. FDR-adjusted *p*-values ranged from 0.643 to 0.995. The smallest nominal *p*-value was observed for Bray–Curtis dispersion across MEDAS adherence categories (*p* = 0.080; *p*-FDR = 0.643). However, because the smallest MEDAS category contained only two participants, this diagnostic comparison had limited sensitivity and should be interpreted cautiously.

The interpretation remained unchanged when the analyses were repeated after retaining only the 161 genus-level features detected in at least four participants, when Aitchison distances were recalculated using pseudocounts of 1 × 10^−8^ and 1 × 10^−4^, and when the original untruncated IPAQ-SF score was used. Across all sensitivity specifications, no marginal exposure effect had an FDR-adjusted *p*-value below 0.05. Complete-model results, multivariate-dispersion diagnostics, and all beta-diversity sensitivity analyses are reported in Supplementary Table S16.

### 3.8. Associations of Dietary and Lifestyle Exposures with Prespecified Bacterial Genera

Associations of the four continuous dietary and lifestyle exposures with the centered log-ratio (CLR) coordinates of the eight prespecified genera were evaluated using 32 separate age- and sex-adjusted linear regression models. None of the conventional 95% confidence intervals excluded zero. Nominal *p*-values ranged from 0.056 to 0.994, and FDR-adjusted *p*-values ranged from 0.815 to 0.994; therefore, none of the associations was statistically supported after correction for multiple testing (Table 6).

The largest exposure-specific effect was observed for the association between the UPF score and *Akkermansia*. Each one-point increase in the UPF score was associated with a −0.314 change in the CLR coordinate of *Akkermansia* (95% CI, −0.638 to 0.009; *p* = 0.056; *p*-FDR = 0.815; partial *R*^2^ = 0.210). Because the outcome was a CLR coordinate, this coefficient represents a change in the abundance of *Akkermansia* relative to the geometric mean abundance of the complete genus-level composition and should not be interpreted as a change in its absolute abundance.

**Figure 5:**
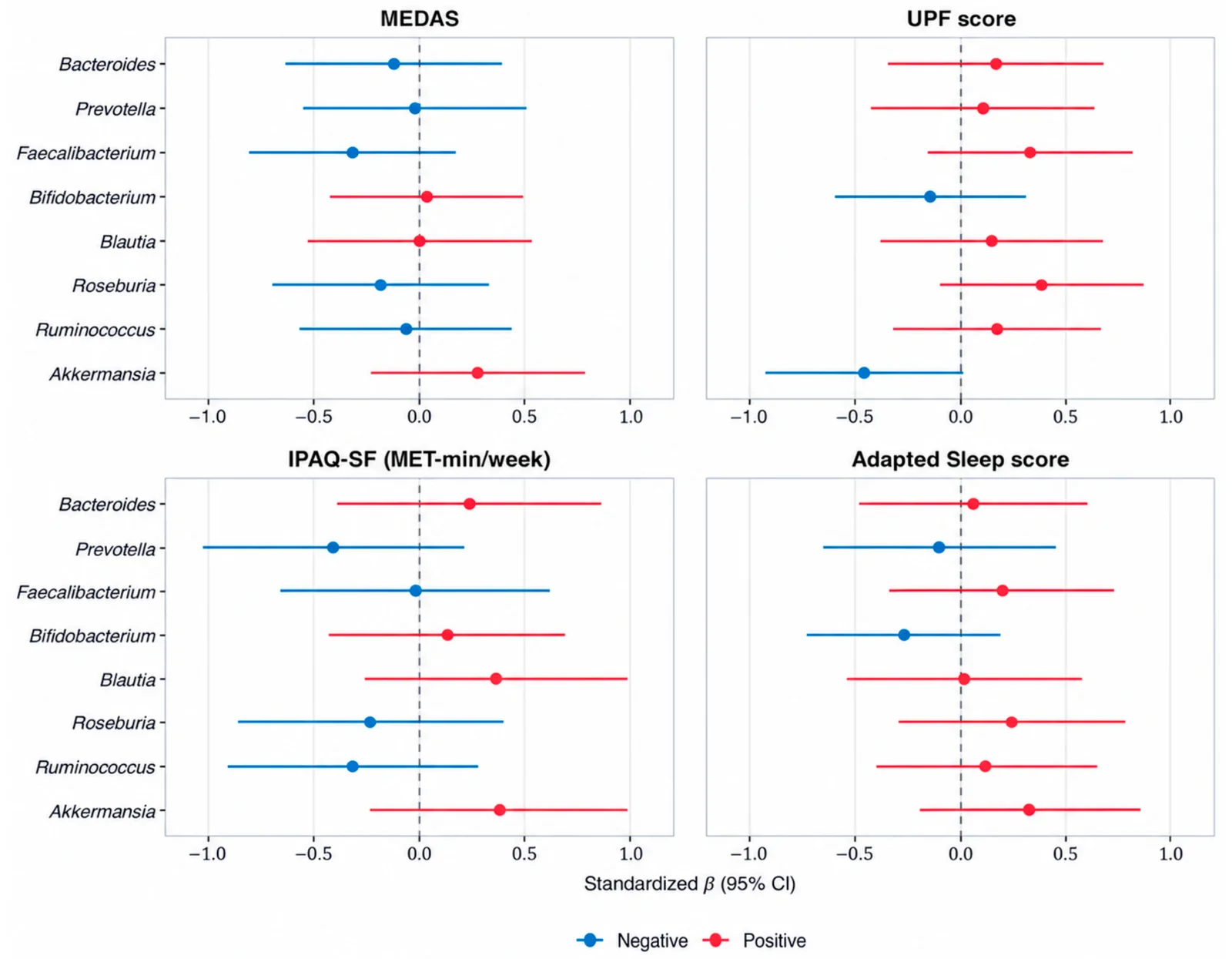
Standardized associations of dietary and lifestyle exposures with the CLR coordinates of the eight prespecified bacterial genera. Points represent standardized exposure coefficients from separate linear regression models adjusted for age and sex, and horizontal lines represent conventional 95% confidence intervals. Standardized coefficients were obtained by expressing the exposure and genus-specific CLR outcome in standard-deviation units, thereby permitting comparison of effect direction and magnitude across exposures and genera. Red indicates positive coefficients and blue indicates negative coefficients; the dashed vertical line denotes no association. All confidence intervals included zero, and none of the 32 associations was statistically supported after Benjamini–Hochberg correction. Standardization was used exclusively for graphical presentation; the unstandardized coefficients used for inference are reported in Table 6. CI, confidence interval; CLR, centered log-ratio; IPAQ-SF, International Physical Activity Questionnaire–Short Form; MEDAS, Mediterranean Diet Adherence Screener; MET, metabolic equivalent of task; UPF, ultra-processed food.

Other comparatively large, although statistically unsupported, partial *R*^2^ values were observed for the positive association between the UPF score and *Roseburia* (β = 0.169; 95% CI, −0.042 to 0.380; *p* = 0.110; partial *R*^2^ = 0.152), the positive association between the UPF score and *Faecalibacterium* (β = 0.056; 95% CI, −0.026 to 0.138; *p* = 0.167; partial *R*^2^ = 0.116), and the negative association between physical activity and *Prevotella* (β = −8.98 × 10^−4^ per MET-min/week; 95% CI, −2.27 × 10^−3^ to 4.75 × 10^−4^; *p* = 0.185; partial *R*^2^ = 0.107). The latter coefficient corresponds to an estimated change of −0.898 in the *Prevotella* CLR coordinate per additional 1000 MET-min/week. The largest partial *R*^2^ values involving MEDAS and the adapted Sleep score were observed for *Faecalibacterium* (MEDAS: β = −0.109; *p* = 0.189; partial *R*^2^ = 0.105) and *Akkermansia* (adapted Sleep score: β = 0.253; *p* = 0.201; partial *R*^2^ = 0.100), respectively. The standardized exposure coefficients and their conventional 95% confidence intervals are summarized in Figure 5, whereas the exposure-specific partial *R*^2^ values and the directions of the corresponding adjusted associations are presented in Supplementary Figure S3.

The Shapiro–Wilk test yielded nominal *p*-values below 0.05 in eight of the 32 primary models, while the Breusch–Pagan test indicated potential heteroscedasticity in one model, involving MEDAS and *Bacteroides*. No problematic collinearity was detected. Thirty models contained at least one observation exceeding the prespecified Cook’s-distance screening threshold of 4/n = 0.20, although no Cook’s-distance value exceeded 1.00. Leave-one-out analyses produced a coefficient sign reversal in 14 models, predominantly among associations with estimates close to zero, illustrating the limited stability expected in this small pilot sample. No participant was removed from the primary analyses.

For the UPF–*Akkermansia* association, HC3-based and Freedman–Lane permutation analyses produced nominal *p*-values of 0.044 and 0.049, respectively. However, the corresponding FDR-adjusted values were 0.982 and 0.811, and no association was supported after multiplicity correction under either inferential approach. The overall interpretation also remained unchanged when taxonomic abundance was represented using arcsine-square-root-transformed, natural-log-transformed, or untransformed relative abundances; when CLR coordinates were recalculated using pseudocounts of 1 × 10^−8^ and 1 × 10^−4^; and when the original untruncated IPAQ-SF score was used. Diagnostic and robustness summaries are reported in Supplementary Table S17.

### 3.9. Associations of Dietary and Lifestyle Exposures with the Prevotella/Bacteroides Log-Ratio

Associations of the four continuous dietary and lifestyle exposures with the natural-log-transformed *Prevotella*/*Bacteroides* ratio were evaluated using separate age- and sex-adjusted linear regression models. None of the conventional 95% confidence intervals excluded zero. Nominal *p*-values ranged from 0.183 to 0.859, and FDR-adjusted *p*-values ranged from 0.732 to 0.859; therefore, none of the four associations was statistically supported after correction for multiple testing (Table 7).

Physical activity showed the largest exposure-specific partial *R*^2^. Each additional 1000 MET-min/week was associated with an estimated −0.842 change in the natural-log-transformed *Prevotella*/*Bacteroides* ratio (95% CI, −2.125 to 0.441; *p* = 0.183; *p*-FDR = 0.732; partial *R*^2^ = 0.108). The negative coefficient indicated a lower relative balance of *Prevotella* to *Bacteroides* with increasing physical activity, but the estimate was imprecise and was not statistically supported. The corresponding estimates were smaller for MEDAS (β = 0.103; 95% CI, −1.102 to 1.307; partial *R*^2^ = 0.002), the UPF score (β = 0.096; 95% CI, −0.496 to 0.688; partial *R*^2^ = 0.007), and the adapted Sleep score (β = −0.178; 95% CI, −0.864 to 0.509; partial *R*^2^ = 0.018).

The Shapiro–Wilk test yielded nominal *p*-values below 0.05 for the MEDAS and UPF models (*p* = 0.036 and *p* = 0.037, respectively), whereas no evidence of heteroscedasticity was identified by the Breusch–Pagan test in any of the four models. The maximum variance inflation factor was 1.530, indicating no problematic collinearity. The UPF and adapted Sleep score models contained observations exceeding the Cook’s-distance screening threshold of 0.20: participant 4M in the UPF model (Cook’s distance = 1.210) and participant 4F in the Sleep model (Cook’s distance = 0.528). Leave-one-out analyses produced coefficient sign reversals for the MEDAS, UPF, and adapted Sleep score models, whereas the physical-activity coefficient remained negative after every participant was omitted in turn. No participant was excluded from the primary analyses.

HC3-based and Freedman–Lane permutation analyses did not materially alter the interpretation. HC3 *p*-values ranged from 0.135 to 0.874 and their FDR-adjusted values from 0.540 to 0.874; permutation *p*-values ranged from 0.178 to 0.855 and their FDR-adjusted values from 0.712 to 0.855. The physical-activity estimate also remained negative when the original untruncated IPAQ-SF score was used (β = −6.76 × 10^−4^ per MET-min/week; 95% CI, −1.91 × 10^−3^ to 5.57 × 10^−4^; *p* = 0.262; partial *R*^2^ = 0.078). Finally, temporary exclusion of participant 7F, who had the lowest sequencing depth, preserved the direction of all four coefficients; none of the resulting confidence intervals excluded zero, and all FDR-adjusted *p*-values remained ≥0.452. Complete model-fit, diagnostic, influence, and robustness results are reported in Supplementary Table S18.

### 3.10. Scenario-Based Sample-Size and Future-Study Design Benchmarks

**Figure 6:**
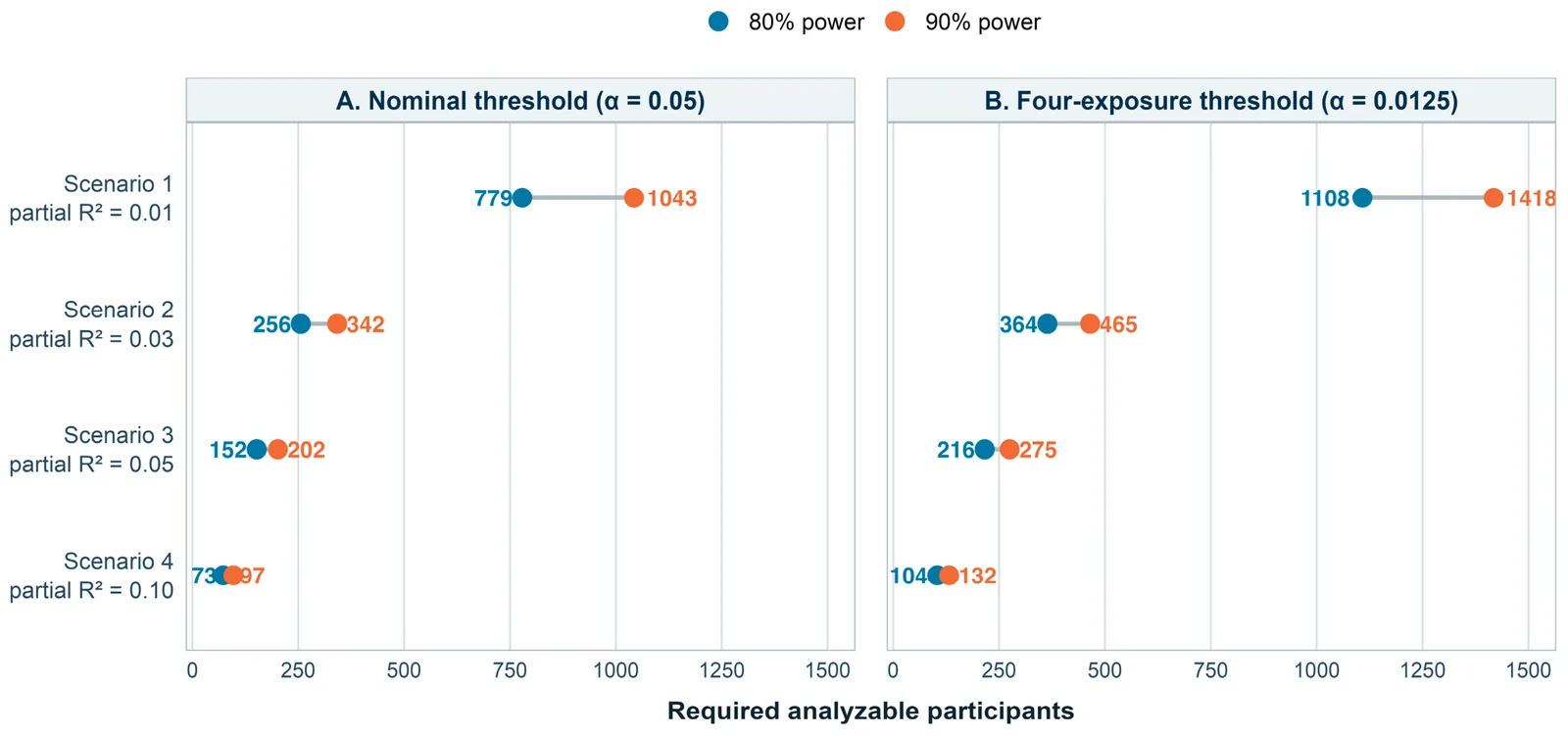
Required analyzable sample sizes for age- and sex-adjusted cross-sectional linear regression analyses across assumed effect-size, power, and multiplicity scenarios. Each horizontal row represents one assumed exposure-specific partial *R*^2^ value, and the labelled points indicate the corresponding number of analyzable participants required for 80% or 90% statistical power. Panel (**A**) presents calculations based on a nominal two-sided α of 0.05. Panel (**B**) presents the more conservative four-exposure scenario based on α = 0.0125. Grey connecting lines are included solely to facilitate comparison between the two power levels within the same effect-size scenario. Under the conservative planning assumptions of 90% power, α = 0.0125, and partial *R*^2^ values of 0.03–0.05, the required analyzable sample ranged from 275 to 465 participants. These values are scenario-based cross-sectional regression benchmarks and do not represent formal power estimates for PERMANOVA or longitudinal models.

The scenario-based calculations for age- and sex-adjusted cross-sectional linear regression models showed that the required number of analyzable participants depended strongly on the assumed exposure-specific partial *R*^2^, target power, and significance threshold. Across partial *R*^2^ scenarios of 0.01, 0.03, 0.05, and 0.10, the required sample sizes under the nominal α of 0.05 ranged from 73 to 779 participants at 80% power and from 97 to 1043 participants at 90% power. Under the more conservative four-exposure threshold of α = 0.0125, the corresponding requirements ranged from 104 to 1108 participants at 80% power and from 132 to 1418 participants at 90% power (Table 8; Figure 6). These estimates apply specifically to exposure-coefficient tests in the stated cross-sectional regression model and do not represent formal sample-size requirements for PERMANOVA or longitudinal analyses.

Under the conservative planning scenario of 90% power and α = 0.0125, assumed partial *R*^2^ values of 0.03 and 0.05 corresponded to 465 and 275 analyzable participants, respectively. The resulting range of 275–465 analyzable participants was retained as the principal benchmark for detecting modest exposure-specific associations using an age- and sex-adjusted cross-sectional linear regression model. This interval is based on assumed rather than pilot-derived effect sizes and should be interpreted as a scenario-based planning range, not as a definitive sample-size requirement for a longitudinal study or a community-composition analysis.

After allowing for 10–20% attrition, incomplete assessments, unsuccessful sequencing, or other sources of unusable observations, the enrollment target corresponding to the principal cross-sectional regression benchmark increased to 306–582 participants. If three stool collections were planned for each enrolled participant, this target would correspond to an operational workload of 918–1746 specimens; with four collections, the corresponding workload would be 1224–2328 specimens. These specimen counts quantify collection and laboratory workload only and do not incorporate an assumed efficiency gain from repeated measurements. Complete combinations of assumed effect size, power, significance threshold, anticipated data loss, enrollment target, and specimen workload are reported in Supplementary Table S19.

Calculations based directly on the exposure-specific effect estimates observed in the pilot linear-regression analyses produced substantially heterogeneous sample-size requirements. Across the alpha-diversity, CLR-transformed genus-level, and natural-log-transformed *Prevotella*/*Bacteroides* ratio outcomes, finite estimates ranged from 32 to 32,720 analyzable participants under the 80%–power, α = 0.05 scenario and from 58 to 59,541 participants under the 90%–power, α = 0.0125 scenario. One near-zero pilot effect produced a requirement exceeding 2,000,000 analyzable participants and is therefore represented by a threshold symbol in Supplementary Figure S4. This extreme variability illustrates the instability of model-specific planning based on effect estimates obtained from a small pilot sample. Accordingly, these values were treated exclusively as exploratory benchmarks, and the assumed partial *R*^2^ range of 0.03–0.05, rather than the individual pilot estimates, formed the principal basis for prospective planning. Each model-specific estimate is identified by microbiome outcome and exposure in Supplementary Figure S4, and the corresponding calculations are reported in Supplementary Table S20. No formal sample-size estimates were derived from the PERMANOVA results; a future study in which community composition is the primary outcome should instead use a dedicated simulation-based power analysis reflecting the intended distance measure, multivariate covariance and dispersion structures, exposure distribution, covariates, permutation strategy, and repeated-measures design.

Because within-participant temporal variability, temporal correlation, and changes in the exposures could not be estimated from the present cross-sectional dataset, repeated sampling was not assumed to reduce the required number of participants. Repeated collections were incorporated exclusively into the specimen-workload calculations. Accordingly, the participant-level estimates should be interpreted as cross-sectional regression planning benchmarks that require refinement using empirically supported repeated-measures assumptions before implementation in a definitive longitudinal protocol. If overall community composition is selected as a primary outcome, its sample size should be determined separately using a dedicated simulation-based PERMANOVA power analysis that reflects the intended distance measure, multivariate covariance structure, exposure distribution, covariates, permutation strategy, and repeated-measures design.

## 4. Discussion

This cross-sectional pilot study examined four potentially modifiable dietary and lifestyle exposures across complementary levels of gut microbiome organization in 20 healthy adults. No association was statistically supported after false-discovery-rate correction: all conventional confidence intervals for the univariate outcomes included zero, and none of the exposure effects on overall community composition had a nominal permutation-derived *p*-value below 0.05. The estimates were not, however, uniformly close to zero. Exposure-specific partial *R*^2^ values reached 0.140 for alpha diversity, 0.210 for a prespecified genus, and 0.108 for the *Prevotella*/*Bacteroides* log-ratio; marginal *R*^2^ values for community composition reached 0.0649 for Bray–Curtis dissimilarity and 0.0585 for Aitchison distance. These estimates were accompanied by substantial uncertainty and, in several univariate models, sensitivity to individual observations. The findings therefore do not demonstrate the absence of diet– or lifestyle–microbiota relationships. Rather, they define the magnitude, uncertainty, and stability of the signals observable in this pilot sample and provide the empirical basis for a more definitive longitudinal design.

The absence of statistically supported associations in this study is compatible with, rather than contradictory to, evidence from larger and more deeply phenotyped cohorts. In 10,068 participants from the Human Phenotype Project, app-based dietary records combined with shotgun metagenomics predicted microbial richness, Shannon diversity, and the relative abundance of most evaluated species and pathways; the degree of food processing also emerged as a predictor of microbial diversity and composition [37]. In approximately 1000 participants from the Food & You digital cohort, high-resolution real-time dietary logging showed that both diet quality and its day-to-day regularity were associated with alpha diversity [38]. These studies had substantially larger samples, wider exposure contrasts, more detailed dietary characterization, and greater taxonomic or functional resolution than the present pilot. In the current cohort, most participants had intermediate or high MEDAS adherence, and none belonged to the high-UPF category, thereby limiting contrast at the upper end of the exposure distributions. A recent cross-sectional study analysing 82 fecal samples from medical students associated NOVA-defined UPF consumption with differences in community composition and selected genera, but used a different exposure definition, population, and analytical framework [39]. Measurement resolution is also relevant to the behavioral exposures: accelerometer-derived activity in 8416 SCAPIS participants was associated with numerous microbial species and functional pathways [40], whereas a study of 159 adults found no direct association between sleep quality and alpha diversity but reported differences in beta diversity and selected microbial features [41]. Collectively, these findings reinforce that alpha diversity, community composition, and individual taxa represent distinct microbiome properties and need not show concordant associations with the same exposure.

The taxon-specific results identify hypotheses for future prespecification, not established biological effects. The most prominent estimate was the negative association between the UPF score and the Akkermansia CLR coordinate (partial *R*^2^ = 0.210). Although HC3 and Freedman–Lane analyses yielded nominal *p*-values below 0.05, the conventional 95% confidence interval included zero and the association was not supported after correction across the 32 exposure–genus comparisons under any inferential approach. Moreover, considered together with the negative *Akkermansia* coefficient, the positive UPF coefficients for *Roseburia* and *Faecalibacterium* did not form a coherent taxonomic pattern from which a mechanism could be inferred. Physical activity showed negative coefficients for both the *Prevotella* CLR coordinate and the *Prevotella*/*Bacteroides* log-ratio. The latter retained a negative direction throughout the leave-one-out analyses and when the original untruncated IPAQ-SF score was used, but its confidence interval remained compatible with effects in either direction. Similarly, the descriptive predominance of Bacteroides in 17 of 20 participants should not be interpreted as evidence of discrete enterotypes: the participant-level distribution supported a continuous *Prevotella*/*Bacteroides* gradient. Because CLR coordinates and log-ratios quantify relative balances within a composition, these coefficients do not represent changes in absolute bacterial load. Large-scale benchmarking has shown that taxon-level conclusions can vary with preprocessing and analytical method [42]. The use of CLR coordinates, alternative transformations, alternative pseudocounts, and influence analyses reduced dependence on a single specification, but the resulting estimates remain exploratory.

Several design and analytical features strengthen the study’s value as a pilot. Participants were selected without replacement through sex-stratified random sampling from the eligible cohort. Stool production and completion of the dietary and lifestyle questionnaire were coordinated on the same day, while collection, refrigerated transport, laboratory processing, and sequencing followed standardized procedures. One fresh-stool profile was retained per participant, avoiding pseudoreplication, and complete exposure, covariate, and microbiome data were available for all 20 participants. The analytical hierarchy distinguished the primary Shannon outcome from complementary alpha-diversity, community-level, genus-level, and log-ratio outcomes. The OTU-level Shannon and Pielou values were independently recalculated from the exported count table, reproducing observed richness exactly and reproducing the software-reported diversity values within their displayed precision. The potential influence of sequencing depth was evaluated directly through correlation analyses, repeated rarefaction, and exclusion of the lowest-depth participant. Taxonomic data were treated compositionally; multiplicity was controlled within prespecified microbiome domains; and no participant was removed solely because of a diagnostic measure. Conventional, HC3-based, and permutation-based inference, together with sensitivity analyses involving sequencing depth, transformations, pseudocounts, prevalence filtering, IPAQ-SF scoring, and influential observations, produced the same overall interpretation. This convergence supports the internal consistency of the analytical conclusions, although it does not eliminate uncertainty in the individual effect estimates.

A strength of the present study is the deliberate restriction to a well-characterized population of young, healthy adults. The eligibility-screening procedure excluded major conditions and exposures known to affect the gut microbiota, including chronic systemic or gastrointestinal disease, regular medication use, recent antibiotic exposure, recent use of microbiota-modulating supplements, gastrointestinal infection, acute gastrointestinal symptoms, and major recent dietary changes. These factors were therefore controlled primarily through study design, reducing important sources of biological heterogeneity and strengthening internal comparability. Within this defined population, the analytical sample of 20 participants and the relatively narrow exposure distributions nevertheless limited the precision of individual association estimates and the feasibility of extensive multivariable adjustment. Adjustment was restricted to the prespecified covariates age and sex to preserve model parsimony and limit overfitting. Residual confounding cannot be entirely excluded because adiposity or body composition, stool consistency, bowel frequency or intestinal transit, total energy intake, and other potentially relevant host or intestinal characteristics were not measured in sufficient detail for multivariable adjustment. Accordingly, the observed effect sizes are interpreted as exploratory estimates for hypothesis prioritization and future-study planning rather than as confirmatory population effects.

The cross-sectional design additionally precludes determination of temporal directionality. Exposure assessment was not delayed relative to specimen production: selected participants completed the questionnaire on the day they produced the stool sample for laboratory delivery. The measures nevertheless covered different reference periods—habitual dietary behavior, physical activity during the preceding seven days, and sleep during the preceding month—which is appropriate to their respective constructs but should be considered when comparing exposure domains. MEDAS and IPAQ-SF were established screening instruments, whereas the study-specific UPF score measured reported consumption frequency rather than intake by weight or energy contribution, and the adapted sleep score was not the complete validated PSQI. These latter measures should therefore be regarded as structured exploratory exposures. Collectively, these considerations define the appropriate scope of inference without diminishing the value of the standardized sampling procedures, carefully selected cohort, and comprehensive analytical and robustness framework, or the study’s methodological contribution to the design of a larger longitudinal investigation.

The microbiome framework was well aligned with the community-level and genus-level objectives of this pilot study. Standardized extraction and sequencing procedures generated usable taxonomic profiles for all 20 selected fresh-stool samples, and no extraction, library-preparation, or sequencing failure was recorded among the 160 participant-derived profiles generated in the source laboratory study. Blank and negative extraction controls were included in each sequencing run and evaluated for unexpected amplification or taxonomic signals as part of routine laboratory quality assurance, while mock-community controls had been assessed during preliminary workflow verification. No evidence of contamination requiring rejection of a sequencing run, repetition or exclusion of a participant sample, or removal of individual taxa was identified. As quality-control materials, these controls were not part of the participant-level analytical dataset, and their evaluation did not trigger any corrective action.

The exported OTU count table enabled independent recalculation and verification of observed richness, Shannon diversity, and Pielou’s evenness, whereas the exported taxonomic tables supported complete reproduction of the downstream compositional, statistical, diagnostic, and sensitivity analyses. The main analytical results were therefore not dependent on the rounded diversity values reported by the commercial software or on an undocumented downstream transformation. The proprietary nature of the primary MicrobAT workflow nevertheless prevented complete independent reproduction of the upstream processing because its internal clustering, read-filtering, chimera-handling, classification, and reference-database settings were not included in the exported output. This represents a clearly defined reproducibility boundary for the primary proprietary pipeline rather than a limitation of the independently implemented downstream analyses.

The use of a single 16S rRNA gene profile per participant means that within-participant temporal variability could not be characterized, and the absence of absolute microbial-load measurements requires taxonomic findings to be interpreted as relative rather than absolute abundances. These boundaries define the intended resolution of the present pilot without detracting from its standardized sampling and laboratory procedures, documented quality-control workflow, independently recalculated alpha-diversity indices, compositional analytical framework, and extensive diagnostic and robustness assessment. Future studies can extend this framework through repeated sampling, absolute-load measurements, and a fully documented open bioinformatic workflow.

The sample-size analyses translate plausible assumptions regarding exposure-specific effects into transparent planning benchmarks. Under the conservative scenario of 90% power, α = 0.0125, and an assumed exposure-specific partial *R*^2^ of 0.03–0.05, a future age- and sex-adjusted cross-sectional regression analysis would require approximately 275–465 analyzable participants. Allowing for 10–20% attrition, incomplete assessments, unsuccessful sequencing, or other sources of unusable observations increases the corresponding enrollment target to 306–582 participants. Three planned stool collections would generate an estimated operational workload of 918–1746 specimens, whereas four collections would generate 1224–2328 specimens. These values are assumption-based cross-sectional regression benchmarks rather than universal requirements or formal sample-size estimates for a longitudinal or community-composition analysis. The exploratory calculations based on the individual effect estimates observed in the pilot regression models ranged from tens to tens of thousands of participants, with one near-zero estimate producing a clearly non-informative requirement exceeding two million. This dispersion illustrates the instability of effect-size estimates obtained from a sample of 20 participants and explains why the principal planning range was based on assumed partial *R*^2^ values rather than on the most favorable pilot estimate. A definitive protocol should prespecify the primary microbiome outcome, the minimum effect considered scientifically relevant, the multiplicity strategy, and the intended statistical model. If overall community composition is selected as the primary outcome, sample-size planning should be performed separately using a dedicated simulation-based approach that incorporates the intended distance measure, multivariate covariance and dispersion structures, exposure distribution, covariates, permutation strategy, and repeated-measures design [43].

A longitudinal extension should separate stable between-person differences from within-person change by repeating both exposure and microbiome measurements. In a cohort of 444 adults with up to three stool collections over one year, temporal variability assessed in 214 participants with repeated data showed that only 39% of 234 genera had an intraclass correlation coefficient above 0.5, while dietary-pattern associations were concentrated in a subset of genera [44]. At a broader scale, the Human Phenotype Project demonstrated longitudinal tracking of predicted and observed diet-related microbial abundances over follow-up periods of up to four years [37], while Food & You showed the additional information provided by temporally resolved dietary records [38]. Together with the short-term physiological variability documented by daily sampling [45], these findings indicate that stability, exposure window, and optimal sampling frequency are feature-specific. The three- and four-collection scenarios evaluated here are therefore practical workload models, not assumptions that repeated sampling will automatically reduce the required participant count. A definitive study should align repeated stool collections with multiple 24-h recalls, prospective food records, or another validated quantitative dietary instrument; incorporate accelerometry and actigraphy; prospectively record stool consistency, bowel frequency or transit, anthropometry, medication and supplement exposure, and recent illness; and analyse the data using prespecified mixed-effects models that account for temporal correlation, intermittent missingness, and attrition. Independent validation should be planned for the limited set of hypotheses carried forward.

Accordingly, the central contribution of this pilot is not a claim of no association. It is the construction and empirical testing of an integrated workflow that identifies where apparent signals are uncertain or participant-dependent, demonstrates which conclusions remain stable across analytical specifications, and quantifies the recruitment and laboratory workload implied by plausible effects. The UPF–*Akkermansia*, physical-activity–*Prevotella*, and physical-activity–*Prevotella*/*Bacteroides* estimates may be used to prioritize a small number of prespecified hypotheses, but they should not be interpreted as confirmed relationships. The study thus provides a transparent bridge from exploratory cross-sectional evidence to an adequately powered, temporally resolved, and outcome-specific longitudinal investigation.

## 5. Conclusions

In this cross-sectional pilot study of 20 healthy adults, Mediterranean diet adherence, UPF consumption frequency, physical activity, and sleep characteristics were not statistically associated, after correction for multiple testing, with alpha diversity, overall community composition, the CLR coordinates of eight prespecified genera, or the continuous *Prevotella*/*Bacteroides* log-ratio. Several comparatively prominent effect estimates were observed, but their uncertainty, sensitivity to individual participants, and multiplicity-adjusted results preclude confirmatory interpretation. The findings therefore should not be interpreted as evidence that the evaluated exposures are unrelated to the gut microbiota.

The principal value of the study is methodological and prospective. It establishes an integrated workflow encompassing standardized specimen handling, compositional microbiome analysis, effect-size estimation, multiplicity control, influence diagnostics, robustness analyses, and scenario-based sample-size planning. Under a conservative assumption-based scenario, 275–465 analyzable participants constitute an initial cross-sectional regression benchmark for detecting modest exposure-specific associations, corresponding to 306–582 enrolled participants after allowing for 10–20% unusable observations. These figures are neither definitive sample-size requirements nor formal power estimates for a longitudinal or community-composition analysis and must be refined according to the prespecified primary outcome, minimum scientifically relevant effect, statistical model, and repeated-measures structure of the future study. A definitive longitudinal investigation should combine a larger cohort with repeated and temporally aligned stool and exposure assessments, more detailed dietary measurement, objective monitoring of physical activity and sleep, prospective measurement of major host and intestinal covariates, and microbiome outcomes selected before analysis. In this way, the present pilot provides an empirical and analytical foundation for distinguishing reproducible biological relationships from temporal, interindividual, and methodological variability.

## Figures and Tables

**Table 1:** Participant characteristics and dietary and lifestyle exposures (n = 20).

Characteristic	Mean ± SD	Median [IQR]	Range or n (%)
Female			10 (50.0)
Male			10 (50.0)
Age, years	28.8 ± 4.5	28.0 [26.0–31.3]	21–40
MEDAS score	8.25 ± 1.83	8.5 [7.0–10.0]	4–11
MEDAS adherence category			
Low			2 (10.0)
Intermediate			12 (60.0)
High			6 (30.0)
UPF score	7.25 ± 3.73	6.5 [5.0–8.5]	2–18
UPF-consumption category			
Low			15 (75.0)
Intermediate			5 (25.0)
High			0 (0.0)
Physical activity, MET-min/week	2524.0 ± 2007.7	1902.5 [1171.3–4255.5]	0–6444
IPAQ-SF physical-activity category			
Low			5 (25.0)
Moderate			6 (30.0)
High			9 (45.0)
Adapted sleep score	5.15 ± 3.34	5.0 [2.8–7.0]	0–13
Adapted sleep-score range			
0–4			7 (35.0)
5–9			11 (55.0)
10–14			2 (10.0)
15–21			0 (0.0)

Values are presented as mean ± standard deviation (SD), median [interquartile range (IQR)], range, or n (%), as indicated. For IPAQ-SF scoring, reported activity durations exceeding 180 min/day were truncated to 180 min/day before calculating total MET-min/week. MEDAS and IPAQ-SF categories were applied according to their respective scoring frameworks. The UPF categories and adapted sleep-score ranges were study-specific operational groupings used exclusively for descriptive purposes; continuous scores were used in the association analyses. The study-specific UPF score measures reported consumption frequency and does not quantify ultra-processed food intake by weight, portion size, energy contribution, or percentage of total dietary intake. The adapted sleep score was derived from seven components informed by the principal domains of the PSQI but does not correspond to administration of the complete validated PSQI. Neither the adapted sleep score nor its descriptive score ranges should be interpreted as validated clinical or diagnostic measures. Higher MEDAS scores indicate greater Mediterranean Diet adherence; higher UPF scores indicate more frequent consumption of the selected ultra-processed food groups; higher IPAQ-SF values indicate greater physical-activity volume; and higher adapted sleep scores indicate greater reported sleep difficulty. MEDAS, Mediterranean Diet Adherence Screener; UPF, ultra-processed food; IPAQ-SF, International Physical Activity Questionnaire–Short Form; MET, metabolic equivalent of task; PSQI, Pittsburgh Sleep Quality Index.

**Table 2:** Descriptive alpha-diversity metrics and beta-diversity ordination summary in the 20 participants: (**A**) independently recalculated OTU-level within-sample diversity and evenness; and (**B**) variation represented by the first two PCoA axes for Bray–Curtis and Aitchison distances.

**Panel (A) Alpha-Diversity Metrics**			
**Metric**	**Mean ± SD**	**Median [IQR]**	**Range**
Shannon index	3.99 ± 0.27	4.09 [3.93–4.15]	3.13–4.27
Pielou’s evenness	0.647 ± 0.025	0.652 [0.639–0.660]	0.571–0.695
**Panel (B) Variation represented by the PCoA axes**			
**Distance measure**	**PCoA1 (%)**	**PCoA2 (%)**	**Cumulative variation (%)**
Bray–Curtis	25.06	23.87	48.93
Aitchison	15.91	10.26	26.17

Values in Panel A are presented as mean ± standard deviation (SD), median [interquartile range (IQR)], and range. Shannon diversity and Pielou’s evenness were independently recalculated at the OTU level from the exported count table using natural logarithms. Full-precision values were used in the statistical analyses, whereas rounded values are displayed in the table. The Shannon index was designated as the primary microbiome outcome, and Pielou’s evenness was considered a complementary alpha-diversity measure. Bray–Curtis dissimilarities were calculated from observed genus-level relative abundances, whereas Aitchison distances were calculated as Euclidean distances between centered log-ratio-transformed genus-level compositions after adding a proportional pseudocount of 1 × 10^−6^. PCoA values indicate the per-centage of total variation represented by each axis; cumulative variation is the sum of the percentages re-presented by PCoA1 and PCoA2. IQR, interquartile range; OTU, operational taxonomic unit; PCoA, principal coordinates analysis; SD, standard deviation.

**Table 3:** Descriptive profile of the eight prespecified genera and the *Prevotella*/*Bacteroides* compositional gradient in the 20 participants: (**A**) genus-level relative abundance and prevalence; (**B**) distribution of the raw and natural-log-transformed ratio; and (**C**) descriptive genus predominance.

**Panel (A) Relative Abundances of the Prespecified Genera**								
**Genus**	**Mean ± SD, %**		**Median [IQR], %**		**Range, %**		**Detected, n (%)**	
*Bacteroides*	15.34 ± 7.61		15.06 [9.77–20.85]		2.87–30.34		20 (100)	
*Faecalibacterium*	7.69 ± 3.24		8.16 [5.19–10.52]		0.93–14.29		20 (100)	
*Bifidobacterium*	3.56 ± 2.72		2.68 [1.46–5.75]		0.08–8.71		20 (100)	
*Blautia*	3.50 ± 2.08		2.62 [2.13–4.38]		1.07–8.64		20 (100)	
*Prevotella*	3.24 ± 6.56		0.07 [0–2.37]		0–22.72		15 (75)	
*Roseburia*	1.25 ± 1.17		0.97 [0.46–1.79]		<0.01–4.86		20 (100)	
*Ruminococcus*	1.25 ± 0.99		1.19 [0.59–1.68]		<0.01–3.94		20 (100)	
*Akkermansia*	0.58 ± 1.01		0.09 [0.01–0.83]		<0.01–3.60		20 (100)	
**Panel (B) Distribution of the *Prevotella*/*Bacteroides* ratio**								
**Measure**	**N**	**Mean**	**SD**	**Median**	**Q1**	**Q3**	**Minimum**	**Maximum**
Raw *Prevotella*/*Bacteroides* ratio	20	0.399	0.791	0.00454	0.000060	0.191	0	2.60
Natural-log-transformed *Prevotella*/*Bacteroides* ratio	20	−5.253	4.164	−6.342	−8.614	−1.726	−10.396	0.955
**Panel (C) Descriptive predominance categories**								
**Category**				**n (%)**				
Relative *Bacteroides* predominance				17 (85.0)				
Relative *Prevotella* predominance				3 (15.0)				
Equal observed abundances				0 (0.0)				

Panel A reports relative abundances as percentages; detection was defined as an observed abundance > 0, and <0.01 indicates a positive abundance below 0.01%. In Panel B, the raw ratio was calculated as *Prevotella*/*Bacteroides*, whereas the transformed ratio was calculated as $\ln\left[\frac{Prevotella+(c_{\left\{PB\right\})}}{Bacteroides+(c_{\left\{PB\right\})}}\right]$, with $c_{\left\{PB\right\}}=8.262619\times 10^{-6}$, corresponding to one-half of the smallest positive abundance observed across the two genera. In Panel C, predominance was assigned to the genus with the higher observed abundance; these descriptive categories do not represent formal enterotypes. IQR, interquartile range; Q1, first quartile; Q3, third quartile; SD, standard deviation.

**Table 4:** Age- and sex-adjusted associations of dietary and lifestyle exposures with independently re-calculated OTU-level alpha-diversity outcomes (n = 20).

Outcome	Exposure	β	SE	95% CI	*p*-Value	*p*-FDR	Partial *R*^2^
Shannon index	MEDAS	−0.02213	0.03410	−0.09442 to 0.05016	0.526	0.684	0.026
	UPF score	0.00701	0.01693	−0.02889 to 0.04291	0.684	0.684	0.011
	IPAQ-SF, MET-min/week	−5.82 × 10^−5^	3.61 × 10^−5^	−1.35 × 10^−4^ to 1.84 × 10^−5^	0.127	0.684	0.140
	Adapted Sleep score	0.01276	0.01959	−0.02877 to 0.05428	0.524	0.684	0.026
Pielou’s evenness	MEDAS	−0.003130	0.003149	−0.009806 to 0.003545	0.335	0.684	0.058
	UPF score	0.000743	0.001588	−0.002624 to 0.004110	0.646	0.684	0.014
	IPAQ-SF, MET-min/week	−3.24 × 10^−6^	3.56 × 10^−6^	−1.08 × 10^−5^ to 4.31 × 10^−6^	0.376	0.684	0.049
	Adapted Sleep score	0.002197	0.001781	−0.001579 to 0.005973	0.235	0.684	0.087

Each row represents a separate ordinary least-squares linear regression model adjusted for age and sex, with female as the reference category. Alpha-diversity outcomes were independently recalculated at the OTU level, and full-precision values were used in all models. β is the unstandardized coefficient associated with a one-unit increase in the exposure; the IPAQ-SF coefficient therefore represents a one-MET-min/week increase. SE and 95% CI are based on conventional model-based standard errors. Partial *R*^2^ quantifies the exposure-specific proportion of residual outcome variation explained after accounting for age and sex. The Benjamini–Hochberg procedure was applied jointly to the eight exposure coefficients in the primary alpha-diversity family. The IPAQ-SF score used in these models was calculated after truncating individual activity durations exceeding 180 min/day. CI, confidence interval; FDR, false discovery rate; IPAQ-SF, International Physical Activity Questionnaire–Short Form; MEDAS, Mediterranean Diet Adherence Screener; MET, metabolic equivalent of task; OTU, operational taxonomic unit; SE, standard error; UPF, ultra-processed food.

**Table 5:** Age- and sex-adjusted associations of dietary and lifestyle exposures with overall gut microbiota composition in the 20 participants.

Distance Measure	Exposure	Marginal *R*^2^	Pseudo-F	*p*-Value	*p*-FDR
Bray–Curtis	MEDAS	0.0348	0.646	0.784	0.800
	UPF score	0.0435	0.815	0.560	0.800
	IPAQ-SF, MET-min/week	0.0649	1.246	0.247	0.800
	Adapted Sleep score	0.0342	0.633	0.800	0.800
Aitchison	MEDAS	0.0454	0.850	0.741	0.800
	UPF score	0.0471	0.885	0.654	0.800
	IPAQ-SF, MET-min/week	0.0477	0.897	0.646	0.800
	Adapted Sleep score	0.0585	1.114	0.275	0.800

Each row represents a separate PERMANOVA model including the continuous exposure, age, and sex. Marginal *R*^2^ represents the proportion of total community variation attributable to the exposure after accounting for age and sex and should not be interpreted as conventional partial *R*^2^. Pseudo-F statistics and *p*-values refer to the marginal exposure effect and were obtained using a common matrix of 9999 unrestricted permutations. The Benjamini–Hochberg procedure was applied jointly to the eight exposure effects in the primary beta-diversity family. Bray–Curtis dissimilarities were calculated from the complete genus-level relative-abundance matrix. Aitchison distances were calculated as Euclidean distances between centered log-ratio-transformed compositions after addition of a pseudocount of 1 × 10^−6^. The IPAQ-SF score was calculated after truncating activity durations exceeding 180 min/day. FDR, false discovery rate; IPAQ-SF, International Physical Activity Questionnaire–Short Form; MEDAS, Mediterranean Diet Adherence Screener; MET, metabolic equivalent of task; UPF, ultra-processed food.

**Table 6:** Age- and sex-adjusted associations of dietary and lifestyle exposures with the centered log-ratio coordinates of the eight prespecified bacterial genera in the 20 participants.

Genus	Exposure	β	SE	95% CI	*p*-Value	*p*-FDR	Partial *R*^2^
*Bacteroides*	MEDAS	−0.04442	0.08821	−0.23142 to 0.14257	0.621	0.878	0.016
	UPF score	0.02991	0.04317	−0.06161 to 0.12143	0.498	0.867	0.029
	IPAQ-SF, MET-min/week	7.97 × 10^−5^	9.82 × 10^−5^	−1.28 × 10^−4^ to 2.88 × 10^−4^	0.429	0.867	0.040
	Adapted Sleep score	0.01219	0.05098	−0.09588 to 0.12026	0.814	0.965	0.004
*Prevotella*	MEDAS	−0.05023	0.60844	−1.34006 to 1.23960	0.935	0.982	<0.001
	UPF score	0.12777	0.29822	−0.50442 to 0.75996	0.674	0.899	0.011
	IPAQ-SF, MET-min/week	−8.98 × 10^−4^	6.48 × 10^−4^	−2.27 × 10^−3^ to 4.75 × 10^−4^	0.185	0.815	0.107
	Adapted Sleep score	−0.12943	0.34808	−0.86732 to 0.60847	0.715	0.915	0.009
*Faecalibacterium*	MEDAS	−0.10859	0.07909	−0.27624 to 0.05907	0.189	0.815	0.105
	UPF score	0.05612	0.03875	−0.02602 to 0.13826	0.167	0.815	0.116
	IPAQ-SF, MET-min/week	−5.84 × 10^−6^	9.42 × 10^−5^	−2.06 × 10^−4^ to 1.94 × 10^−4^	0.951	0.982	<0.001
	Adapted Sleep score	0.03713	0.04713	−0.06278 to 0.13703	0.442	0.867	0.037
*Bifidobacterium*	MEDAS	0.02594	0.15752	−0.30799 to 0.35987	0.871	0.982	0.002
	UPF score	−0.05108	0.07664	−0.21355 to 0.11139	0.515	0.867	0.027
	IPAQ-SF, MET-min/week	8.96 × 10^−5^	1.76 × 10^−4^	−2.84 × 10^−4^ to 4.63 × 10^−4^	0.618	0.878	0.016
	Adapted Sleep score	−0.10763	0.08647	−0.29095 to 0.07568	0.231	0.815	0.088
*Blautia*	MEDAS	0.00087	0.11517	−0.24328 to 0.24502	0.994	0.994	<0.001
	UPF score	0.03318	0.05615	−0.08586 to 0.15221	0.563	0.878	0.021
	IPAQ-SF, MET-min/week	1.55 × 10^−4^	1.24 × 10^−4^	−1.07 × 10^−4^ to 4.18 × 10^−4^	0.228	0.815	0.089
	Adapted Sleep score	0.00482	0.06615	−0.13541 to 0.14504	0.943	0.982	<0.001
*Roseburia*	MEDAS	−0.16241	0.21559	−0.61944 to 0.29462	0.462	0.867	0.034
	UPF score	0.16868	0.09955	−0.04237 to 0.37972	0.110	0.815	0.152
	IPAQ-SF, MET-min/week	−1.82 × 10^−4^	2.43 × 10^−4^	−6.97 × 10^−4^ to 3.33 × 10^−4^	0.465	0.867	0.034
	Adapted Sleep score	0.11992	0.12240	−0.13955 to 0.37940	0.342	0.867	0.057
*Ruminococcus*	MEDAS	−0.06086	0.23111	−0.55079 to 0.42907	0.796	0.965	0.004
	UPF score	0.08351	0.11222	−0.15438 to 0.32141	0.468	0.867	0.033
	IPAQ-SF, MET-min/week	−2.81 × 10^−4^	2.51 × 10^−4^	−8.14 × 10^−4^ to 2.52 × 10^−4^	0.280	0.815	0.072
	Adapted Sleep score	0.06469	0.13206	−0.21526 to 0.34464	0.631	0.878	0.015
*Akkermansia*	MEDAS	0.39241	0.33388	−0.31538 to 1.10020	0.257	0.815	0.079
	UPF score	−0.31447	0.15242	−0.63758 to 0.00865	0.056	0.815	0.210
	IPAQ-SF, MET-min/week	4.94 × 10^−4^	3.72 × 10^−4^	−2.95 × 10^−4^ to 1.28 × 10^−3^	0.203	0.815	0.099
	Adapted Sleep score	0.25279	0.18965	−0.14924 to 0.65482	0.201	0.815	0.100

Each row represents a separate ordinary least-squares linear regression model adjusted for age and sex, with female as the reference category. The outcome was the CLR coordinate of the indicated genus within the complete 241-feature genus-level composition, calculated after addition of a pseudocount of 1 × 10^−6^. β is the unstandardized coefficient associated with a one-unit increase in the exposure; IPAQ-SF coefficients therefore represent a one-MET-min/week increase. A positive coefficient indicates an increase, and a negative coefficient a decrease, in the abundance of the indicated genus relative to the geometric mean abundance of the complete genus-level composition. Coefficients do not represent changes in absolute bacterial abundance. SE and 95% CI are based on conventional model-based standard errors. Partial *R*^2^ quantifies the exposure-specific proportion of outcome variance conditional on age and sex. The Benjamini–Hochberg procedure was applied jointly to the 32 exposure–genus comparisons. CI, confidence interval; CLR, centered log-ratio; FDR, false discovery rate; IPAQ-SF, International Physical Activity Questionnaire–Short Form; MEDAS, Mediterranean Diet Adherence Screener; MET, metabolic equivalent of task; SE, standard error; UPF, ultra-processed food.

**Table 7:** Age- and sex-adjusted associations of dietary and lifestyle exposures with the natural-log-transformed *Prevotella*/*Bacteroides* ratio in the 20 participants.

Exposure	β	SE	95% CI	*p*-Value	*p*-FDR	Partial *R*^2^
MEDAS	0.103	0.568	−1.102 to 1.307	0.859	0.859	0.002
UPF score	0.096	0.279	−0.496 to 0.688	0.736	0.859	0.007
IPAQ-SF, MET-min/week	−8.42 × 10^−4^	6.05 × 10^−4^	−2.13 × 10^−3^ to 4.41 × 10^−4^	0.183	0.732	0.108
Adapted Sleep score	−0.178	0.324	−0.864 to 0.509	0.591	0.859	0.018

Each row represents a separate ordinary least-squares linear regression model adjusted for age and sex, with female as the reference category. The outcome was calculated as ln[(*Prevotella* + c)/(*Bacteroides* + c)], where c = 8.262619 × 10^−6^ in proportional-abundance units. β represents the unstandardized change in the log-ratio associated with a one-unit increase in the exposure; the IPAQ-SF coefficient therefore represents a one-MET-min/week increase. Positive coefficients indicate a higher relative balance of *Prevotella* to *Bacteroides*, whereas negative coefficients indicate a lower relative balance. SE and 95% CI are based on conventional model-based standard errors. Partial *R*^2^ quantifies the exposure-specific proportion of outcome variance conditional on age and sex. The Benjamini–Hochberg procedure was applied jointly to the four exposure–log-ratio comparisons. CI, confidence interval; FDR, false discovery rate; IPAQ-SF, International Physical Activity Questionnaire–Short Form; MEDAS, Mediterranean Diet Adherence Screener; MET, metabolic equivalent of task; SE, standard error; UPF, ultra-processed food.

**Table 8:** Scenario-based numbers of analyzable participants required for future age- and sex-adjusted cross-sectional linear regression analyses.

Partial *R*^2^	Cohen’s f^2^	α = 0.05, 80% Power	α = 0.05, 90% Power	α = 0.0125, 80% Power	α = 0.0125, 90% Power
0.01	0.0101	779	1043	1108	1418
0.03	0.0309	256	342	364	465
0.05	0.0526	152	202	216	275
0.10	0.1111	73	97	104	132

Values represent the minimum numbers of analyzable participants required for a fixed-effects multiple linear regression model testing one continuous exposure coefficient while adjusting for age and sex. Partial *R*^2^ was converted to Cohen’s f^2^ as f^2^ = partial *R*^2^/(1 − partial *R*^2^). Each model included three predictors in addition to the intercept, with one numerator degree of freedom and N − 4 denominator degrees of freedom for the exposure-specific test. Partial *R*^2^ values of 0.03 and 0.05, corresponding to Cohen’s f^2^ values of approximately 0.031 and 0.053, were designated as the principal modest-effect planning range because they represent potentially relevant exposure-specific effects without relying on the unstable estimates obtained from the small pilot sample. The α = 0.0125 scenario applies a Bonferroni-adjusted threshold accounting for the four principal exposures. Sample sizes have not been inflated for attrition, incomplete assessments, unsuccessful sequencing, or other sources of unusable observations. These estimates are cross-sectional regression benchmarks and are not formal sample-size calculations for PERMANOVA or longitudinal models.

## Data Availability

Raw 16S rRNA gene sequencing data are publicly available in the European Nucleotide Archive (ENA) under study accession PRJEB107991 (https://www.ebi.ac.uk/ena/browser/view/PRJEB107991, accessed on 2 October 2026). The custom R scripts used for downstream data processing, statistical analysis, robustness assessment, sample-size planning, and visualization are publicly available on Zenodo at https://doi.org/10.5281/zenodo.22141279. Processed, de-identified data supporting the findings of this study, including questionnaire-derived exposure data, microbiome outcomes, sequencing quality-control metrics, and extended statistical results, are provided in the article and its Supplementary Materials.

## Supplementary Materials

The following supporting information can be downloaded at: https://www.mdpi.com/article/10.3390/nu18203325/s1, Supplementary Questionnaire S1, Eligibility Screening Questionnaire for the Source Cohort; Supplementary Questionnaire S2, Dietary and Lifestyle Data-Collection Instrument; Supplementary Table S1, detailed laboratory, sequencing, primary bioinformatic, and alpha-diversity processing workflow used for the fresh-stool microbiome profiles; Supplementary Table S2, scoring, derivation, and operational treatment of dietary and lifestyle exposures; Supplementary Table S3, participant-level demographic characteristics, exposure scores, and descriptive classifications; Supplementary Table S4A, participant-level responses to MEDAS items 1–7; Supplementary Table S4B, participant-level responses to MEDAS items 8–14, total scores, and adherence categories; Supplementary Table S5, participant-level responses to the nine UPF items, total scores, and operational consumption categories; Supplementary Table S6, participant-level IPAQ-SF responses, corrected MET-min/week values, and physical-activity categories; Supplementary Table S7A, participant-level sleep timing, latency, duration, and subjective sleep quality; Supplementary Table S7B, participant-level frequency of sleep-related disturbances; Supplementary Table S7C, participant-level sleep-medication use and daytime consequences; Supplementary Table S8, participant-level adapted sleep-component scores, total scores, and descriptive score ranges; Supplementary Table S9, sequencing performance, quality-control metrics, and OTU-table characteristics for the 20 fresh-stool microbiome profiles; Supplementary Table S10, descriptive statistics for the ten most abundant genus-level taxonomic groups in the 20 fresh-stool microbiome profiles; Supplementary Table S11, participant-level classified OTU-read depth, observed OTU richness, and independently recalculated full-precision alpha-diversity values; Supplementary Table S12, participant-level coordinates from the Bray–Curtis and Aitchison principal coordinates analyses; Supplementary Table S13, participant-level relative abundances of the eight prespecified bacterial genera; Supplementary Table S14, participant-level *Prevotella* and *Bacteroides* abundances, ratios, and descriptive predominance categories; Supplementary Table S15, model fit, diagnostic assessment, and robustness analyses for associations of dietary and lifestyle exposures with independently recalculated OTU-level alpha diversity: (A) model fit and exposure-specific effect size; (B) regression diagnostics and leave-one-out influence analysis; (C) HC3 and Freedman–Lane permutation inference; (D) sensitivity to the original untruncated IPAQ-SF score; (E) sensitivity after repeated rarefaction to 3026 classified OTU reads; and (F) sensitivity after exclusion of the lowest-depth participant; Supplementary Table S16, PERMANOVA model results, multivariate-dispersion diagnostics, and robustness analyses for associations of dietary and lifestyle exposures with overall gut microbiota composition in the 20 participants: (A) complete-model results for the primary analyses; (B) multivariate-dispersion diagnostics; (C) sensitivity analyses using prevalence-filtered genus-level compositions; (D) Aitchison-distance sensitivity analyses using alternative pseudocounts; and (E) sensitivity analyses using the original untruncated IPAQ-SF score; Supplementary Table S17, diagnostic assessment and robustness analyses for associations of dietary and lifestyle exposures with the CLR coordinates of the eight prespecified bacterial genera in the 20 participants: (A) comparison of conventional, HC3, and Freedman–Lane inference; (B) regression-diagnostic and leave-one-out summary; (C) sensitivity to alternative abundance representations; (D) sensitivity to alternative CLR pseudocounts; and (E) sensitivity to use of the original untruncated IPAQ-SF score; Supplementary Table S18, model fit, diagnostic assessment, influence analyses, and robustness of associations between dietary and lifestyle exposures and the natural-log-transformed *Prevotella*/*Bacteroides* ratio: (A) model fit and regression diagnostics; (B) leave-one-out analyses; (C) HC3 and Freedman–Lane inference; (D) sensitivity to IPAQ-SF truncation; and (E) temporary exclusion of the lowest-depth microbiome profile; Supplementary Table S19, enrollment targets and stool-specimen workloads across prespecified effect-size, power, significance-threshold, and data-loss scenarios; Supplementary Table S20, exploratory model-specific sample-size benchmarks derived from pilot exposure-specific effect estimates for alpha diversity, CLR-transformed prespecified genera, and the natural-log-transformed *Prevotella*/*Bacteroides* ratio; Supplementary Figure S1, participant-level sequencing yield and taxonomic-classification performance; Supplementary Figure S2, participant-level distribution of the eight prespecified bacterial genera; Supplementary Figure S3, direction and magnitude of age- and sex-adjusted associations between dietary and lifestyle exposures and the centered log-ratio coordinates of the eight prespecified bacterial genera; Supplementary Figure S4, model-specific sample-size benchmarks derived from exposure-specific partial R^2^ values observed in the pilot linear-regression analyses; and Supplementary Figure S5, sequencing-depth diagnostics for independently recalculated OTU-level alpha diversity.

## Abbreviations

The following abbreviations are used in this manuscript:

16S rRNA	16S ribosomal ribonucleic acid
CET-CEROM	Comitato Etico della Romagna
CI	Confidence interval
CLR	Centered log-ratio
DNA	Deoxyribonucleic acid
ENA	European Nucleotide Archive
FDR	False discovery rate
HC3	Heteroscedasticity-consistent type 3
IPAQ-SF	International Physical Activity Questionnaire–Short Form
IQR	Interquartile range
MEDAS	Mediterranean Diet Adherence Screener
MET	Metabolic equivalent of task
P/B	*Prevotella*/*Bacteroides*
PCoA	Principal coordinates analysis
PCR	Polymerase chain reaction
PERMANOVA	Permutational multivariate analysis of variance
PREDIMED	Prevención con Dieta Mediterránea
PSQI	Pittsburgh Sleep Quality Index
Q1	First quartile
Q3	Third quartile
RDP	Ribosomal Database Project
SD	Standard deviation
SE	Standard error
STORMS	Strengthening the Organization and Reporting of Microbiome Studies
STROBE	Strengthening the Reporting of Observational Studies in Epidemiology
UPF	Ultra-processed food
