# Supplementary material for: Dietary and Lifestyle Correlates of Gut Microbiota Diversity and Composition in Healthy Adults: A Cross-Sectional Pilot Study Informing Longitudinal Research
Source: Nutrients. 2026 Oct 10;18(20):3325. doi: 10.3390/nu18203325 (PMC13708450; doi:10.3390/nu18203325)
Supplement: Supplementary file 1 [file nutrients-18-03325-s001.zip › Supplementary_Questionnaire_S1_Eligibility_Screening_corrected.pdf]

Supplementary Questionnaire S1

Eligibility Screening Questionnaire for the Source Cohort

Study-specific screening instrument used to assess eligibility for the cohort from which the present analytical sample was derived.

Participant study ID	____		
Age (years)	________________	Sex	Female [ ] Male [ ]
Date of stool collection	____		

Eligibility Screening

Please mark one response for each item. Participants were considered eligible only if they answered Yes to item 1, No to items 2-14, and No to item 15 when applicable.

No.	Question	Yes	No
1	Are you between 20 and 40 years of age?	[ ]	[ ]
2	Do you currently have, or have you ever been diagnosed with, any chronic medical condition (e.g., diabetes, hypertension, cardiovascular disease, chronic liver disease, chronic kidney disease, cancer, or another long-term systemic disease)?	[ ]	[ ]
3	Are you currently taking any medication on a regular basis?	[ ]	[ ]
4	Have you taken antibiotics within the last 6 months?	[ ]	[ ]
5	Have you taken probiotics, prebiotics, synbiotics, or other microbiota-modulating supplements within the last 4 weeks?	[ ]	[ ]
6	Have you used laxatives, antidiarrheal drugs, proton-pump inhibitors, or bowel-cleansing preparations within the last 4 weeks?	[ ]	[ ]
7	Have you had any gastrointestinal infection within the last 6 months (e.g., *Clostridioides difficile*, *Salmonella*, *Campylobacter*, viral gastroenteritis, or other infectious diarrhea)?	[ ]	[ ]
8	Have you ever been diagnosed with an autoimmune disease?	[ ]	[ ]

Eligibility Screening (continued)

No.	Question	Yes	No
9	Have you ever been diagnosed with inflammatory bowel disease (e.g., Crohn's disease or ulcerative colitis)?	[ ]	[ ]
10	Have you ever been diagnosed with irritable bowel syndrome (IBS)?	[ ]	[ ]
11	Have you undergone any major gastrointestinal surgery?	[ ]	[ ]
12	Have you undergone colonoscopy or bowel preparation within the last 3 months?	[ ]	[ ]
13	Do you currently have diarrhea, vomiting, fever, or any other acute gastrointestinal symptoms or illness?	[ ]	[ ]
14	Have you made any major dietary change within the last 4 weeks (e.g., initiation of a restrictive diet, major weight-loss diet, ketogenic diet, vegan diet, elemental diet, or medically prescribed exclusion diet)?	[ ]	[ ]
15	For female participants only: are you currently pregnant?	[ ]	[ ]

Eligibility rule: Yes to item 1; No to items 2-14; and No to item 15 when applicable.

Note: This questionnaire was developed for recruitment of the source cohort and is reproduced here as supplementary material for the present study. No participant responses are shown.

Abbreviation: IBS, irritable bowel syndrome.
