# Supplementary material for: Dietary and Lifestyle Correlates of Gut Microbiota Diversity and Composition in Healthy Adults: A Cross-Sectional Pilot Study Informing Longitudinal Research
Source: Nutrients. 2026 Oct 10;18(20):3325. doi: 10.3390/nu18203325 (PMC13708450; doi:10.3390/nu18203325)
Supplement: Supplementary file 1 [file nutrients-18-03325-s001.zip › Supplementary_Questionnaire_S2_Dietary_Lifestyle.pdf]

Supplementary Questionnaire S2

Dietary and Lifestyle Data-Collection Instrument

English translation of the Italian-language instrument administered to participants. No participant responses are shown. The UPF and adapted sleep sections are study-specific exploratory measures; the sleep section is not the complete official PSQI.

Participant ID	____________________________	Completion date	____ / ____ / ______
Age (years)	____________	Sex	Female [ ] Male [ ]

General instructions

Report your usual behavior. Unless otherwise stated, questions refer to the preceding month; physical-activity questions refer to the preceding 7 days. Mark one response per item unless instructed otherwise.

Section 1 - Mediterranean Diet (MEDAS)

Mark Yes if the described behavior reflects your usual habits; otherwise mark No.

No.	Question	Yes	No
1	Do you use extra-virgin olive oil as the main culinary fat?	[ ]	[ ]
2	Do you consume at least 4 tablespoons of extra-virgin olive oil per day, including oil used for cooking, salads, and meals eaten away from home?	[ ]	[ ]
3	Do you consume at least 2 servings of vegetables per day?	[ ]	[ ]
4	Do you consume at least 3 servings of fruit per day?	[ ]	[ ]
5	Do you consume less than 1 serving of red or processed meat per day?	[ ]	[ ]
6	Do you consume less than 1 serving of butter, margarine, or cream per day?	[ ]	[ ]
7	Do you consume fewer than 1 sugar-sweetened beverage per day?	[ ]	[ ]
8	Do you consume at least 7 glasses of wine per week?	[ ]	[ ]

Section 1 - Mediterranean Diet (continued)

No.	Question	Yes	No
9	Do you consume at least 3 servings of legumes per week?	[ ]	[ ]
10	Do you consume at least 3 servings of fish or seafood per week?	[ ]	[ ]
11	Do you consume commercial sweets, industrial baked goods, or packaged desserts on fewer than 3 occasions per week?	[ ]	[ ]
12	Do you consume at least 3 servings of nuts per week?	[ ]	[ ]
13	Do you prefer white meat (chicken, turkey, or rabbit) to red or processed meat?	[ ]	[ ]
14	Do you consume a tomato, onion, garlic, or leek sofrito prepared with extra-virgin olive oil at least twice per week?	[ ]	[ ]

Scoring: one point for each adherence criterion met; total range 0-14. Derived from the 14-item MEDAS developed within the PREDIMED framework.

Section 2 - Ultra-Processed Food Consumption

Report usual consumption frequency for each category: 0 = never or almost never; 1 = once per week; 2 = 2-4 times per week; 3 = at least 5 times per week.

No.	Category	0	1	2	3
1	Packaged savory snacks (e.g., potato chips, pretzels, industrial crackers)	[ ]	[ ]	[ ]	[ ]
2	Sugar-sweetened beverages (e.g., cola, orange soda, sweetened iced tea)	[ ]	[ ]	[ ]	[ ]
3	Industrially produced sweets (e.g., snack cakes, filled biscuits, packaged sweet snacks)	[ ]	[ ]	[ ]	[ ]
4	Fast food or industrial quick-service meals	[ ]	[ ]	[ ]	[ ]
5	Ready-to-eat or frozen meals requiring reheating	[ ]	[ ]	[ ]	[ ]
6	Processed meats (e.g., frankfurters, nuggets, industrial cold cuts)	[ ]	[ ]	[ ]	[ ]
7	Sugar-sweetened breakfast cereals	[ ]	[ ]	[ ]	[ ]
8	Industrially produced sauces (e.g., mayonnaise, ketchup, ready-made dressings)	[ ]	[ ]	[ ]	[ ]
9	Industrially produced energy or functional beverages	[ ]	[ ]	[ ]	[ ]

Study-specific exploratory score informed by the NOVA classification; item scores are summed to obtain a total ranging from 0 to 27.

Section 3 - Physical Activity (IPAQ-SF)

The following questions refer to the preceding 7 days. Include only activities performed for at least 10 consecutive minutes.

Activity type	Days/week	Minutes/day
Vigorous activity (e.g., running, intense sport, or exercise that makes you breathe much harder than normal)	________	________
Moderate activity (e.g., recreational cycling, light gym exercise, or activity that makes you breathe somewhat harder than normal)	________	________
Walking (e.g., transport, leisure walking, or usual journeys)	________	________

Time spent sitting on a typical weekday (minutes/day): ____________________

The IPAQ-SF MET-min/week score and descriptive activity category were derived after data collection; sitting time was not included in the total score.

Section 3b - Additional Physical-Activity Information (exploratory)

These additional questions were collected for descriptive purposes and were not used as primary exposures in the statistical models.

Main activity: Walking [ ] Running [ ] Cycling [ ] Gym/fitness [ ] Team sport [ ] Strength training [ ] Yoga/Pilates [ ] Other [ ]

Planned exercise frequency: Never [ ] Once/week [ ] Twice/week [ ] Three times/week [ ] Four or more times/week [ ]

Average session duration: <30 min [ ] 30-45 min [ ] 46-60 min [ ] >60 min [ ]

Section 4 - Sleep Characteristics (adapted instrument)

The following questions refer to the preceding month. Complete each field according to your most frequent habits.

Question	Response
What time do you usually go to bed?	______________
How long does it usually take you to fall asleep? (minutes)	______________
What time do you usually wake up?	______________
How many hours of actual sleep do you obtain per night, on average?	______________

Overall, how would you rate your sleep quality during the preceding month?

Very good [ ] Fairly good [ ] Fairly poor [ ] Very poor [ ]

During the preceding 30 days, how often did you experience sleep disturbance for the following reasons?

Reason	Never	<1 time/week	1-2 times/week	≥3 times/week
Difficulty falling asleep within 30 minutes	[ ]	[ ]	[ ]	[ ]
Waking during the night or early in the morning	[ ]	[ ]	[ ]	[ ]
Having to get up to use the bathroom	[ ]	[ ]	[ ]	[ ]
Difficulty breathing or shortness of breath	[ ]	[ ]	[ ]	[ ]
Feeling too hot or too cold to sleep comfortably	[ ]	[ ]	[ ]	[ ]
Pain or physical discomfort interrupting sleep	[ ]	[ ]	[ ]	[ ]

Use of sleep medication during the preceding 30 days

Never [ ] <1 time/week [ ] 1-2 times/week [ ] ≥3 times/week [ ]

Difficulty staying awake during the day

Never [ ] <1 time/week [ ] 1-2 times/week [ ] ≥3 times/week [ ]

How difficult was it to maintain enthusiasm and performance in daily activities?

No difficulty [ ] Mild [ ] Moderate [ ] Severe [ ]

The seven-component adapted Sleep score was derived after data collection. This study-specific instrument incorporated principal PSQI domains but was not the complete validated PSQI and was not interpreted as a clinical or diagnostic measure.
