# Supplementary material for: Dietary and Lifestyle Correlates of Gut Microbiota Diversity and Composition in Healthy Adults: A Cross-Sectional Pilot Study Informing Longitudinal Research
Source: Nutrients. 2026 Oct 10;18(20):3325. doi: 10.3390/nu18203325 (PMC13708450; doi:10.3390/nu18203325)
Supplement: Supplementary file 1 [file nutrients-18-03325-s001.zip › V2 Supplementary material Dietary and Lifestyle Correlates of the Gut Microbiota in Healthy Adults- A Cross-Sectional Pilot Study to Inform Lon-gitudinal Research.pdf]

Supplementary material
Table

Supplementary Table S1. Detailed laboratory, sequencing, primary bioinformatic, and alpha-diversity processing workflow used for the fresh-stool microbiome profiles.

Workflow stage	Procedure and parameters
Biological input	A 250 mg aliquot of fresh stool was used for microbial DNA extraction from each participant
DNA extraction	QIAamp PowerFecal Pro DNA Kit, cat. no. 51804 (QIAGEN, Hilden, Germany), operated on a QIAcube Connect MDx automated platform (QIAGEN)
Lysis and mechanical disruption	Stool was transferred to a PowerBead Pro tube containing 800 μL of Solution CD1 and subjected to vortexing, heat treatment, and mechanical disruption using a TissueLyser II (QIAGEN). After centrifugation, 600 μL of supernatant was transferred to the QIAcube Connect MDx for automated purification
DNA quantification	Qubit 1X dsDNA High Sensitivity Assay Kit and Qubit fluorometer (Invitrogen, Thermo Fisher Scientific, Waltham, MA, USA)
DNA normalization	Extracts with concentrations >1 ng/μL were diluted with UltraPure distilled water to a target concentration of 1 ng/μL; extracts with concentrations ≤1 ng/μL were processed without further dilution
Automated library preparation	Microlab VANTAGE Liquid Handling System (Hamilton Company, Reno, NV, USA) with the Microbiota Solution B Kit, cat. no. AD-002.024 (Arrow Diagnostics, Genoa, Italy)
16S rRNA targets	V3–V4 and V6 hypervariable regions, amplified using the manufacturer-supplied primer mixture
Target-PCR master-mix components reported per sample	Molecular-grade water, 3 μL; Enzyme Mix 1, 10 μL; Amp Mix, 2 μL
Target-PCR thermal profile	Initial denaturation: 95 °C for 5 min; 25 cycles of 95 °C for 30 s, 55 °C for 30 s, and 72 °C for 30 s; final extension: 72 °C for 5 min; hold at 4 °C
Inter-stage purification	AMPure XP magnetic beads (Beckman Coulter, Brea, CA, USA)
Index-PCR master-mix components reported per sample	Molecular-grade water, 8 μL; Enzyme Mix 2, 10 μL
Index-PCR thermal profile	Initial denaturation: 95 °C for 5 min; nine cycles of 95 °C for 30 s, 65 °C for 30 s, and 72 °C for 30 s; final extension: 72 °C for 5 min; hold at 4 °C
Library quantification and normalization	Purified libraries were quantified fluorometrically. Concentrations were converted from ng/μL to nM using a nominal amplicon length of 900 bp, normalized to 10 nM, and pooled in equal volumes
Sequencing platform	Illumina MiSeq using the MiSeq Reagent Kit v3 with 2 × 300-bp paired-end sequencing and sequencing-by-synthesis chemistry (Illumina, San Diego, CA, USA)
Internal sequencing control	PhiX Control v3 added at 10% (Illumina)
Primary bioinformatic processing	MicrobAT version 1.3.0, Microbiota Analysis Tool (SmartSeq S.r.l., Novara, Italy)

Pipeline operations	Quality filtering, OTU assignment, chimera handling, taxonomic classification, software-based calculation of alpha-diversity indices, and generation of count and abundance tables at multiple taxonomic levels
Feature definition	The exported analytical profiles were OTU based. No separate external denoising or ASV-inference procedure was applied
Taxonomic assignment	Taxonomic classification was performed using the reference database embedded in MicrobAT. Assigned sequences were reported as RDP-assigned reads in the quality-control output
Pipeline settings	OTU-clustering thresholds, read-filtering cutoffs, chimera-handling parameters, classifier settings, and the reference-database version were embedded in the commercial pipeline and were neither user-configurable nor reported in the exported output
Exported OTU table	The exported count table contained 1756 rows. One software-generated aggregate row labelled "taxa1" did not represent an individual OTU and was excluded, leaving 1755 OTU rows for independent alpha-diversity recalculation
Observed OTU richness	Independently recalculated as the number of OTUs with a positive count for each participant
Shannon diversity	Independently recalculated as $H_i = -\Sigma_j p_{ij} \ln(p_{ij})$, where $p_{ij} = n_{ij}/\Sigma_j n_{ij}$; natural logarithms were used, and only OTUs with positive counts contributed to the summation
Pielou's evenness	Independently recalculated as $J_i = H_i/\ln(S_i)$, where S_i denotes the participant-specific observed OTU richness
Independent verification	Independently recalculated observed richness reproduced the MicrobAT-reported values exactly. Shannon diversity and Pielou's evenness reproduced the software output within its two-decimal reporting precision for all 20 participants. Full-precision independently recalculated values were used in the revised analyses
Primary alpha-diversity preprocessing	No rarefaction, prevalence filtering, or additional low-abundance filtering was applied before the primary alpha-diversity calculations
Genus-level analytical table	For community-composition and taxon-specific analyses, exported counts were aggregated at the genus level, producing a table containing 241 features. Formally assigned genera and incompletely classified groups retained at the most informative available taxonomic level were included
Downstream relative-abundance calculation	Genus-level relative abundance was calculated within each participant by dividing each feature count by the total count in the corresponding genus-level table
Community- and taxon-level analyses	The genus-level compositions were used to calculate Bray–Curtis dissimilarities, Aitchison distances, centered log-ratio coordinates, and the natural-log-transformed Prevotella/Bacteroides ratio
Sequencing-depth sensitivity analysis	Sequencing-depth dependence was evaluated using correlations, repeated rarefaction without replacement, and exclusion of the lowest-depth participant. For the rarefaction sensitivity analysis, each participant was rarefied 1000 times to the common minimum depth of 3026 classified OTU reads, and participant-level mean alpha-diversity values were used
Additional filtering	No additional external filtering was applied before the primary downstream analyses. Incompletely classified taxonomic groups were retained. Rarefaction was performed exclusively as a sensitivity analysis and did not replace the full-depth primary analysis
Quality-control flags	Extracted DNA concentrations <1 ng/μL and sequencing-library concentrations <5 ng/μL were treated as quality-control flags rather than automatic exclusion criteria
Extraction-failure criterion	Absence of a recoverable DNA extract or failure to generate a sequencing library

Sequencing-failure criterion	Absence of a usable MicrobAT-derived OTU profile
Analytical inclusion criterion	A profile was retained when DNA extraction and library preparation were successful, sequencing yielded nonzero reads, and MicrobAT generated an OTU profile with valid taxonomic output, irrespective of DNA or library concentration alone
Participant-sample quality-control outcome	No extraction, library-preparation, or sequencing failure was recorded among the 160 participant-derived profiles generated in the source laboratory study. All 20 fresh-stool profiles used in the present analysis satisfied the analytical inclusion criterion
Blank and negative-extraction controls	Included in each sequencing run and evaluated as part of the routine laboratory quality-assurance workflow
Blank and negative-control outcome	Routine review did not identify evidence of contamination requiring rejection of a sequencing run, repetition or exclusion of a participant sample, or removal of individual taxa
Mock-community controls	Evaluated during preliminary workflow verification but not included in the participant-level analytical dataset
Control handling	Because the blank and negative controls did not indicate contamination requiring corrective action, they were not incorporated into the participant-level analytical dataset, and no control-based contaminant-removal procedure was required
Reproducibility boundary	Upstream processing within the proprietary MicrobAT workflow could not be reproduced independently in full. All downstream transformations and statistical analyses starting from the exported OTU count table were independently implemented and documented

Footnote: Only the fresh-stool profile selected for each of the 20 participants was used in the present dietary and lifestyle analyses. The reaction-mix entries reproduce the components and volumes documented in the validated laboratory workflow; components automatically dispensed or supplied within the commercial system are not inferred beyond the available records. The proprietary MicrobAT clustering, read-filtering, chimera-handling, classification, and reference-database settings were unavailable in the exported analytical output and therefore could not be reported more specifically. The exported OTU count table nevertheless permitted independent recalculation and verification of observed richness, Shannon diversity, and Pielou's evenness and reproducible implementation of the downstream statistical workflow. Blank and negative-extraction controls were included in each sequencing run and reviewed as part of routine laboratory quality assurance. No evidence of contamination requiring run rejection, sample repetition or exclusion, or removal of individual taxa was identified; therefore, no control-based contaminant-removal procedure was required. Mock-community controls were evaluated during preliminary workflow verification but were not included in the participant-level analytical dataset. No external denoising, amplicon sequence variant inference, quantitative PCR, spike-in control, or absolute bacterial-load measurement was performed. The primary alpha-diversity analysis was conducted without rarefaction; repeated rarefaction was used exclusively as a sequencing-depth sensitivity analysis. Consequently, taxon-level results represent relative rather than absolute microbial abundance. ASV, amplicon sequence variant; OTU, operational taxonomic unit; PCR, polymerase chain reaction; RDP, Ribosomal Database Project.

Supplementary Table S2. Scoring, derivation, and operational treatment of dietary and lifestyle exposures.

Panel A. Exposure scores and descriptive classifications

Measure	Information included	Scoring or derivation	Range or categories	Analytical use
MEDAS score	Fourteen items assessing adherence to the Mediterranean dietary pattern	Each item was scored 1 when the adherence criterion was met and 0 otherwise; item scores were summed	0–14	Continuous exposure in association models
MEDAS adherence category	Total MEDAS score	Low: 0–5; intermediate: 6–9; high: 10–14	Three categories	Descriptive only
UPF score	Reported consumption frequency of nine study-specific food groups selected to represent commonly consumed and readily recognizable examples of industrially formulated foods consistent with the NOVA concept: packaged savory snacks, sugar-sweetened beverages, industrially produced sweets, fast food, ready-to-eat meals, processed meats, sugar-sweetened breakfast cereals, industrially produced sauces, and energy or functional beverages	Each item was scored 0 = never or almost never, 1 = once/week, 2 = 2–4 times/week, or 3 = ≥5 times/week. These ordered frequency levels were selected to distinguish absent or negligible, occasional, recurrent, and frequent consumption. Items were equally weighted because no validated empirical basis was available for assigning differential weights; the nine item scores were summed	0–27	Exploratory continuous exposure in association models
UPF-consumption category	Total UPF score	Low: 0–9; intermediate: 10–18; high: 19–27. These cutoffs represented pragmatic partitions of the theoretical score range and were not validated intake thresholds	Three study-specific operational categories	Descriptive only
IPAQ-SF total physical activity	Days/week and minutes/day of walking and moderate- and vigorous-intensity activity during the previous 7 days	Total MET-min/week = 3.3 × walking minutes/day × walking days/week + 4.0 × moderate-intensity minutes/day × moderate-intensity days/week + 8.0 × vigorous-intensity minutes/day × vigorous-intensity days/week. Reported durations exceeding 180 min/day for an individual activity were truncated to 180 min/day before calculation	Continuous	Continuous exposure in association models
High physical-activity category	Frequency and total volume of activity	Vigorous activity on ≥3 days and ≥1500 MET-min/week, or any combination of walking and	High	Descriptive only

		moderate- or vigorous-intensity activity on ≥7 days and ≥3000 MET-min/week		
Moderate physical-activity category	Frequency, duration, and total volume of activity	Vigorous activity on ≥3 days for ≥20 min/day; or moderate activity and/or walking on ≥5 days for ≥30 min/day; or any combination of walking and moderate- or vigorous-intensity activity on ≥5 days and ≥600 MET-min/week	Moderate	Descriptive only
Low physical-activity category	Frequency and total volume of activity	Criteria for the moderate or high categories not met	Low	Descriptive only
Adapted sleep score	Seven components covering subjective sleep quality, sleep latency, sleep duration, habitual sleep efficiency, sleep disturbances, use of sleep medication, and daytime dysfunction	Each component was scored from 0 to 3, with higher values indicating greater reported sleep difficulty; the seven component scores were summed	0–21	Exploratory continuous exposure in association models
Adapted sleep-score range	Total adapted sleep score	0–4; 5–9; 10–14; or 15–21. These ranges divided the theoretical scale into successive five-point intervals, with the final range extending to the theoretical maximum. They were not assigned qualitative or diagnostic labels and were not derived from clinical validation	Four study-specific descriptive score ranges	Descriptive only

Panel B. Scoring of the seven adapted sleep components

Component	Information used	Component scoring
C1—Subjective sleep quality	Overall self-rated sleep quality during the previous month	Very good = 0; fairly good = 1; fairly poor = 2; very poor = 3
C2—Sleep latency	Time required to fall asleep and frequency of difficulty falling asleep within 30 min	Latency: ≤15 min = 0; 16–30 min = 1; 31–60 min = 2; >60 min = 3. Difficulty frequency: never = 0; <1 time/week = 1; 1–2 times/week = 2; ≥3 times/week = 3. Sum of the two scores: 0 = component 0; 1–2 = component 1; 3–4 = component 2; 5–6 = component 3
C3—Sleep duration	Mean hours of actual sleep per night	>7 h = 0; 6–7 h = 1; 5 to <6 h = 2; <5 h = 3
C4—Habitual sleep efficiency	Actual sleep duration divided by time spent in bed and multiplied by 100	≥85% = 0; 75 to <85% = 1; 65 to <75% = 2; <65% = 3

C5—Sleep disturbances	Nocturnal or early awakening, getting up to use the bathroom, breathing difficulty, thermal discomfort, and pain or discomfort	Each frequency item was scored from 0 to 3. Sum of the five items: 0 = component 0; 1–5 = component 1; 6–10 = component 2; 11–15 = component 3
C6—Use of sleep medication	Frequency of medication use for sleep during the previous month	Never = 0; <1 time/week = 1; 1–2 times/week = 2; ≥3 times/week = 3
C7—Daytime dysfunction	Difficulty remaining awake during the day and difficulty maintaining enthusiasm or performance	Daytime sleepiness: never = 0; <1 time/week = 1; 1–2 times/week = 2; ≥3 times/week = 3. Performance difficulty: none = 0; mild = 1; moderate = 2; severe = 3. Sum of the two scores: 0 = component 0; 1–2 = component 1; 3–4 = component 2; 5–6 = component 3

Footnote: The dietary and lifestyle questionnaire was provided together with the eligibility-screening questionnaire, and participants selected for the analytical cohort were instructed to complete it on the same day that they produced the stool specimen for laboratory delivery. The nine UPF groups, item-frequency levels, equal weighting, and total-score classification were specified as components of the study-specific questionnaire and scoring procedure before the current association analyses. The UPF score was designed as a brief frequency-based indicator rather than as an exhaustive application of NOVA group 4. It has not been externally validated and does not quantify portion size, food weight, energy intake, the energy contribution of ultra-processed foods, or the proportion of the total diet represented by ultra-processed foods. Its total score and operational categories were therefore treated as exploratory. For IPAQ-SF scoring, reported durations exceeding 180 min/day for an individual activity were truncated to 180 min/day before calculating total MET-min/week. This correction affected one participant who reported 240 min/day of walking, reducing the total score from 6984 to 5598 MET-min/week without changing the high-activity classification. MEDAS and IPAQ-SF categories were applied according to their respective scoring frameworks. The adapted sleep assessment incorporated seven components informed by the principal domains of the PSQI but did not constitute administration of the complete validated PSQI. Its total score and descriptive score ranges have not been clinically validated and must not be interpreted as validated sleep-quality measures, clinically meaningful categories, or diagnostic classifications. Higher MEDAS scores indicate greater Mediterranean Diet adherence; higher UPF scores indicate more frequent consumption of the nine selected food groups; higher IPAQ-SF values indicate greater physical-activity volume; and higher adapted sleep scores indicate greater reported sleep difficulty. IPAQ-SF, International Physical Activity Questionnaire–Short Form; MEDAS, Mediterranean Diet Adherence Screener; MET, metabolic equivalent of task; PSQI, Pittsburgh Sleep Quality Index; UPF, ultra-processed food.

Supplementary Table S3. Participant-level demographic characteristics, exposure scores, and descriptive classifications.

Participant	Age	Sex	MEDAS score	MEDAS category	UPF score	UPF category	IPAQ-SF, MET-min/week	IPAQ-SF category	Adapted sleep score	Adapted sleep-score range
1M	28	M	9	Intermediate	2	Low	2100	High	0	0–4
2M	26	M	8	Intermediate	10	Intermediate	5196	High	10	10–14
3M	31	M	5	Low	4	Low	4476	High	7	5–9

4M	32	M	7	Intermediate	18	Intermediate	1939	Moderate	5	5–9
5M	29	M	10	High	4	Low	4182	High	9	5–9
6M	40	M	9	Intermediate	5	Low	297	Low	6	5–9
7M	24	M	8	Intermediate	7	Low	6444	High	5	5–9
8M	27	M	10	High	6	Low	1356	Moderate	1	0–4
9M	26	M	8	Intermediate	11	Intermediate	1866	Moderate	7	5–9
10M	27	M	9	Intermediate	5	Low	1598	Low	0	0–4
1F	27	F	6	Intermediate	11	Intermediate	2373	High	5	5–9
2F	25	F	10	High	2	Low	5652	High	4	0–4
3F	33	F	8	Intermediate	8	Low	1836	High	5	5–9
4F	22	F	10	High	11	Intermediate	297	Low	13	10–14
5F	33	F	9	Intermediate	8	Low	480	Low	2	0–4
6F	28	F	4	Low	6	Low	1836	Moderate	6	5–9
7F	31	F	7	Intermediate	6	Low	2337	Moderate	5	5–9
8F	34	F	10	High	5	Low	0	Low	3	0–4
9F	21	F	7	Intermediate	8	Low	5598	High	8	5–9
10F	31	F	11	High	8	Low	617	Moderate	2	0–4

Footnote: Participant identifiers are pseudonymized. Scores, established classifications, and study-specific operational groupings were derived according to the rules reported in Supplementary Table S2. IPAQ-SF values incorporate the 180-min/day truncation rule. MEDAS and IPAQ-SF categories were applied according to their respective scoring frameworks. UPF categories were study-specific operational classifications used exclusively for descriptive purposes. Adapted sleep-score ranges were likewise used only to describe the distribution of the study-specific score and do not represent validated sleep-quality categories, clinically meaningful thresholds, or diagnostic classifications. Continuous MEDAS, UPF, IPAQ-SF, and adapted sleep scores were used in the association analyses. F, female; IPAQ-SF, International Physical Activity Questionnaire–Short Form; M, male; MEDAS, Mediterranean Diet Adherence Screener; MET, metabolic equivalent of task; UPF, ultra-processed food.

Supplementary Table S4A. Participant-level responses to MEDAS items 1–7.

Participant	M1	M2	M3	M4	M5	M6	M7
1M	Yes	No	Yes	Yes	Yes	Yes	Yes
2M	Yes	No	Yes	Yes	Yes	Yes	Yes
3M	No	No	No	No	Yes	Yes	Yes
4M	Yes	Yes	No	No	No	Yes	No
5M	Yes	Yes	Yes	Yes	Yes	Yes	Yes
6M	Yes	No	Yes	No	Yes	Yes	Yes
7M	Yes	No	No	No	Yes	Yes	Yes
8M	Yes	Yes	Yes	No	Yes	Yes	Yes
9M	Yes	Yes	Yes	No	Yes	Yes	No
10M	Yes	Yes	Yes	No	Yes	No	Yes
1F	Yes	Yes	Yes	No	Yes	Yes	Yes
2F	Yes	Yes	Yes	Yes	Yes	Yes	Yes
3F	Yes	No	Yes	No	Yes	Yes	No
4F	Yes	Yes	Yes	No	Yes	Yes	Yes
5F	No	Yes	Yes	Yes	Yes	Yes	Yes
6F	Yes	No	Yes	No	Yes	No	No
7F	Yes	No	Yes	Yes	Yes	Yes	No
8F	Yes	No	Yes	Yes	Yes	Yes	Yes
9F	Yes	Yes	Yes	No	Yes	Yes	Yes
10F	Yes	Yes	Yes	No	Yes	Yes	Yes

Footnote: "Yes" indicates that the corresponding MEDAS adherence criterion was satisfied. M1, extra-virgin olive oil used as the principal culinary fat; M2, ≥4 tablespoons of olive oil/day; M3, ≥2 servings of vegetables/day; M4, ≥3 servings of fruit/day; M5, <1 serving of red or processed meat/day; M6, <1 serving of butter, margarine, or cream/day; M7, <1 sugar-sweetened beverage/day. F, female; M, male; MEDAS, Mediterranean Diet Adherence Screener.

Supplementary Table S4B. Participant-level responses to MEDAS items 8–14, total scores, and adherence categories.

Participant	M8	M9	M10	M11	M12	M13	M14	Total score	Category
1M	No	No	No	Yes	Yes	Yes	No	9	Intermediate
2M	No	No	No	No	Yes	Yes	No	8	Intermediate
3M	No	No	No	No	Yes	Yes	No	5	Low
4M	Yes	Yes	No	No	No	Yes	Yes	7	Intermediate
5M	No	No	No	Yes	No	Yes	Yes	10	High
6M	Yes	No	No	Yes	No	Yes	Yes	9	Intermediate
7M	No	No	Yes	Yes	No	Yes	Yes	8	Intermediate
8M	No	Yes	Yes	No	No	Yes	Yes	10	High
9M	Yes	No	No	No	Yes	Yes	No	8	Intermediate
10M	Yes	No	Yes	No	Yes	Yes	No	9	Intermediate
1F	No	No	Yes	No	No	No	Yes	6	Intermediate
2F	No	Yes	No	Yes	No	Yes	No	10	High
3F	No	No	No	Yes	Yes	Yes	Yes	8	Intermediate
4F	No	No	No	Yes	Yes	Yes	Yes	10	High
5F	No	No	No	Yes	No	Yes	Yes	9	Intermediate
6F	No	No	No	No	Yes	No	No	4	Low

7F	No	No	No	Yes	No	Yes	No	7	Intermediate
8F	No	Yes	No	Yes	Yes	Yes	No	10	High
9F	No	No	No	No	Yes	No	No	7	Intermediate
10F	No	Yes	Yes	Yes	No	Yes	Yes	11	High

Footnote: "Yes" indicates that the corresponding MEDAS adherence criterion was satisfied. M8, ≥7 glasses of wine/week; M9, ≥3 servings of legumes/week; M10, ≥3 servings of fish or seafood/week; M11, <3 servings of commercially produced sweets or pastries/week; M12, ≥3 servings of nuts/week; M13, preferential consumption of poultry rather than red meat; M14, consumption of sofrito ≥2 times/week. Total scores range from 0 to 14; adherence was classified as low (0–5), intermediate (6–9), or high (10–14). F, female; M, male; MEDAS, Mediterranean Diet Adherence Screener.

Supplementary Table S5. Participant-level responses to the nine UPF items, total scores, and operational consumption categories.

Participant	U1	U2	U3	U4	U5	U6	U7	U8	U9	Total score	Operational category
1M	1	0	0	0	1	0	0	0	0	2	Low
2M	1	1	3	0	0	3	1	1	0	10	Intermediate
3M	1	0	1	0	1	0	0	1	0	4	Low
4M	2	2	3	2	3	2	1	2	1	18	Intermediate
5M	0	0	2	0	0	0	0	2	0	4	Low
6M	1	2	1	0	0	1	0	0	0	5	Low
7M	0	1	1	1	0	2	0	1	1	7	Low
8M	0	1	2	0	2	0	0	1	0	6	Low
9M	2	2	2	0	1	0	1	2	1	11	Intermediate

10M	1	1	0	1	0	1	0	0	1	5	Low
1F	1	1	2	1	2	1	1	1	1	11	Intermediate
2F	0	0	0	0	2	0	0	0	0	2	Low
3F	1	3	1	0	0	1	0	2	0	8	Low
4F	2	1	1	1	2	1	2	1	0	11	Intermediate
5F	1	0	1	1	3	1	1	0	0	8	Low
6F	1	0	3	0	0	0	2	0	0	6	Low
7F	0	1	1	0	2	0	0	2	0	6	Low
8F	2	0	0	0	0	0	3	0	0	5	Low
9F	1	1	0	1	0	2	2	1	0	8	Low
10F	2	0	1	1	2	1	0	1	0	8	Low

Footnote: Item values represent reported consumption frequency: 0, never or almost never; 1, once/week; 2, 2–4 times/week; and 3, ≥5 times/week. U1, packaged savory snacks; U2, sugar-sweetened beverages; U3, industrially produced sweets; U4, fast food; U5, ready-to-eat meals; U6, processed meats; U7, sugar-sweetened breakfast cereals; U8, industrially produced sauces; U9, energy or functional beverages. These nine groups were specified in the study questionnaire before the current association analyses to provide a brief frequency-based representation of commonly consumed and readily recognizable industrially formulated foods consistent with the NOVA concept; they were not intended to constitute an exhaustive assessment of all NOVA group 4 foods. The four item-frequency levels were used to distinguish absent or negligible, occasional, recurrent, and frequent consumption. Items were equally weighted because no validated empirical basis was available for assigning differential weights. Total scores ranged from 0 to 27 and were operationally classified as low (0–9), intermediate (10–18), or high (19–27). These cutoffs were pragmatic partitions of the theoretical score range and were not validated intake thresholds. The continuous total score was used in the association analyses, whereas the operational categories were used exclusively for descriptive summaries and multivariate-dispersion diagnostics. The study-specific score has not been externally validated and does not quantify portion size, food weight, total energy intake, the energy contribution of ultra-processed foods, or the proportion of the total diet represented by ultra-processed foods. F, female; M, male; UPF, ultra-processed food.

Supplementary Table S6. Participant-level IPAQ-SF responses, corrected MET-min/week values, and physical-activity categories.

Participant	Vigorous, days/week	Vigorous, min/day	Moderate, days/week	Moderate, min/day	Walking, days/week	Walking, reported min/day	Walking, scoring min/day	Sitting, min/day	MET-min/week	Category
1M	3	60	0	0	5	40	40	120	2100	High
2M	5	120	0	0	2	60	60	120	5196	High
3M	5	90	2	60	2	60	60	120	4476	High
4M	2	90	5	20	1	30	30	240	1939	Moderate
5M	5	90	1	30	7	20	20	90	4182	High
6M	0	0	0	0	1	90	90	90	297	Low
7M	3	90	2	90	6	180	180	180	6444	High
8M	0	0	3	80	4	30	30	240	1356	Moderate
9M	1	60	0	0	7	60	60	180	1866	Moderate
10M	2	80	1	30	1	60	60	120	1598	Low
1F	3	60	1	60	7	30	30	240	2373	High
2F	4	90	0	0	7	120	120	300	5652	High
3F	3	50	2	30	2	60	60	300	1836	High
4F	0	0	0	0	3	30	30	120	297	Low

5F	0	0	3	40	0	0	0	120	480	Low
6F	2	60	2	60	4	30	30	120	1836	Moderate
7F	0	0	3	60	7	70	70	240	2337	Moderate
8F	0	0	0	0	0	0	0	120	0	Low
9F	3	60	0	0	7	240	180	480	5598	High
10F	1	10	2	30	3	30	30	90	617	Moderate

Footnote: MET-min/week was calculated as 8.0 × vigorous minutes × vigorous days + 4.0 × moderate minutes × moderate days + 3.3 × walking minutes × walking days. Activity durations exceeding 180 min/day were truncated to 180 min/day before calculation. This rule affected one participant (9F), whose reported walking duration was reduced from 240 to 180 min/day for scoring, changing the total from 6984 to 5598 MET-min/week without changing the High category. Sitting time was not included in the MET-min/week calculation. Categories were assigned according to the IPAQ-SF scoring criteria. F, female; IPAQ-SF, International Physical Activity Questionnaire–Short Form; M, male; MET, metabolic equivalent of task.

Supplementary Table S7A. Participant-level sleep timing, latency, duration, and subjective sleep quality.

Participant	Bedtime	Sleep latency, min	Wake time	Reported sleep duration, h	Subjective sleep quality
1M	00:00	5	09:00	9	Very good
2M	21:00	20	01:00	3.5	Fairly poor
3M	02:00	5	06:00	4	Very poor
4M	00:00	5	07:00	7	Fairly good
5M	00:00	60	07:00	6	Fairly poor
6M	00:00	5	07:00	7	Fairly good
7M	00:00	5	07:00	7	Fairly good
8M	23:00	10	07:00	8	Very good
9M	00:00	15	07:00	7	Fairly poor
10M	23:00	15	07:00	8	Very good
1F	23:00	60	08:00	8	Fairly good
2F	23:00	10	07:00	8	Fairly good
3F	01:00	10	08:00	7	Fairly good
4F	23:00	60	05:00	5	Fairly poor
5F	22:00	5	07:00	9	Fairly good
6F	00:00	20	07:00	6.5	Fairly good
7F	23:00	30	07:00	7.5	Fairly good
8F	21:00	10	06:00	9	Fairly good
9F	00:00	10	07:00	7	Fairly good
10F	23:00	5	07:00	8	Fairly good

Footnote: Values represent participants' reported habitual sleep characteristics during the reference period. Sleep duration is self-reported and was not calculated by subtracting bedtime from wake time. F, female; M, male.

Supplementary Table S7B. Participant-level frequency of sleep-related disturbances.

Participant	Difficulty falling asleep	Night/early-morning awakening	Bathroom use	Breathing difficulty	Thermal discomfort	Pain/discomfort
1M	Never	Never	Never	Never	Never	Never
2M	1–2 times/week	1–2 times/week	≥3 times/week	Never	Never	Never
3M	<1 time/week	Never	Never	Never	Never	Never
4M	<1 time/week	1–2 times/week	1–2 times/week	Never	Never	<1 time/week
5M	≥3 times/week	≥3 times/week	1–2 times/week	Never	≥3 times/week	Never
6M	<1 time/week	≥3 times/week	Never	<1 time/week	<1 time/week	<1 time/week
7M	1–2 times/week	Never	Never	Never	<1 time/week	Never
8M	Never	Never	Never	Never	Never	Never
9M	Never	≥3 times/week	Never	Never	≥3 times/week	Never
10M	Never	Never	Never	Never	Never	Never
1F	<1 time/week	<1 time/week	Never	Never	<1 time/week	Never
2F	<1 time/week	1–2 times/week	Never	Never	1–2 times/week	Never
3F	<1 time/week	<1 time/week	Never	Never	1–2 times/week	Never
4F	≥3 times/week	1–2 times/week	1–2 times/week	<1 time/week	Never	Never
5F	Never	<1 time/week	Never	Never	Never	Never

6F	<1 time/week	1–2 times/week	Never	Never	Never	Never
7F	<1 time/week	≥3 times/week	1–2 times/week	Never	1–2 times/week	Never
8F	Never	1–2 times/week	Never	Never	Never	Never
9F	Never	1–2 times/week	<1 time/week	Never	Never	Never
10F	Never	Never	<1 time/week	Never	Never	Never

Footnote: Frequencies refer to sleep-related disturbances reported during the month preceding questionnaire completion. Response categories were never, <1 time/week, 1–2 times/week, and ≥3 times/week. F, female; M, male.

Supplementary Table S7C. Participant-level sleep-medication use and daytime consequences.

Participant	Sleep-medication use	Difficulty staying awake	Difficulty maintaining enthusiasm/performance
1M	Never	Never	None
2M	Never	1–2 times/week	Moderate
3M	Never	Never	None
4M	Never	<1 time/week	Mild
5M	Never	Never	Mild
6M	Never	<1 time/week	Mild
7M	Never	<1 time/week	Mild
8M	Never	Never	Mild
9M	Never	1–2 times/week	Moderate
10M	Never	Never	None
1F	Never	Never	Moderate
2F	Never	Never	Mild
3F	Never	Never	Mild
4F	≥3 times/week	Never	Moderate
5F	Never	Never	None
6F	Never	≥3 times/week	Mild
7F	Never	<1 time/week	None
8F	Never	Never	Mild
9F	≥3 times/week	<1 time/week	Moderate
10F	Never	Never	None

Footnote: Sleep-medication use and difficulty staying awake refer to the month preceding questionnaire completion. Difficulty maintaining enthusiasm or performance was reported as none, mild, moderate, or severe. F, female; M, male.

Supplementary Table S8. Participant-level adapted sleep-component scores, total scores, and descriptive score ranges.

Participant	C1	C2	C3	C4	C5	C6	C7	Adapted sleep score	Descriptive score range
1M	0	0	0	0	0	0	0	0	0–4
2M	2	2	3	0	1	0	2	10	10–14
3M	3	1	3	0	0	0	0	7	5–9
4M	1	1	1	0	1	0	1	5	5–9
5M	2	3	1	0	2	0	1	9	5–9
6M	1	1	1	0	2	0	1	6	5–9
7M	1	1	1	0	1	0	1	5	5–9
8M	0	0	0	0	0	0	1	1	0–4
9M	2	0	1	0	2	0	2	7	5–9
10M	0	0	0	0	0	0	0	0	0–4
1F	1	2	0	0	1	0	1	5	5–9
2F	1	1	0	0	1	0	1	4	0–4
3F	1	1	1	0	1	0	1	5	5–9
4F	2	3	2	1	1	3	1	13	10–14
5F	1	0	0	0	1	0	0	2	0–4

6F	1	1	1	0	1	0	2	6	5–9
7F	1	1	0	0	2	0	1	5	5–9
8F	1	0	0	0	1	0	1	3	0–4
9F	1	0	1	0	1	3	2	8	5–9
10F	1	0	0	0	1	0	0	2	0–4

Footnote: C1, subjective sleep quality; C2, sleep latency; C3, sleep duration; C4, habitual sleep efficiency; C5, sleep disturbances; C6, use of sleep medication; C7, daytime dysfunction. Each component ranges from 0 to 3, and the adapted sleep score is the sum of the seven component scores (theoretical range, 0–21), with higher values indicating greater reported sleep difficulty. For descriptive summaries only, total scores were grouped into the prespecified ranges of 0–4, 5–9, 10–14, and 15–21. These ranges were pragmatic descriptive groupings and were not assigned qualitative or diagnostic labels. The instrument represents a study-specific adaptation of seven principal sleep-related domains and does not constitute administration of the complete validated Pittsburgh Sleep Quality Index. Neither the adapted total score nor its descriptive ranges should be interpreted as a validated measure of sleep quality, a clinically meaningful classification, or a diagnostic instrument. The continuous adapted sleep score was used in the association analyses; the score ranges were used only for descriptive summaries and multivariate-dispersion diagnostics. F, female; M, male.

Supplementary Table S9. Sequencing performance, quality-control metrics, and OTU-table characteristics for the 20 fresh-stool microbiome profiles.

Characteristic	Mean ± SD	Median [IQR]	Range or n (%)
Total reads, n	186,570.8 ± 48,767.9	198,405.0 [175,864.0–207,102.5]	24,738–256,997
Quality-filtered reads, n	168,200.4 ± 46,187.5	177,995.5 [161,365.8–188,979.5]	14,311–230,078
Low-quality reads, n	18,370.5 ± 3,963.6	18,634.0 [16,694.3–20,790.0]	10,427–26,919
Unclassified reads, n	30,288.3 ± 8,947.0	32,558.0 [26,789.0–35,587.3]	8569–42,733
RDP-classified reads, n	144,753.9 ± 39,989.3	154,161.0 [138,772.8–164,516.8]	11,595–188,401
Classified OTU reads contributing to alpha-diversity calculation, n	114,475.4 ± 36,408.1	122,485.5 [109,322.8–132,687.8]	3026–161,259
Observed OTU richness, n	499.6 ± 122.4	537.0 [477.3–573.3]	164–664

Footnote: Values are presented as mean ± standard deviation (SD), median [interquartile range (IQR)], and range across the 20 fresh-stool microbiome profiles. Percentages were calculated separately for each sample before summarization. Unclassified reads correspond to sequences reported as "Unclassified Bacteria and Archaea" in the sequencing-statistics output. RDP-classified reads were obtained from the sequencing-statistics output. Classified OTU reads represent the participant-level sums across the 1755 non-aggregate rows retained from the exported OTU count table after exclusion of the software-generated "taxa1" row. Because the RDP-classified and OTU-read counts derive from different exported pipeline outputs, they are not directly interchangeable. Observed OTU richness was independently calculated as the number of OTUs with a positive count. IQR, interquartile range; OTU, operational taxonomic unit; RDP, Ribosomal Database Project; SD, standard deviation.

Supplementary Table S10. Descriptive statistics for the ten most abundant genus-level taxonomic groups in the 20 fresh-stool microbiome profiles.

Genus-level taxonomic group	Mean, %	SD, %	Median, %	IQR, %	Range, %	Detected, n (%)
Bacteroides	15.34	7.61	15.06	9.77–20.85	2.87–30.34	20 (100)
Unclassified Lachnospiraceae	11.27	3.20	11.40	8.47–13.76	5.68–18.25	20 (100)
Faecalibacterium	7.69	3.24	8.16	5.19–10.52	0.93–14.29	20 (100)
Unclassified Clostridiales	4.62	1.84	4.85	3.42–5.57	0.97–8.11	20 (100)
Unclassified Ruminococcaceae	3.99	2.35	3.68	2.76–5.06	0.38–10.46	20 (100)
Unclassified Bacteroidales	3.91	1.09	3.84	3.44–4.50	1.08–6.23	20 (100)
Bifidobacterium	3.56	2.72	2.68	1.46–5.75	0.08–8.71	20 (100)
Blautia	3.50	2.08	2.62	2.13–4.38	1.07–8.64	20 (100)
Prevotella	3.24	6.56	0.07	0.00–2.37	0.00–22.72	15 (75.0)
Lachnospiraceae incertae sedis	2.77	1.40	2.63	1.78–3.99	0.12–5.64	20 (100)

Footnote: Taxonomic groups are ordered by decreasing mean relative abundance. Relative abundances are expressed as percentages of the total genus-level abundance assigned within each sample. Prevalence represents the number and percentage of participants in whom the taxonomic group had an observed relative abundance greater than zero. The table includes both formally assigned genera and incompletely classified groups retained at the lowest taxonomic level available in the analytical output. Because values are rounded to two decimal places, very low positive abundances may appear as 0.00%. SD, standard deviation; IQR, interquartile range.

Supplementary Table S11. Participant-level classified OTU-read depth, observed OTU richness, and independently recalculated full-precision alpha-diversity values.

Participant	Classified OTU reads, n	Observed OTUs, n	Shannon index	Pielou's evenness
1M	161,259	511	3.956165	0.634370
2M	131,042	536	4.092031	0.651169
3M	134,661	552	4.091901	0.648114
4M	132,030	400	3.837080	0.640424
5M	92,772	550	4.181052	0.662616
6M	103,311	445	4.019708	0.659177
7M	128,177	587	4.249916	0.666651
8M	145,268	573	4.151198	0.653641
9M	117,096	488	4.121766	0.665841
10M	123,944	664	4.240331	0.652531
1F	121,027	520	3.809393	0.609130
2F	37,509	242	3.134325	0.571026
3F	117,147	501	4.070131	0.654719
4F	135,423	550	4.126672	0.653998
5F	145,791	613	4.153521	0.647131
6F	109,877	598	4.269721	0.667813
7F	3026	164	3.543264	0.694776
8F	109,323	574	4.087445	0.643426
9F	109,322	538	3.974326	0.632064
10F	131,502	385	3.749108	0.629759

Footnote: Classified OTU reads were calculated as the participant-level sums across the 1755 non-aggregate rows retained from the exported OTU count table after exclusion of the software-generated "taxa1" row. Observed OTU richness represents

the number of OTUs with a positive count. Shannon diversity was calculated using natural logarithms, and Pielou's evenness was calculated as Shannon diversity divided by the natural logarithm of observed OTU richness. Full-precision values are reported because they constituted the outcomes used in the revised statistical analyses. F, female; M, male; OTU, operational taxonomic unit.

Supplementary Table S12. Participant-level coordinates from the Bray–Curtis and Aitchison principal coordinates analyses.

Participant	Bray-Curtis Axis 1	Bray-Curtis Axis 2	Aitchison Axis 1	Aitchison Axis 2
1M	−0.103625	0.040708	0.813804	0.882583
2M	0.036284	−0.070656	−3.705254	6.189844
3M	0.008504	−0.018034	9.114275	0.415430
4M	−0.157348	0.022442	0.992254	−4.885797
5M	0.069079	−0.134567	−3.934386	12.215405
6M	0.093339	0.005709	−6.609759	−4.116096
7M	−0.054738	−0.034354	−1.318652	0.512833
8M	0.101760	0.067456	−2.378259	−0.548383
9M	0.030757	0.052296	−7.204749	−2.352346
10M	0.030757	−0.034343	2.090590	−1.161475
1F	0.015195	−0.021826	0.896563	−1.603025
2F	−0.053088	−0.020631	−0.963525	−1.365997
3F	0.064380	0.030099	−0.925013	2.291021
4F	−0.005863	0.006286	1.372303	−1.965221
5F	−0.026621	0.041173	3.135992	−3.765038
6F	0.080715	0.009066	−0.487817	−1.127833
7F	−0.097070	−0.040871	8.394437	−5.432666
8F	0.038284	0.019690	−0.768673	2.722439
9F	−0.042006	0.043495	1.046846	0.430131

10F	0.030132	0.037161	−4.557006	1.668192

Footnote: Coordinates correspond to Figure 3 and are displayed to six decimal places; full-precision values were used to generate the ordinations. Bray–Curtis dissimilarity was calculated from observed genus-level relative abundances. Aitchison distance was calculated as Euclidean distance in centered log-ratio space after adding a proportional pseudocount of 1×10^{-6}. Axis 1 and axis 2 represented 25.06% and 23.87% of the Bray–Curtis variation and 15.91% and 10.26% of the Aitchison variation, respectively. The signs and orientations of PCoA axes are arbitrary and may be reversed without altering the represented intersample relationships. F, female; M, male; PCoA, principal coordinates analysis.

Supplementary Table S13. Participant-level relative abundances of the eight prespecified bacterial genera.

Participant	*Bacteroides*	*Faecalibacterium*	*Bifidobacterium*	*Blautia*	*Prevotella*	*Roseburia*	*Ruminococcus*	*Akkermansia*
1M	10.1154	8.7983	5.7987	1.7010	19.7763	1.2663	0.3404	0.9786
2M	17.5844	14.2855	5.7295	1.9803	0.0053	2.3038	1.5781	0.3426
3M	14.0501	9.5744	6.7978	2.0496	0.1233	0.1960	1.6493	0.1329
4M	8.7457	8.9555	0.7809	5.3609	22.7236	0.8324	1.1376	0.0008
5M	30.3367	10.6993	2.7099	4.4054	0.0086	1.9165	0.0744	0.9992
6M	16.7427	4.2725	0.0784	4.3674	0.0039	0.4762	2.7945	3.5959
7M	18.6235	3.9648	1.9122	4.8737	0.0000	0.6117	1.2475	0.9440
8M	11.2537	9.1940	4.5488	2.1567	0.2533	0.0454	1.2783	0.0089
9M	11.9304	6.2743	1.1546	2.2170	0.0000	2.9104	0.8198	0.0043
10M	16.0750	6.6006	3.8227	3.6670	0.6132	2.2906	3.9405	0.0032
1F	20.7268	5.2137	8.5749	8.6444	0.0017	0.3974	0.0033	0.0025
2F	2.8740	0.9304	6.5051	8.3847	0.0000	0.0027	0.0053	0.0213
3F	27.0310	10.5474	2.3039	2.5114	0.0000	4.8580	0.9279	0.0060
4F	7.6206	7.5194	2.6421	3.6065	6.6030	0.9806	2.0639	0.3522
5F	21.2359	10.5391	0.6262	2.6620	2.4707	1.7429	0.3917	0.0453
6F	21.4121	10.8594	4.6488	2.3927	0.0036	1.1021	2.1306	0.0046

7F	4.1309	10.5089	0.0991	3.8334	7.8321	0.9584	0.8592	0.2313
8F	7.0333	3.8290	1.5614	1.0721	2.3307	0.4153	0.6613	3.0378
9F	13.7420	6.1616	2.1030	1.5505	1.9868	1.0135	1.2550	0.7876
10F	25.6057	5.1109	8.7086	2.5863	0.0000	0.6494	1.7802	0.0183

Footnote: Values are observed relative abundances expressed as percentages. Values are displayed to four decimal places to preserve low positive abundances. A displayed value of 0.0000% indicates that the genus was not detected in that participant. F, female; M, male.

Supplementary Table S14. Participant-level Prevotella and Bacteroides abundances, ratios, and descriptive predominance categories.

Participant	Prevotella, %	Bacteroides, %	Untransformed P/B ratio	Natural-log-transformed P/B ratio	Descriptive category
1M	19.776261	10.115404	1.955064	0.670383	Relative Prevotella predominance
2M	0.005342	17.584439	0.000304	−7.955432	Relative Bacteroides predominance
3M	0.123273	14.050096	0.008774	−4.729365	Relative Bacteroides predominance
4M	22.723623	8.745740	2.598251	0.954780	Relative Prevotella predominance
5M	0.008623	30.336740	0.000284	−8.074175	Relative Bacteroides predominance
6M	0.003872	16.742651	0.000231	−8.178613	Relative Bacteroides predominance
7M	0.000000	18.623466	0.000000	−10.023066	Relative Bacteroides predominance
8M	0.253325	11.253683	0.022510	−3.790595	Relative Bacteroides predominance
9M	0.000000	11.930382	0.000000	−9.577756	Relative Bacteroides predominance
10M	0.613180	16.075002	0.038145	−3.265067	Relative Bacteroides predominance
1F	0.001653	20.726780	0.000080	−9.031453	Relative Bacteroides predominance
2F	0.000000	2.873977	0.000000	−8.154583	Relative Bacteroides predominance
3F	0.000000	27.030995	0.000000	−10.395614	Relative Bacteroides predominance
4F	6.603014	7.620567	0.866473	−0.143308	Relative Bacteroides predominance
5F	2.470660	21.235879	0.116344	−2.150911	Relative Bacteroides predominance
6F	0.003640	21.412124	0.000170	−8.475102	Relative Bacteroides predominance
7F	7.832122	4.130866	1.896000	0.639652	Relative Prevotella predominance
8F	2.330708	7.033287	0.331382	−1.104245	Relative Bacteroides predominance
9F	1.986791	13.741973	0.144578	−1.933578	Relative Bacteroides predominance
10F	0.000000	25.605694	0.000000	−10.341446	Relative Bacteroides predominance

Footnote: The untransformed P/B ratio was calculated directly from the observed relative abundances as Prevotella/Bacteroides. The natural-log-transformed ratio was calculated as ln[(Prevotella + c)/(Bacteroides + c)], using proportional abundances and $c = 8.262619 \times 10^{-6}$. Relative Bacteroides predominance was defined as an untransformed ratio <1 and relative Prevotella predominance as a ratio >1. These categories were used only for descriptive purposes and do not represent formal enterotypes. Values are displayed to six decimal places; full-precision values were retained for all analyses. F, female; M, male; P/B, Prevotella/Bacteroides.

Supplementary Table S15. Model fit, diagnostic assessment, and robustness analyses for associations of dietary and lifestyle exposures with independently recalculated OTU-level alpha diversity: (A) model fit and exposure-specific effect size; (B) regression diagnostics and leave-one-out influence analysis; (C) HC3 and Freedman–Lane permutation inference; (D) sensitivity to the original untruncated IPAQ-SF score; (E) sensitivity after repeated rarefaction to 3026 classified OTU reads; and (F) sensitivity after exclusion of the lowest-depth participant.

Panel A. Model fit and exposure-specific effect size (n = 20)

Outcome	Exposure	Model R^2	Adjusted R^2	Partial R^2
**Shannon index**	MEDAS	0.167	0.010	0.026
	UPF score	0.154	−0.005	0.011
	IPAQ-SF	0.264	0.126	0.140
	Adapted Sleep score	0.167	0.011	0.026
**Pielou's evenness**	MEDAS	0.159	0.002	0.058
	UPF score	0.120	−0.045	0.014
	IPAQ-SF	0.151	−0.008	0.049
	Adapted Sleep score	0.185	0.032	0.087

Panel B. Regression diagnostics and leave-one-out assessment (n=20)

Outcome	Exposure	Shapiro–Wilk p	Breusch–Pagan p	Maximum VIF	Maximum Cook's D (participant)	n with Cook's D > 0.20	LOO β sign reversal	LOO p-value range
**Shannon index**	MEDAS	0.032	0.311	1.006	0.497 (2F)	2	Yes	0.356–0.874
	UPF score	0.076	0.263	1.011	1.264 (4M)	2	Yes	0.090–0.837
	IPAQ-SF	0.433	0.072	1.530	0.742 (2F)	1	No	0.063–0.659
	Adapted Sleep score	0.065	0.333	1.105	0.396 (2F)	1	No	0.348–0.844
**Pielou's evenness**	MEDAS	0.381	0.358	1.006	0.402 (2F)	1	No	0.156–0.708
	UPF score	0.382	0.245	1.011	0.783 (4M)	2	Yes	0.165–0.827
	IPAQ-SF	0.578	0.110	1.530	0.673 (2F)	2	Yes	0.175–0.748
	Adapted Sleep score	0.338	0.416	1.105	0.330 (2F)	1	No	0.182–0.405

Panel C. HC3 and Freedman–Lane permutation inference (n = 20)

Outcome	Exposure	HC3 SE	HC3 95% CI	HC3 p-value	HC3 p-FDR	Permutation p-value	Permutation p-FDR
Shannon index	MEDAS	0.04513	−0.11780 to 0.07354	0.631	0.841	0.523	0.657
	UPF score	0.04008	−0.07795 to 0.09198	0.863	0.863	0.657	0.657
	IPAQ-SF	5.12×10^{-5}	-1.67×10^{-4} to 5.04×10^{-5}	0.273	0.828	0.124	0.657
	Adapted Sleep score	0.01694	−0.02315 to 0.04866	0.462	0.828	0.523	0.657
Pielou's evenness	MEDAS	0.003786	−0.011157 to 0.004896	0.420	0.828	0.340	0.657
	UPF score	0.003156	−0.005946 to 0.007433	0.817	0.863	0.620	0.657
	IPAQ-SF	4.90×10^{-6}	-1.36×10^{-5} to 7.14×10^{-6}	0.518	0.828	0.376	0.657
	Adapted Sleep score	0.001269	−0.000495 to 0.004888	0.103	0.822	0.232	0.657

Panel D. Sensitivity to the original untruncated IPAQ-SF score

Outcome	IPAQ-SF specification	β	95% CI	p-value	p-FDR	Partial R^2
Shannon index	Primary score with 180-min/day truncation	-5.82×10^{-5}	-1.35×10^{-4} to 1.84×10^{-5}	0.127	0.684	0.140
	Original untruncated score	-5.01×10^{-5}	-1.23×10^{-4} to 2.32×10^{-5}	0.167	0.333	0.116
Pielou's evenness	Primary score with 180-min/day truncation	-3.24×10^{-6}	-1.08×10^{-5} to 4.31×10^{-6}	0.376	0.684	0.049
	Original untruncated score	-2.90×10^{-6}	-1.01×10^{-5} to 4.26×10^{-6}	0.403	0.403	0.044

Panel E. Sensitivity after repeated rarefaction to 3026 classified OTU reads (n = 20)

Outcome	Exposure	β	95% CI	p-value	p-FDR	Partial R^2
Shannon index	MEDAS	−0.02192	−0.09088 to 0.04703	0.510	0.646	0.028
	UPF score	0.00702	−0.02724 to 0.04128	0.670	0.670	0.012
	IPAQ-SF	-5.59×10^{-5}	-1.29×10^{-4} to 1.70×10^{-5}	0.124	0.495	0.142
	Adapted Sleep score	0.01288	−0.02670 to 0.05246	0.500	0.646	0.029
Pielou's evenness	MEDAS	−0.002133	−0.009836 to 0.005571	0.565	0.646	0.021
	UPF score	0.001593	−0.002150 to 0.005336	0.380	0.646	0.048
	IPAQ-SF	-6.85×10^{-6}	-1.48×10^{-5} to 1.14×10^{-6}	0.088	0.495	0.171
	Adapted Sleep score	0.002203	−0.002115 to 0.006520	0.295	0.646	0.068

Panel F. Sensitivity after exclusion of participant 7F (n = 19)

Outcome	Exposure	β	95% CI	p-value	p-FDR	Partial R^2
Shannon index	MEDAS	−0.03070	−0.10142 to 0.04002	0.369	0.566	0.054
	UPF score	0.00538	−0.02992 to 0.04068	0.750	0.750	0.007
	IPAQ-SF	-5.24×10^{-5}	-1.29×10^{-4} to 2.38×10^{-5}	0.163	0.502	0.125
	Adapted Sleep score	0.01330	−0.02723 to 0.05383	0.495	0.566	0.032
Pielou's evenness	MEDAS	−0.002034	−0.008066 to 0.003999	0.483	0.566	0.033
	UPF score	0.000998	−0.001940 to 0.003936	0.480	0.566	0.034
	IPAQ-SF	-4.30×10^{-6}	-1.08×10^{-5} to 2.15×10^{-6}	0.175	0.502	0.119

Adapted Sleep score	0.002117	−0.001157 to 0.005391	0.188	0.502	0.112

Footnote: All models were adjusted for age and sex, with female as the reference category. Panels A–C use the full-precision alpha-diversity values independently recalculated from the exported OTU count table. The Cook's-distance screening threshold was 4/n = 0.20; observations exceeding this threshold were examined but were not automatically excluded. Panel D compares the primary IPAQ-SF score, calculated after truncating individual activity durations exceeding 180 min/day, with the score calculated from the original untruncated responses. The primary and untruncated p-FDR values derive from their respective, separately adjusted model families and should not be compared as though they belonged to a common multiplicity family. Panel E uses participant-level alpha-diversity values averaged across 1000 rarefactions without replacement to the common minimum depth of 3026 classified OTU reads. Panel F excludes participant 7F, who had the lowest classified OTU-read depth. Benjamini–Hochberg correction was applied separately across the eight rarefied models and across the eight participant-exclusion models. HC3 and Freedman–Lane p-values were also adjusted separately within their respective eight-model families. β, unstandardized regression coefficient; CI, confidence interval; F, female; FDR, false discovery rate; HC3, heteroscedasticity-consistent covariance estimator; IPAQ-SF, International Physical Activity Questionnaire–Short Form; LOO, leave-one-out; MEDAS, Mediterranean Diet Adherence Screener; MET, metabolic equivalent of task; OTU, operational taxonomic unit; SE, standard error; UPF, ultra-processed food; VIF, variance inflation factor.

Supplementary Table S16. PERMANOVA model results, multivariate-dispersion diagnostics, and robustness analyses for associations of dietary and lifestyle exposures with overall gut microbiota composition in the 20 participants: (A) complete-model results for the primary analyses; (B) multivariate-dispersion diagnostics; (C) sensitivity analyses using prevalence-filtered genus-level compositions; (D) Aitchison-distance sensitivity analyses using alternative pseudocounts; and (E) sensitivity analyses using the original untruncated IPAQ-SF score.

Panel A. Complete-model results for the primary PERMANOVA analyses

Distance measure	Exposure	Overall R^2	Overall pseudo-F	Overall p-value
**Bray–Curtis**	MEDAS	0.1370	0.846	0.707
	UPF score	0.1457	0.910	0.583
	IPAQ-SF, MET-min/week	0.1670	1.069	0.365
	Adapted Sleep score	0.1363	0.842	0.711
**Aitchison**	MEDAS	0.1462	0.914	0.738
	UPF score	0.1480	0.926	0.693
	IPAQ-SF, MET-min/week	0.1486	0.931	0.688
	Adapted Sleep score	0.1594	1.011	0.450

Panel B. Multivariate-dispersion diagnostics based on operational exposure categories

Distance measure	Exposure classification	Categories, n	Smallest category, n	F	p-value	p-FDR
**Bray–Curtis**	MEDAS adherence	3	2	3.079	0.080	0.643
	UPF consumption	2	5	0.087	0.787	0.995

	IPAQ-SF physical activity	3	5	0.021	0.985	0.995
	Adapted Sleep score	3	2	1.454	0.251	0.995
	MEDAS adherence	3	2	0.494	0.632	0.995
**Aitchison**	UPF consumption	2	5	<0.001	0.995	0.995
	IPAQ-SF physical activity	3	5	0.659	0.530	0.995
	Adapted Sleep score	3	2	0.136	0.883	0.995

Panel C. Sensitivity analyses using prevalence-filtered genus-level compositions

Distance measure	Exposure	Marginal R^2	Pseudo-F	p-value	p-FDR
**Bray–Curtis**	MEDAS	0.0348	0.645	0.784	0.799
	UPF score	0.0436	0.816	0.560	0.799
	IPAQ-SF, MET-min/week	0.0650	1.248	0.246	0.799
	Adapted Sleep score	0.0342	0.634	0.799	0.799
**Aitchison**	MEDAS	0.0435	0.812	0.794	0.799
	UPF score	0.0471	0.883	0.660	0.799
	IPAQ-SF, MET-min/week	0.0477	0.894	0.642	0.799
	Adapted Sleep score	0.0605	1.151	0.238	0.799

Panel D. Aitchison-distance sensitivity analyses using alternative pseudocounts

Pseudocount	Exposure	Marginal R²	Pseudo-F	p-value	p-FDR
1×10^{-8}	MEDAS	0.0496	0.938	0.586	0.758
	UPF score	0.0496	0.938	0.578	0.758
	IPAQ-SF, MET-min/week	0.0456	0.857	0.758	0.758
	Adapted Sleep score	0.0562	1.071	0.321	0.758
1×10^{-4}	MEDAS	0.0358	0.665	0.928	0.928
	UPF score	0.0400	0.747	0.812	0.928
	IPAQ-SF, MET-min/week	0.0589	1.126	0.276	0.551
	Adapted Sleep score	0.0626	1.202	0.204	0.551

Panel E. Sensitivity analyses using the original untruncated IPAQ-SF score

Distance measure	Exposure	Marginal R²	Pseudo-F	p-value	p-FDR
Bray–Curtis	Untruncated IPAQ-SF, MET-min/week	0.0532	1.008	0.415	0.788
Aitchison	Untruncated IPAQ-SF, MET-min/week	0.0440	0.824	0.788	0.788

Footnote: Panel A reports the variance explained, pseudo-F statistic, and permutation-derived p-value for each complete PERMANOVA model containing the exposure, age, and sex. Panel B reports diagnostic tests of homogeneity of multivariate dispersion based on the operational categories of each exposure; distances to group spatial medians were calculated with small-sample bias adjustment. Panel C reports marginal exposure effects after retaining the 161 genus-level features detected in at least four participants and reclosing each composition to sum to one. Panel D reports marginal effects obtained after calculating Aitchison distances with pseudocounts of 1×10^{-8} or 1×10^{-4}. Panel E reports marginal effects obtained using the original IPAQ-SF score without truncation of reported activity durations. All permutation-based analyses used the same matrix of 9999 unrestricted permutations. Benjamini–Hochberg adjustment was applied to the eight dispersion tests in Panel B and separately

within each sensitivity-analysis family in Panels C–E. FDR, false discovery rate; IPAQ-SF, International Physical Activity Questionnaire–Short Form; MEDAS, Mediterranean Diet Adherence Screener; MET, metabolic equivalent of task; PERMANOVA, permutational multivariate analysis of variance; UPF, ultra-processed food.

Supplementary Table S17. Diagnostic assessment and robustness analyses for associations of dietary and lifestyle exposures with the CLR coordinates of the eight prespecified bacterial genera in the 20 participants: (A) comparison of conventional, HC3, and Freedman–Lane inference; (B) regression-diagnostic and leave-one-out summary; (C) sensitivity to alternative abundance representations; (D) sensitivity to alternative CLR pseudocounts; and (E) sensitivity to use of the original untruncated IPAQ-SF score.

Panel A. Comparison of inferential approaches

Inferential approach	Smallest p-value	Exposure–genus association	p-FDR for the same association	Associations with p-FDR < 0.05
Conventional OLS	0.0557	UPF–*Akkermansia*	0.8147	0
HC3	0.0435	UPF–*Akkermansia*	0.9819	0
Freedman–Lane permutation	0.0485	UPF–*Akkermansia*	0.8113	0

Panel B. Regression-diagnostic and leave-one-out summary

Diagnostic assessment	Result
Shapiro–Wilk residual-normality test	$p < 0.05$ in 8 of 32 models
Breusch–Pagan heteroscedasticity test	$p < 0.05$ in 1 of 32 models: MEDAS–Bacteroides
Maximum variance inflation factor	No model had a VIF > 5
Cook's distance > 4/n = 0.20	At least one observation in 30 of 32 models
Cook's distance > 1.00	None
Largest Cook's distance	0.993 in the UPF–Prevotella model; participant 4M
Leave-one-out coefficient sign reversal	14 of 32 models
Leave-one-out nominal p-value < 0.05	At least one iteration in 3 of 32 models: UPF–Roseburia, UPF–Akkermansia, and IPAQ-SF–Akkermansia; none represented a stable multiplicity-adjusted association
Observations excluded from primary models	None

Panel C. Sensitivity to alternative taxonomic-abundance representations

Outcome representation	Smallest nominal p-value	Exposure–genus association	Minimum p-FDR	Direction concordant with primary CLR model	Models with Shapiro–Wilk p < 0.05
CLR, primary	0.0557	UPF–*Akkermansia*	0.8147	32/32	8/32
Arcsine-square-root-transformed proportion	0.1482	MEDAS–*Faecalibacterium*	0.8188	29/32	6/32
Natural-log-transformed abundance	0.0556	UPF–*Akkermansia*	0.7880	30/32	15/32
Untransformed relative abundance	0.1666	UPF–*Prevotella*	0.9906	29/32	13/32

Panel D. Sensitivity to the CLR pseudocount

CLR pseudocount	Smallest nominal p-value	Exposure–genus association	Minimum p-FDR	Direction concordant with primary model	Models with Shapiro–Wilk p < 0.05
1×10^{-8}	0.0565	UPF–*Akkermansia*	0.8992	31/32	3/32
1×10^{-6}, primary	0.0557	UPF–*Akkermansia*	0.8147	32/32	8/32
1×10^{-4}	0.1177	MEDAS–*Faecalibacterium*	0.7724	30/32	11/32

Panel E. Sensitivity analysis using the original untruncated IPAQ-SF score

Genus	β	SE	95% CI	p-value	p-FDR	Partial R²
Bacteroides	7.08×10^{-5}	9.29×10^{-5}	-1.26×10^{-4} to 2.68×10^{-4}	0.457	0.707	0.035
Prevotella	-7.05×10^{-4}	6.23×10^{-4}	-2.03×10^{-3} to 6.17×10^{-4}	0.275	0.707	0.074
Faecalibacterium	-9.64×10^{-6}	8.90×10^{-5}	-1.98×10^{-4} to 1.79×10^{-4}	0.915	0.915	0.001
Bifidobacterium	4.21×10^{-5}	1.67×10^{-4}	-3.13×10^{-4} to 3.97×10^{-4}	0.805	0.915	0.004
Blautia	1.13×10^{-4}	1.19×10^{-4}	-1.40×10^{-4} to 3.66×10^{-4}	0.357	0.707	0.053
Roseburia	-1.48×10^{-4}	2.31×10^{-4}	-6.37×10^{-4} to 3.41×10^{-4}	0.530	0.707	0.025
Ruminococcus	-2.16×10^{-4}	2.40×10^{-4}	-7.26×10^{-4} to 2.93×10^{-4}	0.382	0.707	0.048
Akkermansia	5.08×10^{-4}	3.48×10^{-4}	-2.30×10^{-4} to 1.25×10^{-3}	0.164	0.707	0.117

Footnote: Panel A compares the smallest nominal and FDR-adjusted p-values obtained using conventional model-based inference, HC3 heteroscedasticity-consistent inference, and Freedman–Lane permutation tests with 9,999 permutations. Multiplicity correction was applied separately to the 32 tests under each inferential approach. Panel B summarizes diagnostics for the 32 primary CLR models; Cook's distance was used as a screening measure, and no observation was excluded solely on the basis of diagnostic results. Panel C compares the primary CLR representation with three alternative representations of genus-level relative abundance. Natural-log-transformed abundances were calculated as $\ln(x + 1 \times 10^{-6})$, where x denotes proportional relative abundance. Panel D compares CLR coordinates calculated using three pseudocounts expressed in proportional-abundance units. Direction concordance indicates the number of exposure–genus coefficients having the same sign as the corresponding primary CLR coefficient. In Panels C and D, multiplicity correction was applied separately within each representation or pseudocount. Panel E reports age- and sex-adjusted models using the IPAQ-SF score calculated from the original responses without truncation at 180 min/day; the coefficient direction was concordant with the primary truncated-score analysis for all eight genera. CI, confidence interval; CLR, centered log-ratio; FDR, false discovery rate; IPAQ-SF, International Physical Activity Questionnaire–Short Form; MEDAS, Mediterranean Diet Adherence Screener; OLS, ordinary least squares; SE, standard error; UPF, ultra-processed food; VIF, variance inflation factor.

Supplementary Table S18. Model fit, diagnostic assessment, influence analyses, and robustness of associations between dietary and lifestyle exposures and the natural-log-transformed *Prevotella*/*Bacteroides* ratio: (A) model fit and regression diagnostics; (B) leave-one-out analyses; (C) HC3 and Freedman–Lane inference; (D) sensitivity to IPAQ-SF truncation; and (E) temporary exclusion of the lowest-depth microbiome profile.

Panel A. Model fit and regression diagnostics

Exposure	Model R^2	Adjusted R^2	Shapiro–Wilk p	Breusch–Pagan p	Maximum VIF	Maximum Cook's distance	Participant	n above 0.20
MEDAS	0.004	−0.183	0.036	0.874	1.006	0.147	10F	0
UPF score	0.009	−0.177	0.037	0.606	1.011	1.210	4M	1
IPAQ-SF, MET-min/week	0.109	−0.058	0.436	0.698	1.530	0.168	9F	0
Adapted Sleep score	0.020	−0.164	0.088	0.600	1.105	0.528	4F	1

Panel B. Leave-one-out influence analyses

Exposure	Primary β	Leave-one-out β range	Leave-one-out p-value range	Sign reversal
MEDAS	0.103	−0.209 to 0.413	0.496–0.962	Yes
UPF score	0.096	−0.450 to 0.263	0.247–0.942	Yes
IPAQ-SF, MET-min/week	-8.42×10^{-4}	-1.10×10^{-3} to -5.98×10^{-4}	0.088–0.428	No
Adapted Sleep score	−0.178	−0.507 to 0.006	0.167–0.985	Yes

Panel C. HC3 and Freedman–Lane inference

Exposure	HC3 SE	HC3 95% CI	HC3 p-value	HC3 p-FDR	Permutation p-value	Permutation p-FDR
MEDAS	0.604	−1.177 to 1.383	0.867	0.874	0.855	0.855
UPF score	0.596	−1.168 to 1.359	0.874	0.874	0.752	0.855
IPAQ-SF, MET-min/week	5.35×10^{-4}	-1.98×10^{-3} to 2.92×10^{-4}	0.135	0.540	0.178	0.712
Adapted Sleep score	0.416	−1.059 to 0.703	0.675	0.874	0.586	0.855

Panel D. Sensitivity to the IPAQ-SF truncation rule

IPAQ-SF score	β per MET-min/week	95% CI	p-value	p-FDR	Partial R^2	Direction concordant
Primary score, durations truncated at 180 min/day	-8.42×10^{-4}	-2.13×10^{-3} to 4.41×10^{-4}	0.183	0.732	0.108	Reference
Original untruncated score	-6.76×10^{-4}	-1.91×10^{-3} to 5.57×10^{-4}	0.262	0.262	0.078	Yes

Panel E. Sensitivity analysis excluding participant 7F (n = 19)

Exposure	β	95% CI	p-value	p-FDR	Partial R^2	Direction concordant
MEDAS	0.238	−0.949 to 1.426	0.675	0.675	0.012	Yes
UPF score	0.124	−0.454 to 0.702	0.653	0.675	0.014	Yes
IPAQ-SF, MET-min/week	-9.69×10^{-4}	-2.20×10^{-3} to 2.58×10^{-4}	0.113	0.452	0.159	Yes
Adapted Sleep score	−0.187	−0.856 to 0.482	0.561	0.675	0.023	Yes

Footnote: All models used the natural-log-transformed *Prevotella/Bacteroides* ratio as the outcome and were adjusted for age and sex, with female as the reference category. Panel A reports diagnostics from the four primary models; Cook's distance values greater than 4/n = 0.20 were used for influence screening. Panel B reports the ranges obtained by excluding each participant in turn; sign reversal indicates that at least one leave-one-out coefficient had the opposite sign from the primary estimate. Panel C reports HC3 heteroscedasticity-consistent inference and two-sided Freedman–Lane tests based on 9,999 permutations. Panel D compares the primary IPAQ-SF score with the score calculated from the original untruncated responses; its sensitivity-analysis p-value constituted a separate multiplicity family. Panel E reports models refitted after temporarily excluding participant 7F, whose microbiome profile had the lowest sequencing depth; the four p-values were adjusted as a separate sensitivity family. No participant was excluded from the primary analyses. CI, confidence interval; FDR, false discovery rate; IPAQ-SF, International Physical Activity Questionnaire–Short Form; MEDAS, Mediterranean Diet Adherence Screener; MET, metabolic equivalent of task; SE, standard error; UPF, ultra-processed food; VIF, variance inflation factor.

Supplementary Table S19. Enrollment targets and stool-specimen workloads across prespecified effect-size, power, significance-threshold, and data-loss scenarios.

Panel A. Anticipated data loss: 10%

Partial R^2	Power	α	Analyzable participants, n	Participants to enroll, n	Specimens with 3 collections, n	Specimens with 4 collections, n
0.01	80%	0.05	779	866	2598	3464
0.03	80%	0.05	256	285	855	1140
0.05	80%	0.05	152	169	507	676
0.10	80%	0.05	73	82	246	328
0.01	90%	0.05	1043	1159	3477	4636
0.03	90%	0.05	342	380	1140	1520
0.05	90%	0.05	202	225	675	900
0.10	90%	0.05	97	108	324	432
0.01	80%	0.0125	1108	1232	3696	4928
0.03	80%	0.0125	364	405	1215	1620
0.05	80%	0.0125	216	240	720	960
0.10	80%	0.0125	104	116	348	464
0.01	90%	0.0125	1418	1576	4728	6304
0.03	90%	0.0125	465	517	1551	2068

0.05	90%	0.0125	275	306	918	1224
0.10	90%	0.0125	132	147	441	588

Panel B. Anticipated data loss: 15%

Partial R^2	Power	α	Analyzable participants, n	Participants to enroll, n	Specimens with 3 collections, n	Specimens with 4 collections, n
0.01	80%	0.05	779	917	2751	3668
0.03	80%	0.05	256	302	906	1208
0.05	80%	0.05	152	179	537	716
0.10	80%	0.05	73	86	258	344
0.01	90%	0.05	1043	1228	3684	4912
0.03	90%	0.05	342	403	1209	1612
0.05	90%	0.05	202	238	714	952
0.10	90%	0.05	97	115	345	460
0.01	80%	0.0125	1108	1304	3912	5216
0.03	80%	0.0125	364	429	1287	1716
0.05	80%	0.0125	216	255	765	1020
0.10	80%	0.0125	104	123	369	492
0.01	90%	0.0125	1418	1669	5007	6676

0.03	90%	0.0125	465	548	1644	2192
0.05	90%	0.0125	275	324	972	1296
0.10	90%	0.0125	132	147	441	588

Panel C. Anticipated data loss: 20%

Partial R^2	Power	α	Analyzable participants, n	Participants to enroll, n	Specimens with 3 collections, n	Specimens with 4 collections, n
0.01	80%	0.05	779	974	2922	3896
0.03	80%	0.05	256	320	960	1280
0.05	80%	0.05	152	190	570	760
0.10	80%	0.05	73	92	276	368
0.01	90%	0.05	1043	1304	3912	5216
0.03	90%	0.05	342	428	1284	1712
0.05	90%	0.05	202	253	759	1012
0.10	90%	0.05	97	122	366	488
0.01	80%	0.0125	1108	1385	4155	5540
0.03	80%	0.0125	364	455	1365	1820
0.05	80%	0.0125	216	270	810	1080
0.10	80%	0.0125	104	130	390	520

0.01	90%	0.0125	1418	1773	5319	7092
0.03	90%	0.0125	465	582	1746	2328
0.05	90%	0.0125	275	344	1032	1376
0.10	90%	0.0125	132	165	495	660

Footnote: The number of participants to enroll was calculated as the ceiling of N_analyzable/(1 − L), where L represents the anticipated proportion of enrolled participants without analyzable data. Total specimen workloads were calculated by multiplying the enrollment target by three or four planned stool collections per participant. Repeated sampling was not assumed to reduce the required number of participants because within-participant temporal variability and correlation could not be estimated from the cross-sectional pilot dataset. The $\alpha = 0.0125$ scenario applies a Bonferroni-adjusted threshold accounting for the four principal exposures.

Supplementary Table S20. Exploratory model-specific sample-size benchmarks derived from pilot exposure-specific effect estimates for alpha diversity, CLR-transformed prespecified genera, and the natural-log-transformed *Prevotella/Bacteroides* ratio.

Panel A. Alpha-diversity outcomes

Outcome	Exposure	Partial R²	Cohen's f²	N, 80% power, α = 0.05	N, 90% power, α = 0.05	N, 80% power, α = 0.0125	N, 90% power, α = 0.0125
Shannon index	MEDAS	0.025654	0.026329	301	402	427	546
	UPF score	0.010609	0.010723	734	982	1,044	1,336
	IPAQ-SF	0.139595	0.162244	51	67	73	92
	Adapted Sleep score	0.025824	0.026509	299	399	424	542
Pielou's evenness	MEDAS	0.058172	0.061765	130	173	184	235
	UPF score	0.013501	0.013686	576	770	818	1,047
	IPAQ-SF	0.049178	0.051722	154	206	219	280
	Adapted Sleep score	0.086806	0.095058	85	113	121	154

Panel B. CLR-transformed prespecified genera

Outcome	Exposure	Partial R²	Cohen's f²	N, 80% power, α = 0.05	N, 90% power, α = 0.05	N, 80% power, α = 0.0125	N, 90% power, α = 0.0125
Bacteroides	MEDAS	0.015603	0.015851	498	665	707	905
Bacteroides	UPF score	0.029124	0.029998	264	353	375	480
Bacteroides	IPAQ-SF	0.039534	0.041161	193	258	275	351
Bacteroides	Adapted Sleep score	0.003559	0.003572	2,200	2,944	3,126	4,002

Prevotella	MEDAS	0.000426	0.000426	18,430	24,671	26,184	33,536
Prevotella	UPF score	0.011343	0.011473	687	918	976	1,249
Prevotella	IPAQ-SF	0.107194	0.120064	68	90	97	123
Prevotella	Adapted Sleep score	0.008567	0.008641	911	1,218	1,294	1,657
Faecalibacterium	MEDAS	0.105400	0.117819	69	92	98	125
Faecalibacterium	UPF score	0.115900	0.131094	62	83	89	113
Faecalibacterium	IPAQ-SF	0.000240	0.000240	32,720	43,802	46,487	59,541
Faecalibacterium	Adapted Sleep score	0.037343	0.038791	205	273	291	372
Bifidobacterium	MEDAS	0.001692	0.001694	4,635	6,204	6,585	8,433
Bifidobacterium	UPF score	0.027011	0.027761	285	381	405	518
Bifidobacterium	IPAQ-SF	0.015913	0.016170	488	652	693	887
Bifidobacterium	Adapted Sleep score	0.088286	0.096836	84	111	119	151
Blautia	MEDAS	0.000004	0.000004	>2,000,000	>2,000,000	>2,000,000	>2,000,000
Blautia	UPF score	0.021356	0.021822	362	484	515	658
Blautia	IPAQ-SF	0.089427	0.098210	82	109	117	149
Blautia	Adapted Sleep score	0.000331	0.000331	23,698	31,724	33,668	43,123
Roseburia	MEDAS	0.034255	0.035469	224	299	318	406

Roseburia	UPF score	0.152125	0.179418	46	61	66	83
Roseburia	IPAQ-SF	0.033899	0.035089	226	302	321	411
Roseburia	Adapted Sleep score	0.056601	0.059997	133	178	190	242
Ruminococcus	MEDAS	0.004315	0.004334	1,813	2,427	2,577	3,299
Ruminococcus	UPF score	0.033457	0.034615	229	306	326	416
Ruminococcus	IPAQ-SF	0.072463	0.078124	103	137	146	187
Ruminococcus	Adapted Sleep score	0.014777	0.014998	526	703	747	956
Akkermansia	MEDAS	0.079474	0.086335	93	124	133	169
Akkermansia	UPF score	0.210133	0.266037	32	42	46	58
Akkermansia	IPAQ-SF	0.099081	0.109978	74	98	105	134
Akkermansia	Adapted Sleep score	0.099947	0.111046	73	97	104	132

Panel C. Natural-log-transformed *Prevotella*/*Bacteroides* ratio

Outcome	Exposure	Partial R^2	Cohen's f^2	N, 80% power, α = 0.05	N, 90% power, α = 0.05	N, 80% power, α = 0.0125	N, 90% power, α = 0.0125
ln(*Prevotella*/*Bacteroides*)	MEDAS	0.002041	0.002045	3,840	5,140	5,456	6,987
ln(*Prevotella*/*Bacteroides*)	UPF score	0.007309	0.007362	1,069	1,430	1,518	1,944
ln(*Prevotella*/*Bacteroides*)	IPAQ-SF	0.107973	0.121042	67	89	96	122
ln(*Prevotella*/*Bacteroides*)	Adapted Sleep score	0.018487	0.018835	419	560	596	762

Footnote: Each row represents a separate age- and sex-adjusted linear regression model. Partial R^2 is the exposure-specific effect estimate obtained from the pilot analysis and was converted to Cohen's f^2 as f^2 = partial R^2/(1 − partial R^2). N denotes the minimum required number of analyzable participants under the specified power and significance-level scenario. The α = 0.0125 scenario applies a Bonferroni-adjusted threshold accounting for the four principal exposures. Alpha-diversity effect estimates were obtained from analyses using the full-precision Shannon diversity and Pielou's evenness values independently recalculated from the exported OTU count table. Genus-level outcomes were represented by CLR coordinates, whereas the Prevotella/Bacteroides outcome was natural-log-transformed after addition of the prespecified pseudocount. The near-zero partial R^2 for the MEDAS–Blautia model produced requirements exceeding 2,000,000 analyzable participants and is therefore reported as >2,000,000. These estimates are exploratory, model-specific benchmarks derived from a small pilot sample. They should not be interpreted as definitive sample-size recommendations or as formal power calculations for longitudinal or PERMANOVA-based analyses; the principal prospective planning range was instead based on assumed partial R^2 values of 0.03–0.05. CLR, centered log-ratio; IPAQ-SF, International Physical Activity Questionnaire–Short Form; MEDAS, Mediterranean Diet Adherence Screener; OTU, operational taxonomic unit; UPF, ultra-processed food.

Figures

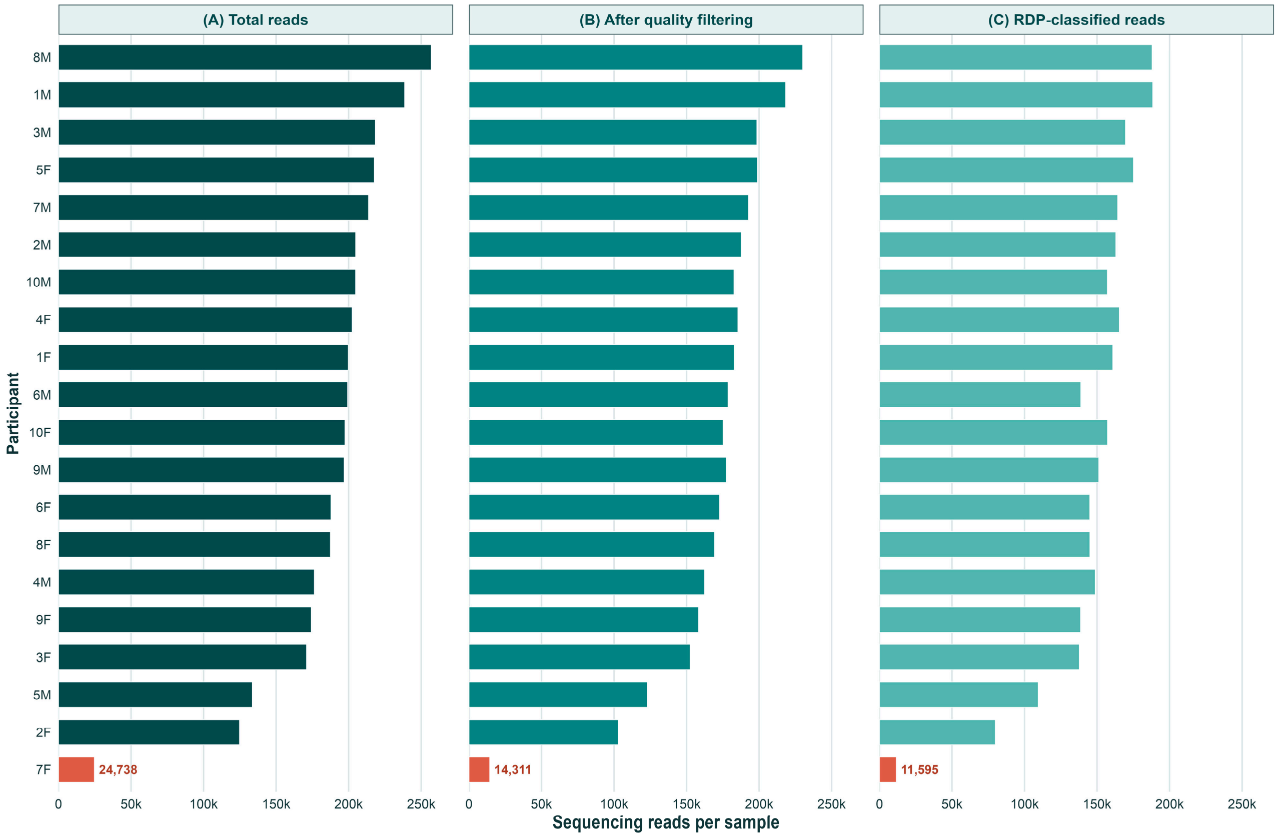

Supplementary Figure S1. Participant-level sequencing yield and taxonomic-classification performance. The three panels show (A) total reads, (B) reads retained after quality filtering, and (C) reads assigned using the Ribosomal Database Project classifier for each of the 20 participant-level microbiome profiles. Participants are presented in the same order across panels and ranked by decreasing total read count. Participant 7F, highlighted in coral, had the lowest sequencing depth, with 24,738 total reads, 14,311 quality-filtered reads, and 11,595 RDP-classified reads, and was retained in the analytical dataset. F, female; M, male; RDP, Ribosomal Database Project.

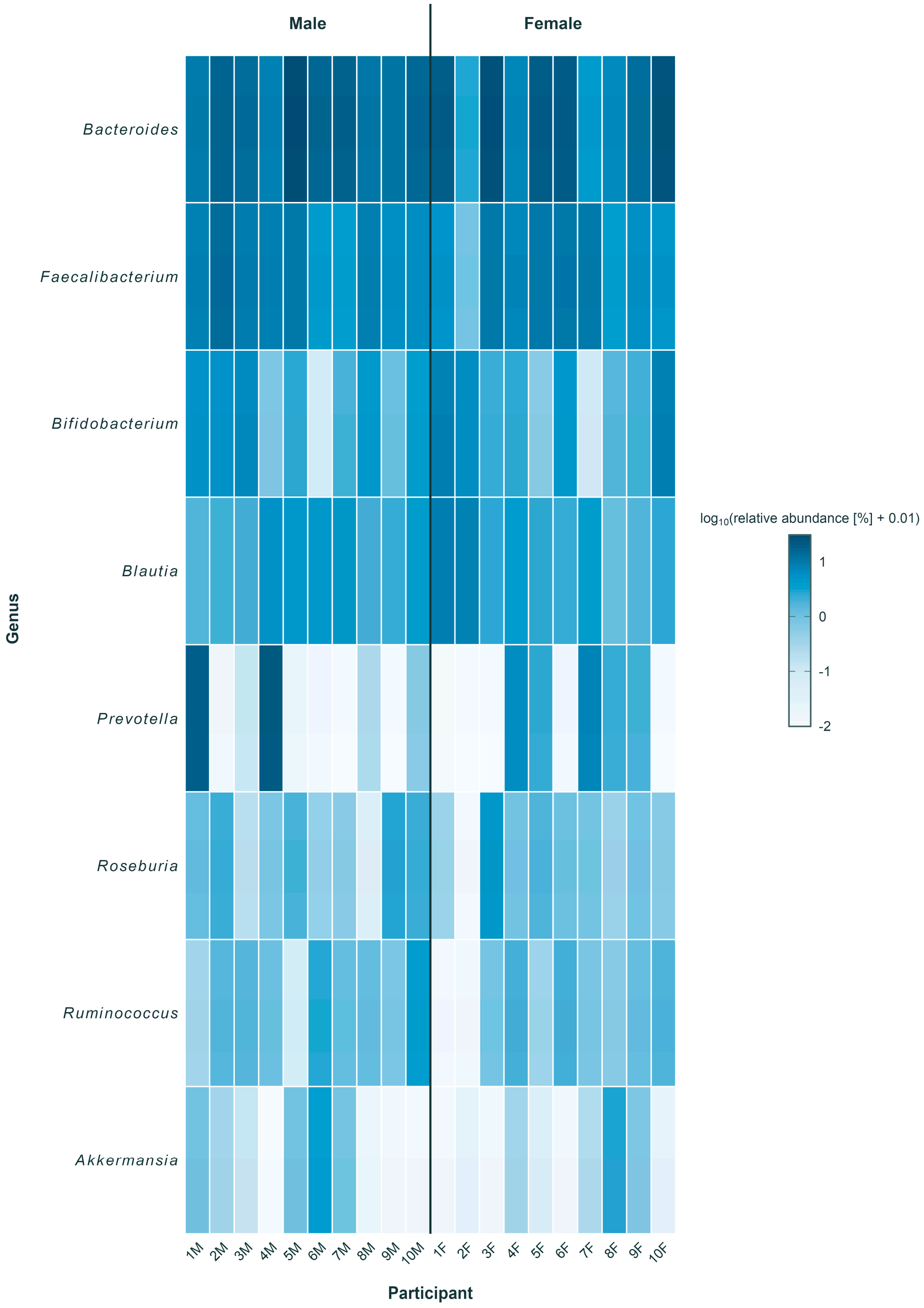
Male
Female
Bacteroides
Faecalibacterium
Bifidobacterium
Blautia
Prevotella
Roseburia
Ruminococcus
Akkermansia
Genus
1M
2M
3M
4M
5M
6M
7M
8M
9M
10M
1F
2F
3F
4F
5F
6F
7F
8F
9F
10F
Participant
log10(relative abundance [%] + 0.01)
1
0
-1
-2

Supplementary Figure S2. Participant-level distribution of the eight prespecified bacterial genera. The heatmap displays the genus-level relative abundances of the eight prespecified genera across the 20 participants, expressed as $\log_{10}$(relative abundance [%] + 0.01). The addition of 0.01 percentage points was used exclusively to permit visualization of zero abundances and was not used in the inferential analyses. Participants are ordered by sex and participant identifier. Darker blue shades indicate higher relative abundance. Sex grouping is presented for descriptive visualization only and does not represent an inferential comparison. F, female; M, male.

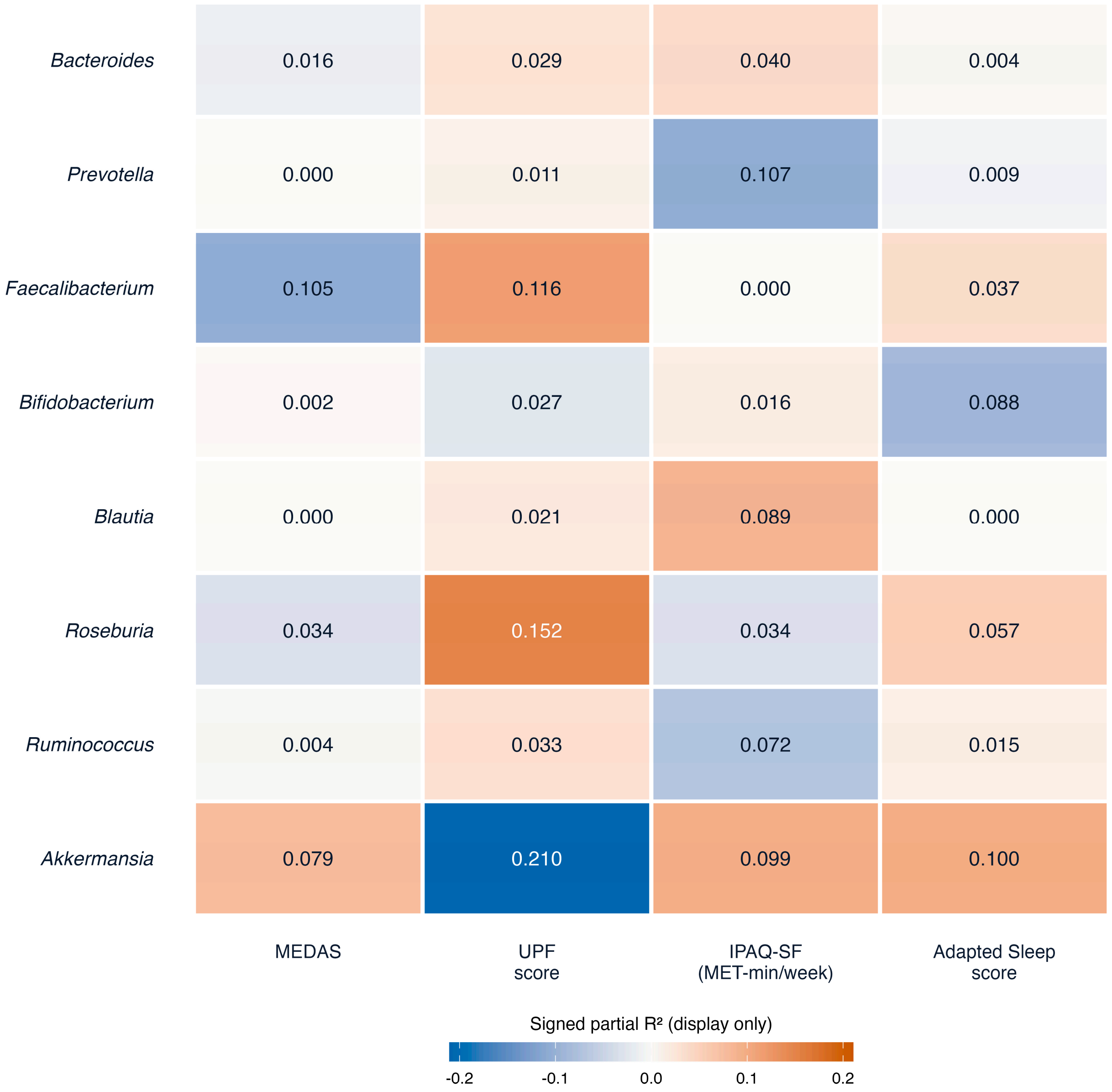

Supplementary Figure S3. Direction and magnitude of age- and sex-adjusted associations between dietary and lifestyle exposures and the centered log-ratio coordinates of the eight prespecified bacterial genera. Each cell reports the partial R^2 attributable to the exposure in a separate ordinary least-squares model adjusted for age and sex. Cell colors encode the direction of the corresponding exposure coefficient—blue for negative and orange for positive associations—whereas color intensity represents the magnitude of the partial R^2. The signed partial R^2 was used exclusively for graphical display and does not constitute a separate inferential statistic. None of the associations was statistically supported after false-discovery-rate correction. CLR, centered log-ratio; IPAQ-SF, International Physical Activity Questionnaire–Short Form; MEDAS, Mediterranean Diet Adherence Screener; MET, metabolic equivalent of task; R^2, coefficient of determination; UPF, ultra-processed food.

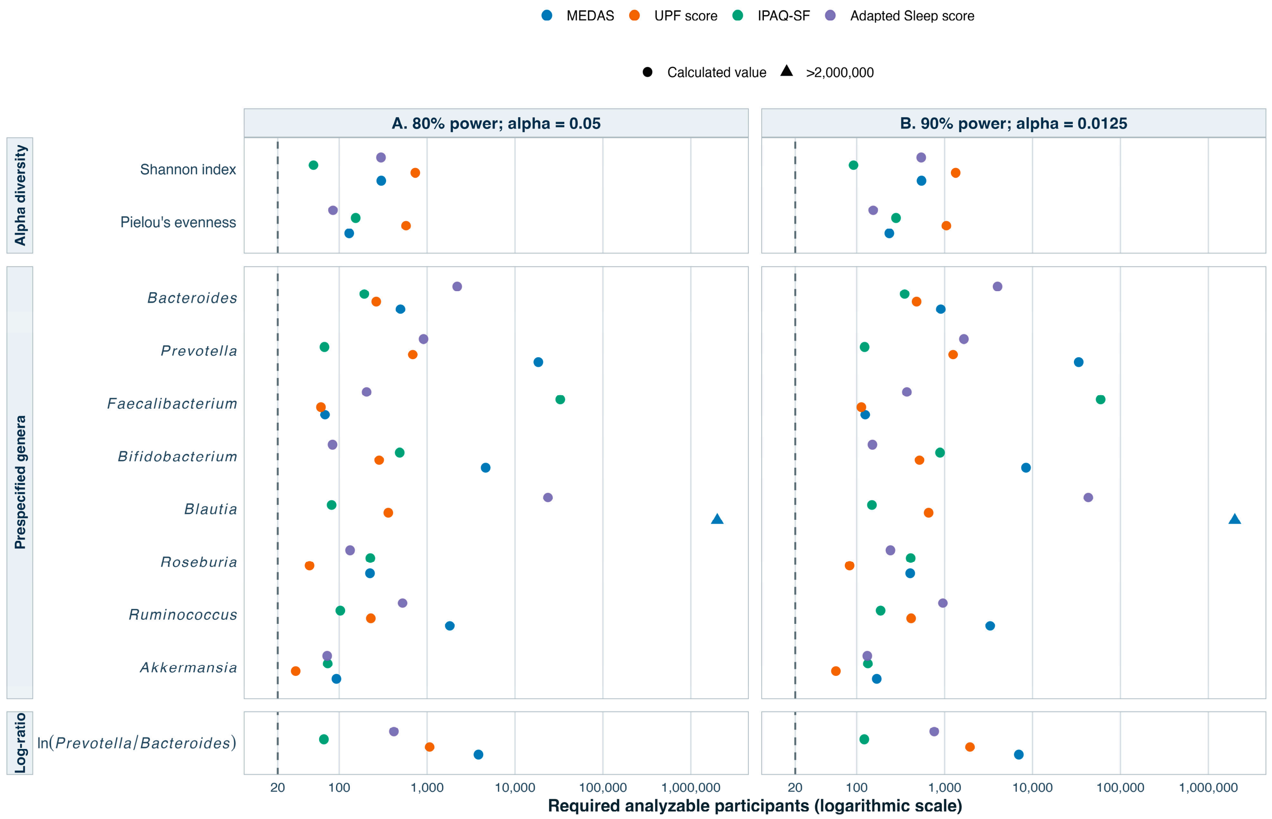

Supplementary Figure S4. Model-specific sample-size benchmarks derived from exposure-specific partial R^2 values observed in the pilot linear-regression analyses. Panels A and B show the numbers of analyzable participants required to achieve 80% power at $\alpha = 0.05$ and 90% power at $\alpha = 0.0125$, respectively. The 44 models comprise eight alpha-diversity models, 32 CLR-transformed genus-level models, and four natural-log-transformed Prevotella/Bacteroides ratio models. Each point represents one exposure–outcome model; outcomes are identified by row and exposures by color. The vertical dashed line indicates the present pilot sample size (n = 20), and the horizontal axis is displayed on a logarithmic scale. Circles denote calculated values, whereas triangles denote effect estimates for which the calculated requirement exceeded 2,000,000 analyzable participants or the predefined numerical search limit. These pilot-derived estimates are presented exclusively as exploratory, model-specific benchmarks and should not be interpreted as the principal prospective planning range or as formal power calculations for longitudinal or PERMANOVA-based analyses. CLR, centered log-ratio; IPAQ-SF, International Physical Activity Questionnaire–Short Form; MEDAS, Mediterranean Diet Adherence Screener; UPF, ultra-processed food.

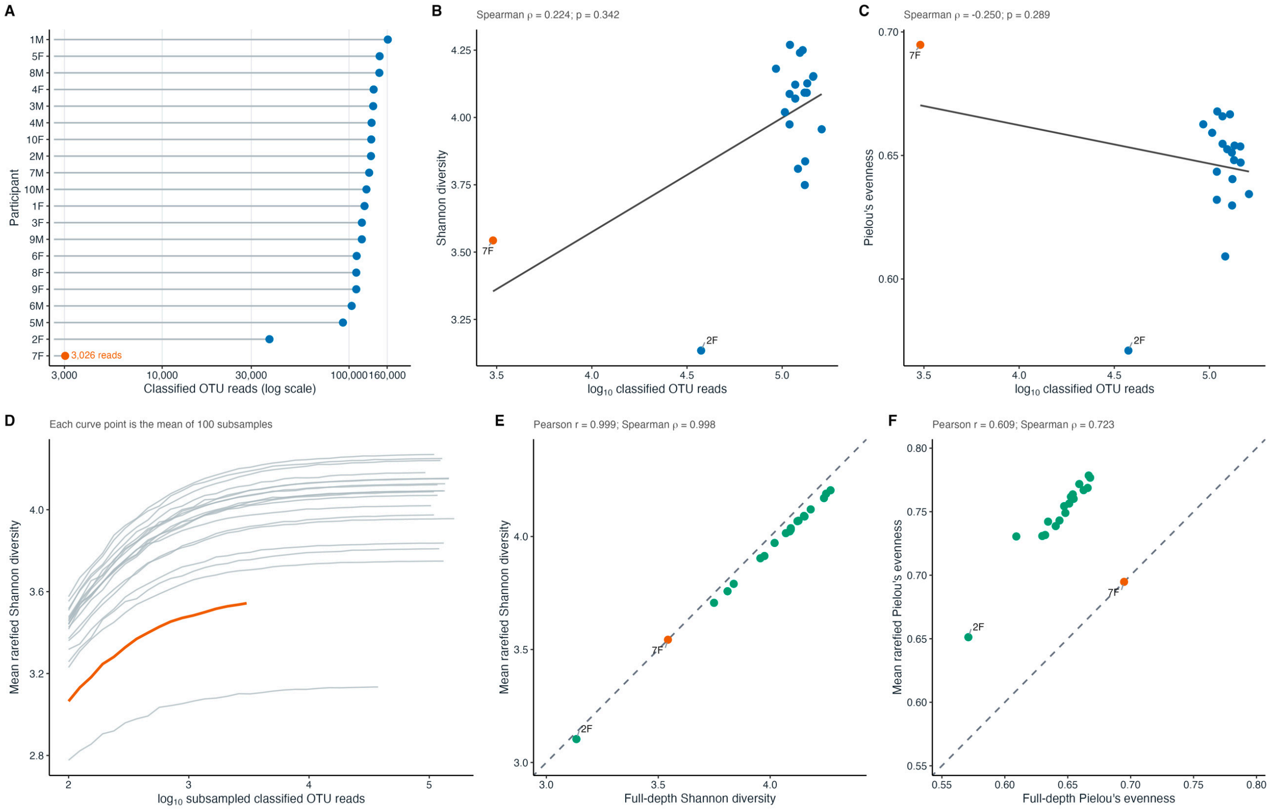

Supplementary Figure S5. Sequencing-depth diagnostics for independently recalculated OTU-level alpha diversity. (A) Participant-specific classified OTU-read depths, ordered from lowest to highest and displayed on a logarithmic scale. Participant 7F, who had the minimum depth of 3026 reads, is highlighted in orange. (B,C) Relationships of Shannon diversity and Pielou's evenness with log10-transformed classified OTU-read depth. Solid lines represent ordinary least-squares fits for visualization, whereas the displayed ρ values and p-values correspond to Spearman rank correlations. Participants 7F and 2F are labelled because they represent the lowest sequencing depth and the lowest alpha-diversity values, respectively. (D) Individual Shannon rarefaction curves generated by repeated subsampling without replacement. Each plotted value below the participant's full sequencing depth represents the mean of 100 independent subsamples; participant 7F is highlighted in orange and the remaining participants are shown in grey. (E,F) Concordance between full-depth and mean rarefied Shannon diversity and Pielou's evenness after 1000 rarefactions to the common depth of 3026 classified OTU reads per participant. Dashed diagonal lines represent identity; participant 7F is highlighted in orange, whereas the remaining participants are shown in green. Rarefaction was used exclusively as a sequencing-depth sensitivity analysis and did not replace the full-depth primary calculations. F, female; M, male; OTU, operational taxonomic unit.
